# Participating in a Citizen Science Monitoring Program: Implications for Environmental Education

**DOI:** 10.1371/journal.pone.0131812

**Published:** 2015-07-22

**Authors:** Simone Branchini, Marta Meschini, Claudia Covi, Corrado Piccinetti, Francesco Zaccanti, Stefano Goffredo

**Affiliations:** 1 Marine Science Group, Citizen Science Lab, Department of Biological, Geological and Environmental Sciences, Section of Biology, University of Bologna, Via F. Selmi 3, I-40126, Bologna, Italy, European Union; 2 Laboratory of Fisheries and Marine Biology at Fano, University of Bologna, Viale Adriatico 1/N, I-61032, Fano (PU), Italy, European Union; University of California Santa Cruz, UNITED STATES

## Abstract

Tourism is of growing economical importance to many nations, in particular for developing countries. Although tourism is an important economic vehicle for the host country, its continued growth has led to on-going concerns about its environmental sustainability. Coastal and marine tourism can directly affect the environment through direct and indirect tourist activities. For these reasons tourism sector needs practical actions of sustainability. Several studies have shown how education minimizes the impact on and is proactive for, preserving the natural resources. This paper evaluates the effectiveness of a citizen science program to improve the environmental education of the volunteers, by means of questionnaires provided to participants to a volunteer-based Red Sea coral reef monitoring program (STEproject). Fifteen multiple-choice questions evaluated the level of knowledge on the basic coral reef biology and ecology and the awareness on the impact of human behaviour on the environment. Volunteers filled in questionnaires twice, once at the beginning, before being involved in the project and again at the end of their stay, after several days participation in the program. We found that the participation in STEproject significantly increased both the knowledge of coral reef biology and ecology and the awareness of human behavioural impacts on the environment, but was more effective on the former. We also detected that tourists with a higher education level have a higher initial level of environmental education than less educated people and that the project was more effective on divers than snorkelers. This study has emphasized that citizen science projects have an important and effective educational value and has suggested that tourism and diving stakeholders should increase their commitment and efforts to these programs

## Introduction

Tourism is a cross-cutting sector, involving a large diversity of services and professions, linked to many other economic activities and policy areas. For this reason, tourism is one of the most important forces shaping our world, which makes it worth devoting attention to [1; 2]. Tourism is of growing economical importance to many nations and is recognized as the largest export earner in the world and as an important provider of foreign exchange and employment [2; 3]. To date, the tourism industry represents 9% of global GDP, which corresponds to USD 1.4 trillion in international exports [4]. According to the United Nations World Tourism Organization, despite occasional shocks, such as the global economical crisis, international tourist arrivals have shown virtually uninterrupted growth (from 528 million in 1995 to 703 million in 2002 and 1085 million in 2013) and they are expected to increase by 3.3% per year from 2010 to 2030, reaching 1.8 billions by 2030. In particular, visitors in emerging destinations (+ 4.4% per year) are expected to increase at twice the rate of those in advanced economies (+ 2.2% per year) [4; 5].

For these reasons, developing countries are encouraged to use tourism as a means of economic development that wreaks less damage than extractive industries [6] and can be used to create many employment opportunities for the local population and to generate revenue for other developmental activities [7]. In Egypt, tourism generates an estimated USD 7.8 billion annually (equivalent to 11.3% of the national gross domestic product) and represents 47.8% of international exports, providing employment for 12.6% of the national work force [8; Egyptian Tourist Authority, personal communication]. Although the Great Pyramids of Giza and The Nile River are some of the world's most iconic touristic attractions, the Red Sea coastal zone attracts great numbers of tourists. In the period 2010–2013, more than 30 million people arrived from all over the world to visit the coral reefs of the Egyptian Red Sea, providing growing demand for touristic infrastructures and delivering important foreign revenue to the regional and national economy (according to CAPMAS–Egyptian Central Agency for Public Mobilization and Statistics; www.capmas.gov.eg).

Although tourism is an important economic vehicle for the host country, its continued growth has led to on-going concerns about its environmental sustainability and the increasing criticism on the negative impacts of tourism began in the 1980s [9–15]. In particular, coastal and marine tourism can directly affect the environment through localized pollution, resource depletion, habitat loss, conversion and habitat and wildlife disturbance. In addition, these impacts have been shown to reduce recreational enjoyment, decreasing tourism business [16; 17]. Physical development of resorts, consumption of fuel by buildings, aircraft, trains, buses, taxis and cars, overuse of water resources, oil-spills, pollution by vehicle emissions, sewage, litter and boat anchors and groundings have caused ecosystem degradation. Several studies have shown how the direct presence and activities of the tourists along the shores have a negative impact on the environment [18–21].

Although all coastal habitats are affected by tourism [22], coral reef habitats seem more susceptible to an uncontrolled and unplanned tourist flow. Recreational marine activities affect corals in many ways, such as trampling, breakages, physical contact with organisms, sediment resuspension, behavioural changes among marine life due to food offerings, animal harassment, trash and debris production. For example, snorkelers and SCUBA divers can inadvertently damage corals by clambering over them, by kicking them accidentally with their fins, or by stirring up silt that suffocates them (e.g. [18; 19]). They may unintentionally damage stony corals and other benthic reef organisms by breaking their skeletons and abrading their tissues. Also other activities, not properly related with snorkelling or SCUBA diving, are reasonably considered dangerous for the environment, such as shell collecting, feeding fish and buying or collecting “marine” souvenirs.

The tourism sector needs practical actions to ensure sustainability. These actions must be integrated into all steps of tourism planning and coordinated at community or regional level, and applied to all forms of tourism in all types of destinations. The importance of raising environmental awareness and education among tourists is emphasized by Lansing and De Vries [2]. Education minimizes the impact on and is proactive for preserving the natural resources [18, 23–26]. Medio et al. [27] showed that divers did less damage after a 45-minute illustrated dive briefing covering reef biology, contacts caused by divers and the concept of a protected area. Divers were shown the different forms of live reef cover and non-living substrate, such as rock and dead coral, to illustrate areas of the reef that could be touched without damage it. Also, Rouphael and Inglis [28] suggested that the probability of divers coming into contact with corals is determined also by their awareness of the environmental consequences of their actions. Barradas et al. [29] state that no sustainable actions (such as: limitation of water consumption, wasting and pollution reduction, environmental limitations) are effective without a good educational program. Nevertheless, dive companies often give briefings that last only a few minutes and in many instances they do not include sustainability tips [16].

This paper evaluates the effectiveness of a citizen science program to improve the environmental education of the volunteers, by involving them in a practical biodiversity monitoring program. Through a specific questionnaire, the level of environmental education of volunteers was assessed before the participation in a coral reef biodiversity monitoring program and after several participations to it.

## Methods

### STE project

“STE: Scuba Tourism for the Environment” (STE) is a volunteer-based coral reef biodiversity monitoring program based, which is being implemented in three countries facing the Red Sea: Egypt, Sudan and Saudi Arabia. The main project goals have been to: 1) collect information on the presence and abundance of key coral reef taxa, by using the skills of non-specialist volunteers, and 2) improve their environmental awareness, by engaging them in a practical conservation program. The “recreational monitoring” approach [30; 31] used in STEproject allowed volunteers to carry out normal recreational activities during their reef visits and ensured the reliability of gathered data through standardized data collection. Without forcing volunteers to follow pre-selected transects or strict survey protocols, this approach guaranteed the enjoyment of the volunteer in project participation and allowed the engagement of a relevant number of volunteers.

Since 2007, user-friendly questionnaires distributed to volunteer recreational divers and snorkelers were used to gather key information on coral reef ecosystem health. During seven years of data collection (2007–2013), 14,502 volunteers were involved in the project resulting in 29,312 completed questionnaires. The data collected was useful to detect environmental status trends and inform the local environmental managers on the effectiveness of current management actions and how to direct future efforts [32].

The research team held training courses for professional divers before the beginning of the project and yearly throughout the project. The research team trained professional divers about the project’s objectives and methods, including taxa identification and data recording (the training program consisted of lectures, video, slideshows, and field identification). Topics such as biodiversity and its application in assessing environmental change caused by natural and anthropogenic pressures were covered. Subsequently in the field, divemasters and SCUBA instructors, with the help of students of the research team, briefed the divers, providing information on the habitat features, the species that may be encountered, and tips on how to minimize the impact of diving activities on coral reefs. They then assisted the volunteers during data collection and were available for consultation in case of difficulties with species identification, providing more information about environmental and ecological issues (see [32], for detailed training procedure).

The questionnaire contained an initial section providing guidance for limiting anthropogenic impacts on the reef and throughout the vacation period (see [32], for the questionnaire). This section could be torn off and conserved by volunteers after their participation in the project.

### Environmental education: evaluation questionnaire

**Fig 1:**
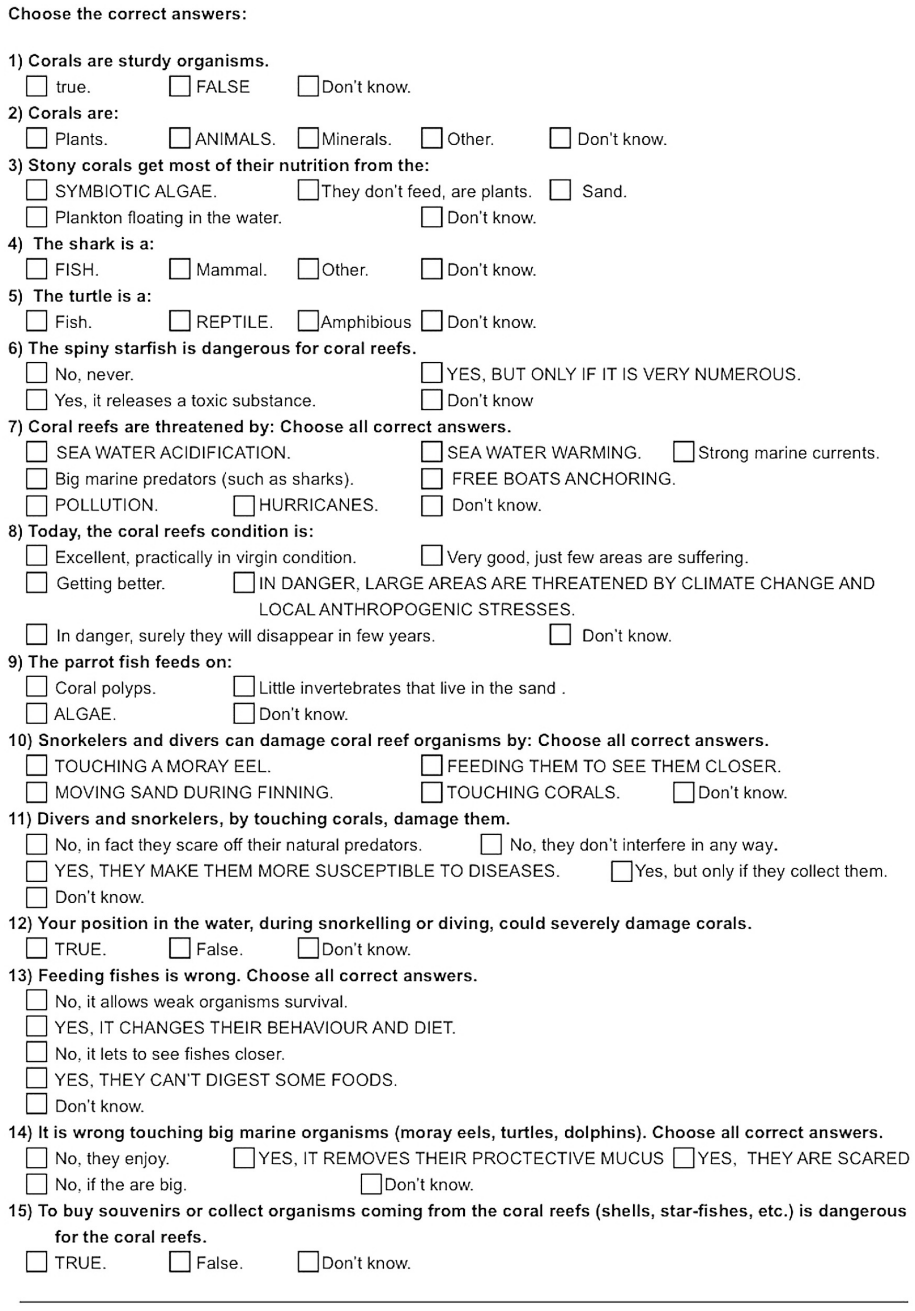
Environmental education evaluation questionnaire. The figure show the section dedicated to the evaluation of the level of environmental education. The answers in capital letters show the correct answer. *STE project-Citixen Science Lab*,*Marine Science Group*, *Dipartimento di Biologia E*.*S*., *Universita di Bologna*, *Via Selmi 3*,*40126 Bologno*, *italy* www.marinesciencegroup.org

To verify the effectiveness of the project in increasing the environmental education of the volunteers, an additional questionnaire was created and provided in Egypt to a subset of volunteers during the years 2012 and 2013. This questionnaire consisted of two sections. The first section aimed to collect personal and demographic data of the volunteer to identify factors that could influence the initial level of environmental education and its improvement after the project (Table 1): 1) gender (male, female); age (five age categories); level of education (five categories, according to Italian level of education); diving qualification (six categories, according to World Recreational Scuba Training Council–WRSTC). An additional question assessed if the volunteer already participated in the project: “How many questionnaires of the STEproject did you fill out until today?”. A statement declared that the survey was used for research purpose. The second section evaluated the level of environmental education. It contained 15 multiple-choice questions. These questions contained two different kinds of issues. The first set of questions (9 questions, from number 1 to number 9; Fig 1) covered the knowledge on the basic coral reef biology and ecology, hereafter called reef biology questions. The second set of questions (6 questions, from number 10 to number 15; Fig 1) dealt with the awareness on the impact of human behaviour on the environment, hereafter called human impact questions. There was only one correct answer, except when explicitly stated with the sentence “Choose all answers that you consider correct”. We developed the questions tailored to a tropical marine environment and based on the content that the STEproject was expected to cover. Members of the STEproject research group working in the field provided the questionnaire to the volunteers twice, once at the beginning, before being involved in the project and again at the end of their stay, after several days participation in the program, so that every volunteer filled out the same questionnaire twice.

**Table 1:** Volunteers’ personal and demographic data collected to identify factors that could influence the initial level of environmental awareness and its improvement after the project.

Factor	Categories
*Gender*	1: Female
	2: Male
*Age*	1: < 15 years old
	2: 16–30 years old
	3: 31–45 years old
	4: 46–60 years old
	5: > 61 years old
*Level of education*	1: Compulsory School
	2: High School
	3: Bachelor Degree (B.Sc.)
	4: Master Degree (M.Sc.)
	5: Doctorate of Philosophy (Ph.D.)
*Diving qualification*	1: None
	2: Open Water Diver (O.W.D.)
	3: Advanced Open Water Diver (A.O.W.D.)
	4: Rescue Diver
	5: Divemaster
	6: Instructor

Participants (or parents/guardians in case of minors) gave their consent by signing a declaration inserted in the questionnaires. STEproject and its consent acquisition procedure have received the approval of Bioethics Committee of the University of Bologna.

The data were anonymously analysed. The second section was analysed giving a score for each answer. The score was negative if the answer was wrong, positive if it was correct and zero if it was “*I don’t know*”. The value of the score of each question was calculated so that the sum of all correct answers would be +1 and the sum of all the wrong answers -1. During the elaboration, we analysed and compared the overall questionnaire score (15 questions), the score of the reef biology questions (9 questions) and the score of the human impact questions (6 questions). For this reason we standardized all the scores ranging from 0 (all answers wrong) to 10 (all answers correct). We performed a volunteer-level analysis by comparing, for each volunteer, the total scores of the pre-questionnaire with those of the post-questionnaire, for all volunteers together and then splitting the volunteers according to their personal and demographic data (gender, age, level of education, diving qualification; Table 1).

Differences in the mean score of questionnaires were examined either by T-student test or by one-way analysis of variances (ANOVA), when the factors that could influence the initial level of environmental education and its improvement after the project were defined by more than two groups or categories.

## Results

In two years a total of 212 volunteers completed 424 questionnaires. Most of the volunteers were men (129, 60.8%), but there was a considerable participation of women (83, 39.2%). The most frequent age group comprised 31 to 45-year-olds (84, 39.6%), followed by 46 to 60-year-olds (66, 31.1%) and 16 to 30-year-olds (44, 20.8%). The groups under 15 years-old (10, 4.7%) and over 60 years-old (8, 3.8%) had low numbers and were less surveyed. The level of education of the majority of volunteers was high school (95, 44.8%), 45 volunteers (21.2%) were master graduated, 42 (19.8%) completed the compulsory school, 27 (12.7%) had a bachelor degree and 3 were Doctors of Philosophy. A hundred and thirty-five (63.7%) volunteers were snorkelers, 60 (28.3%) were recreational divers (20 open water divers, 9.4%; 32 advanced open water divers, 15.1%; and 8 rescue diver, 3.8%) and 17 (8.0%) were professional divers (5 divemasters, 2.4%; 12 instructors, 5.7%). No volunteers had already participated in the STE project before filling the first environmental awareness evaluation questionnaire.

**Fig 2:**
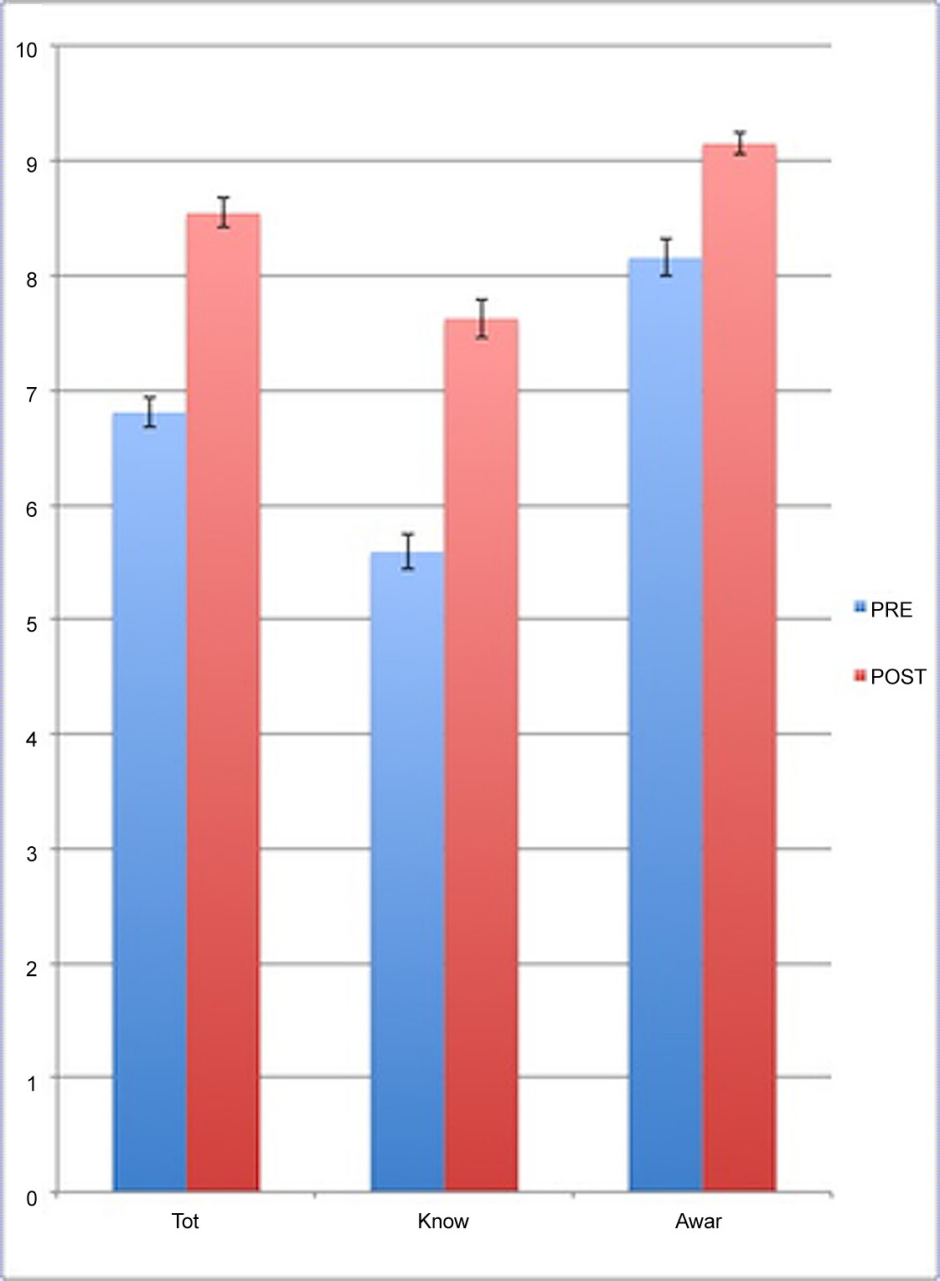
Mean score of the environmental education evaluation questionnaire. *Tot* represents the mean score of the overall questionnaires, *Know* represents the mean score of the reef biology questions and *Awar* represents the mean score of the human impact questions. Error bars are 95% confidence intervals (CI), N = 212.

The comparison between the score of the pre-questionnaire with those of the post-questionnaire showed 192 cases (90.6%) where the post-questionnaire had a higher score than the first one, 12 cases (5.7%) where the score of the two questionnaires were equal and 8 cases (3.8%) where the post-questionnaire had a lower score than the first one. For the overall questionnaire, the reef biology and the human impact questions, the mean score of the post-questionnaire resulted significantly higher than that of the pre-questionnaire (respectively T = -18.959, *p* < 0.01; T = -17.385 *p* < 0.01; and T = -10.132, *p* < 0.01; Fig 2)

Both males and females showed the mean score of the post-questionnaire significantly higher than that of the pre-questionnaire for the overall questionnaire, the reef biology and the human impact questions (Table 2), without significant differences between genders (Table 3).

**Table 2:** Results of T-student test and the percent increase between the score of the post-questionnaire and the score of the pre-questionnaire for the overall questionnaire, the reef biology and the human impact questions.

			*Overall questionnaire*			*Knowledge questions*			*Awareness questions*		
		*df*	T	*p*	%	T	*p*	%	T	*p*	%
*Gender*	Female	166	-12.500	< 0.001	20.6	-11.129	< 0.001	26.1	-6.237	< 0.001	10.5
	Male	254	-14.300	< 0.001	19.1	-13.331	< 0.001	27.	-8.025	< 0.001	11.5
*Age*	< 15 y.o.	18	-3.813	0.001	16.1	-2.722	0.014	20.4	-3.500	0.003	11.1
	16–30 y.o.	86	-7.374	< 0.001	18.9	-7.365	< 0.001	28.2	-3.428	0.001	7.3
	31–45 y.o.	166	-13.171	< 0.001	20.7	-11.957	< 0.001	28.6	-6.093	< 0.001	10.8
	46–60 y.o.	130	-10.743	< 0.001	19.6	-10.493	< 0.001	25.0	-9.707	< 0.001	13.0
	> 61 y.o.	14	-3.086	0.011	17.9	-3.111	0.008	21.5	-3.874	0.002	13.3
*Level of education*	Compulsory School	82	-8.435	< 0.001	19.1	-7.078	< 0.001	24.3	-4.912	< 0.001	12.8
	High School	186	-13.746	< 0.001	19.6	-11.733	< 0.001	27.1	-7.119	< 0.001	10.1
	B.Sc.	52	-5.610	< 0.001	21.6	-6.263	< 0.001	28.9	-3.151	0.003	12.2
	M.Sc.	90	-8.022	< 0.001	19.2	-8.421	< 0.001	26.1	-4.614	< 0.001	10.5
	Ph.D.	4	-15.76	< 0.001	22.8	**-2.226**	**0.086**	37.1	**-1.131**	**0.321**	5.5
	Under-grad.	324	-8.825	< 0.001	19.8	-15.010	< 0.001	26.7	-8.938	< 0.001	11.1
	Post-grad.	96	-2.311	0.022	19.4	-8.735	< 0.001	26.8	-4.727	< 0.001	10.2
*Diving qualification*	None	270	-14.080	< 0.001	19.7	-14.055	< 0.001	27.2	-7.716	< 0.001	10.3
	O.W.D.	38	-6.068	< 0.001	21.6	-5.911	< 0.001	29.1	-3.371	0.002	11.6
	A.O.W.D.	60	-9.722	< 0.001	20.1	-6.028	< 0.001	25.3	-5.871	< 0.001	13.7
	RD	14	-3.685	0.003	15.3	**-2.090**	**0.055**	22.6	**-1.118**	**0.282**	6.5
	Divemaster	8	-4.470	0.004	31.2	-6.094	< 0.001	32.7	-2.708	0.027	29.1
	Instructor	22	-4.533	< 0.001	13.3	-4.462	< 0.001	20.9	**-0.811**	**0.426**	3.4
	Snorkelers	270	-14.08	< 0.001	19.7	-14.055	< 0.001	27.2	-7.716	< 0.001	10.3
	Divers	150	-13.421	< 0.001	19.7	-10.181	< 0.001	25.9	-6.589	< 0.001	11.9

The *Overall questionnaire* column represents the analyses performed on the 15 questions, the *Knowledge questions* column represents the analyses performed on the 9 questions on the knowledge on the basic coral reef biology and ecology and the *Awareness questions* column represents the analyses performed on the 6 questions on the awareness on the impact of human behaviour on the environment. In the table are represented the value of the T-student Test (*T*) and the level of significance (*p*). The non-significant differences of the T-student test are in bold. *%* represents the percent increase between the score of the post-questionnaire and the score of the pre-questionnaire for the overall questionnaire.

**Table 3:** Results of T student test or ANOVA test among the categories and groups for the mean score of the overall questionnaire, for the reef biology and the human impact questions, in the pre-, in the post-questionnaire and the its increase between the pre- and the post-questionnaire.

					Pre questionnaire		Post questionnaire		Increase	
			Test	*df*	value	*p*	value	*p*	value	*p*
*Gender*		Overall	T-student	210	0.400	0.680	0.968	0.334	0.454	0.650
		Know	T-student	210	0.477	0.634	-0.374	0.709	-0.673	0.502
		Awar	T-student	210	0.980	0.328	0.793	0.429	-0.508	0.612
*Age*		Overall	ANOVA (F)	4	0.720	0.579	0.831	0.507	1.138	0.340
		Know	ANOVA (F)	4	0.997	0.410	0.584	0.675	0.893	0.469
		Awar	ANOVA (F)	4	0.642	0.633	0.413	0.799	1.316	0.265
*Level of education*	*all categories*	Overall	ANOVA (F)	4	1.636	0.166	1.429	0.225	1.240	0.295
		Know	ANOVA (F)	4	0.816	0.517	1.340	0.256	0.639	0.636
		Awar	ANOVA (F)	4	1.583	0.180	1.750	0.140	0.418	0.796
	*under-graduate*	Overall	T-student	210	**-2.311**	**0.022**	-1.104	0.271	1.175	0.243
	*vs*. *post-graduate*	Know	T-student	210	-0.036	0.971	-0.62	0.951	-0.026	0.979
		Awar	T-student	210	-0.276	0.783	0.282	0.778	0.440	0.660
*Diving qualification*	*all categories*	Overall	ANOVA (F)	5	0.685	0.635	**2.283** *	**0.048** *	0.648	0.663
		Know	ANOVA (F)	5	0.748	0.588	0.993	0.423	0.689	0.633
		Awar	ANOVA (F)	5	**2.44** †	**0.036** †	1.000	0.419	**3.553** ‡	**0.004** ‡
	*snorkelers vs*.	Overall	T-student	210	-1.251	0.212	**-2.906**	**0.004**	-1.294	0.199
	*divers*	Know	T-student	210	-0.721	0.472	-0.157	0.875	0.417	0.677
		Awar	T-student	210	0.973	0.332	0.358	0.721	-0.768	0.443

* LSD post-hoc tests showed a significant difference between the category *Snorkelers* and the categories *Open Water Divers and Instructors* (*p* = 0.008; 0045).

^**†**^ LSD post-hoc tests showed a significant difference between the category *Open Water Divers* and the category *Instructors* (*p* = 0.044) and between the category *Divemasters* and the categories *Snorkelers*, *Advanced Open Water Divers*, *Rescue Divers and Instructors* (*p* = 0.010; 0.042; 0.014; 0.002).

^‡^ LSD post-hoc tests showed a significant difference between the category *Advanced Open Water Divers* and the category *Instructors* (*p* = 0.019) and between the category *Divemasters* and *Snorkelers*, *Open Water Divers*, *Advanced Open Water Divers*, *Rescue Divers and Instructors* (*p* = 0.001; 0.004; 0.010; 0.002; < 0.001). The significant differences are in bold.

According to age, all categories showed the mean score of the post-questionnaire significantly higher than that of the pre-questionnaire for the overall questionnaire, the reef biology and the human impact questions (Table 2), without significant differences among the categories (Table 3).

According to the level of education, all categories showed the mean score of the post-questionnaire significantly higher than that of the pre-questionnaire for the overall questionnaire, the reef biology and the human impact questions (with the only exception of the category “*Doctor of Philosophy*” for the reef biology and the human impact questions; Table 2), without significant differences among education categories (Table 3). The categories were pooled into the two different groups: under-graduate (Compulsory School, High School and Bachelor Degree) and post-graduate (Master Degree and Doctorate of Philosophy). Both under-graduate and post-graduate showed the mean score of the post-questionnaire significantly higher than that of the pre-questionnaire for the overall questionnaire, the reef biology and the human impact questions (Table 2). Considering the overall questionnaire, the mean score of the pre-questionnaire was significantly higher in post-graduate than in under-graduate volunteers (Table 3). However, the mean score of the post-questionnaire and the increase of the mean score between pre- and post-questionnaire didn’t show significant differences between under-graduates and post-graduates (Table 3). Considering the reef biology and the human impact questions, the mean score of the pre-questionnaire, the mean score of the post-questionnaire and the increase of the mean score between pre- and post-questionnaire didn’t show significant differences between under-graduates and post-graduates (Table 3).

According to the diving experience, all categories showed the mean score of the post-questionnaire significantly higher than that of the pre-questionnaire for the overall questionnaire, the reef biology and the human impact questions (except for the category “*Rescue*” for the mean score of the reef biology and the human impact questions and for the category “*Instructor*” for the mean score of the human impact questions; Table 2). Considering the overall questionnaire, the mean score of the post-questionnaire showed significant difference among the categories, the post-hoc tests showed significant difference between the category *Snorkelers* and the categories *Open Water Divers and Instructors* (*p* = 0.008; 0045; Table 3). The mean score of the pre-questionnaire and the increase of the mean score between pre- and post-questionnaire didn’t show significant differences among diving experience categories (Table 3). Considering the reef biology questions, the mean score of the pre-questionnaire, the mean score of the post-questionnaire and the increase of the mean score between pre- and post-questionnaire didn’t show significant differences among the categories (Table 3). Considering the human impact questions, the mean score of the pre-questionnaire and the increase of the mean score between pre- and post-questionnaire showed significant differences among the categories. For the mean score of the pre-questionnaire, the post-hoc tests showed a significant difference between the category *Open Water Divers* and the category *Instructors* (Table 3) and between the category *Divemasters* and the categories *Snorkelers*, *Advanced Open Water Divers*, *Rescue Divers and Instructors* (Table 3). For the increase of the mean score between pre- and post-questionnaire, the post-hoc tests showed a significant difference between the category *Advanced Open Water Divers* and the category *Instructors* (Table 3) and between the category *Divemasters* and *Snorkelers*, *Open Water Divers*, *Advanced Open Water Divers*, *Rescue Divers and Instructors* (Table 3). The mean score of the post-questionnaire didn’t show significant differences among the categories (Table 3). The categories were pooled into two different groups: snorkelers and divers. Both snorkelers and divers showed the mean score of the post-questionnaire significantly higher than that of the pre-questionnaire for the overall questionnaire, the reef biology and human impact questions (Table 2). Considering the overall questionnaire the mean score of the post-questionnaire was significantly higher in divers than in snorkelers (Table 3). The mean score of the pre-questionnaire and the increase of the mean score between pre- and post-questionnaire didn’t show significant differences between the groups (Table 3). Considering the reef biology and the human impact questions, the mean score of the pre-questionnaire, the mean score of the post-questionnaire and the increase of the mean score between pre- and post-questionnaire didn’t show significant differences between the groups (Table 3).

Significant differences between the score of the reef biology questions and that of the human impact questions were detected. All categories and pooled groups (i.e. under-graduate, post-graduate, snorkelers and divers) showed that the mean score of the reef biology questions was significantly lower than that of the human impact questions, both in pre-and post-questionnaire (with the exception of the score of the pre-questionnaire in the category “*Divemaster*” for certification level, and in the post-questionnaire in the category “*Doctor of Philosophy*” see Table 4).

**Table 4:** Results of T student test between the mean score of the reef biology and the human impact questions, in the pre- and in the post-questionnaire.

			*Pre-questionnaire*		*Post-questionnaire*	
		*df*	T	*p*	T	*p*
*Gender*	Female	166	-12.929	< 0.001	-8.737	< 0.001
	Male	254	-17.993	< 0.001	-12.714	< 0.001
*Age*	< 15 years old	18	-6.508	< 0.001	-4.256	< 0.001
	16–30 years old	86	-12.208	< 0.001	-6.275	< 0.001
	31–45 years old	166	-14.107	< 0.001	-8.792	< 0.001
	46–60 years old	130	-10.493	< 0.001	-9.707	< 0.001
	> 61 years old	14	-3.111	0.008	-3.874	0.002
*Level of education*	Compulsory School	82	-9.681	< 0.001	-7.946	< 0.001
	High School	186	-15.300	< 0.001	-10.979	< 0.001
	Bachelor Degree	52	-5.995	< 0.001	-3.767	< 0.001
	Master Degree	90	-11.174	< 0.001	-6.657	< 0.001
	Doctorate of Philosophy	4	-4.285	0.013	**-2.115**	**0.102**
	Under-graduate	324	-18.734	< 0.001	-13621	< 0.001
	Post-graduate	96	-11.851	< 0.001	-7.037	< 0.001
*Diving qualification*	None	270	-18.490	< 0.001	-12.288	< 0.001
	Open Water Diver	38	-6.671	< 0.001	-2.877	0.007
	Advanced Open Water Diver	60	-8.456	< 0.001	-7.746	< 0.001
	Rescue Diver	14	-3.828	0.002	-3.010	0.009
	Divemaster	8	**-1.040**	**0.329**	-2.732	0.026
	Instructor	22	-6.177	< 0.001	-3.711	0.001
	Non-diver	270	-18.490	< 0.001	-12.288	< 0.001
	Diver	150	-12.122	< 0.001	-9.160	< 0.001

In the table are represented the value of the T-student Test (*T*) and the level of significance (*p*). The non-significant differences of the T-student test are in bold.

## Discussion

We found that the participation in a citizen-science monitoring project significantly increased both the knowledge of coral reef biology and ecology and the awareness of human behavioural impacts on the environment. The overall number of correct answers after participation in the project was 25.6% higher than before. According to the reef biology knowledge and the human impact awareness questions, the increase was respectively 36.5% and 12.2%. Our results showed that the level of environmental education of tourists who reach the Red Sea is quite low, (only 32.1% scored more than 7 in the pre-questionnaire, but 86.8% scored more than 7 in the post-questionnaire). From an environmental conservation perspective, this means that tourists represent a serious potential threat for coral reefs, as several previous studies have shown [26, 33–36]. Environmental education is important because it can be determinant of more specific attitudes that, in turn, can help to change human intentions and behaviour toward natural resources such as coral reefs [37; 38]. If people know about organism ecological features or how their own behaviour impacts the reefs, they may be more concerned about the health of the natural resources and also more careful to avoid erroneous behaviours such as touching or interfering with coral reef species.

The analyses to detect differences between categories showed that tourists with a higher education level have a higher initial environmental knowledge and awareness than less educated people, which is in line with normal expectations. The higher mean score of the post-questionnaire for divers compared to that of snorkelers is remarkable, which seems to indicate that the project was more effective on divers than snorkelers. Two motivations could explain this result. The first could be the higher interest and motivation of divers to protect the marine environment. Previous studies have shown that the biocentric orientation of divers is related to the degree of learning and to the fact that divers are well-disposed towards environmental education programs [39; 40]. Future citizen science projects aiming to influence volunteers’ environmental education should focus on this aspect during the design process, to tackle the different citizens’ motivation to participate and their value orientations. A complementary explanation for the higher mean score of the post-questionnaire for divers compared to that of snorkelers is related to the long-term effectiveness of environmental education projects. Divers could have acquired knowledge similar to that provided by the project during their diving training and have lost it before the participation in the project. In this case, the project just reminded them issues they already knew about. This aspect is also discussed in the following “*Limitation*” paragraph.

Another consideration could be made by taking into account the score of the reef biology questions and that for the human impact questions. All categories and pooled groups showed a significantly lower mean score of the reef biology questions than that of the human impact questions (with the exception of the category of “*Divemaster*” and “*Doctor of Philosophy*”, that could also be an artefact, given the very low number of volunteer in this category, respectively N = 5 and N = 3). This could mean that volunteers know that specific behaviours are wrong, but they don’t know exactly how these behaviours affect the environment and the organisms. This result confirms previous findings. Barker and Roberts [21] have shown that if the briefing is short and given by local staff it does not reduce diver contact rate with the reef or the probability of a diver breaking living substrate. Camp and Fraser [41] found that only more detailed briefings (that included legal requirements of the area, scientific evidences and generational equity) significantly reduced the number of diver interactions with the substrate. Several studies have shown that briefings decreased the diving impact on the natural environments but several other studies have shown that divers continue to have an impact. These findings seem to show that very short briefings, that probably represent the more realistic commitment for a dive company with time-wise and other constraints, is not enough to affect the diver behaviour. To use briefings as effective education programs they should be more detailed and last longer than what is normally proposed by dive leaders.

### Limitations

First of all, we must consider that people voluntarily decided to participate in the project. This could mean that involved volunteers were potentially more likely to learn about environmental issues and this could affect the results of this study, preventing a generalization to the broad public of the very promising results obtained here.

The present study didn’t evaluate the long-term effectiveness of the participation in the monitoring program, since the post-questionnaire was filled in during the last day of the volunteers’ holiday. Unfortunately, none of the surveyed volunteers had already participated in the project in the previous years. Further studies should be necessary to examine if the acquired knowledge and awareness remain several months after the participation in the project and if citizen science programs prompt long-term environmentally responsible attitudes and behaviour in participants. Further studies could also explain the better performance of divers than snorkelers, in terms of a long-term effectiveness of environmental education projects. Further studies should also take into account the different role of coral reef biology and ecology knowledge and human behaviour impact awareness. Understanding how behaviour affects the organisms and the environments they live in could play a key role in determining a change in the attitude and behaviour of people towards the environment.

## Conclusion

As emphasized in this study, citizen science projects have an important and effective educational value. Thanks to the recreational approach, STE project has engage a relevant number of volunteers and increased the environmental education of the participants of all ages, gender, education level or diving experience. The results of this study have also suggested that tourism and diving stakeholders should increase their commitment and efforts to these programs for different reasons.

First of all, more educated and, consequentially, more sustainable tourists are of central interest for stakeholders to preserve the environment that primarily supports their business. In addition, the environmental education of tourists, which leads to a decrease in the frequency of environmental impacting activities, raises the carrying capacity of the environment [19], boosting the economical business.

Barker and Roberts [21] have argued that, often, diving companies are unable to provide a briefing that guarantees a sufficient number of environmental education information. Implementing citizen science programs could enhance the possibility for the dive leaders to create moments to talk about the environment and how to approach it or provide scientific figures (research volunteers, students) to assure these educational activities are carried out.

Third, as suggested by Orams and Hill [23], citizen science and educational programs could represent a marketing tool, which increases the acceptance of tourism involving a sustainable exploitation of the environment, fostering a green reputation for the company.

## Supporting Information

S1 Table Dataset. (HTM)
