# Supplementary material for: Participating in a Citizen Science Monitoring Program: Implications for Environmental Education
Source: PLoS One. 2015 Jul 22;10(7):e0131812. doi: 10.1371/journal.pone.0131812 (PMC4511791; doi:10.1371/journal.pone.0131812)
Supplement: S1 Table — (HTM) [file pone.0131812.s001.htm]

|  | Demographic data | | | | Pre questionnaire | | | | | | | | | | | | | | | | | | | | | | | | | | | | | | | | | | | | | | | | | | | | | | | | | | | | | | | | | | | | | | | | | | | | | Post questionnaire | | | | | | | | | | | | | | | | | | | | | | | | | | | | | | | | | | | | | | | | | | | | | | | | | | | | | | | | | | | | | | | | | | | | |
| ID | GEN | AGE | EDU | EXP | 1a | 1b | 1c | 2a | 2b | 2c | 2d | 2e | 3a | 3b | 3c | 3d | 3e | 4a | 4b | 4c | 4d | 5a | 5b | 5c | 5d | 6a | 6b | 6c | 6d | 7a | 7b | 7c | 7d | 7e | 7f | 7g | 7h | 8a | 8b | 8c | 8d | 8e | 8f | 9a | 9b | 9c | 9d | 10a | 10b | 10c | 10d | 10e | 11a | 11b | 11c | 11d | 11e | 12a | 12b | 12c | 13a | 13b | 13c | 13d | 13e | 14a | 14b | 14c | 14d | 14e | 15a | 15b | 15c | 1a | 1b | 1c | 2a | 2b | 2c | 2d | 2e | 3a | 3b | 3c | 3d | 3e | 4a | 4b | 4c | 4d | 5a | 5b | 5c | 5d | 6a | 6b | 6c | 6d | 7a | 7b | 7c | 7d | 7e | 7f | 7g | 7h | 8a | 8b | 8c | 8d | 8e | 8f | 9a | 9b | 9c | 9d | 10a | 10b | 10c | 10d | 10e | 11a | 11b | 11c | 11d | 11e | 12a | 12b | 12c | 13a | 13b | 13c | 13d | 13e | 14a | 14b | 14c | 14d | 14e | 15a | 15b | 15c |
| 1 | 1 | 4 | 2 | 1 |  | 1 |  |  | 1 |  |  |  |  |  |  | 1 |  | 1 |  |  |  |  |  | 1 |  |  |  |  | 1 | 1 | 1 |  |  | 1 | 1 |  |  |  |  |  |  | 1 |  | 1 |  |  |  |  | 1 |  | 1 |  |  |  |  | 1 |  |  | 1 |  |  | 1 |  |  |  |  | 1 |  |  |  | 1 |  |  |  | 1 |  |  | 1 |  |  |  | 1 |  |  |  |  | 1 |  |  |  |  | 1 |  |  |  |  |  | 1 | 1 | 1 |  |  | 1 | 1 |  |  |  |  |  |  | 1 |  |  |  | 1 |  |  | 1 | 1 | 1 |  |  |  | 1 |  |  | 1 |  |  |  | 1 |  |  |  |  | 1 |  |  |  | 1 |  |  |
| 2 | 1 | 3 | 1 | 1 |  | 1 |  |  | 1 |  |  |  |  |  |  | 1 |  | 1 |  |  |  |  |  | 1 |  |  |  |  | 1 |  | 1 | 1 |  | 1 | 1 |  |  |  |  |  |  | 1 |  |  |  |  | 1 |  | 1 | 1 | 1 |  |  |  |  | 1 |  |  |  | 1 |  | 1 |  |  |  |  | 1 | 1 |  |  | 1 |  |  |  | 1 |  |  | 1 |  |  |  | 1 |  |  | 1 |  | 1 |  |  |  |  |  | 1 |  | 1 |  |  |  | 1 | 1 | 1 |  | 1 | 1 |  |  |  |  |  |  | 1 |  |  |  |  | 1 |  | 1 | 1 | 1 |  |  |  |  | 1 |  | 1 |  |  |  | 1 |  | 1 |  |  | 1 | 1 |  |  | 1 |  |  |
| 3 | 1 | 3 | 4 | 1 |  | 1 |  | 1 |  |  |  |  | 1 |  |  |  |  |  | 1 |  |  |  | 1 |  |  |  |  |  | 1 | 1 |  |  |  | 1 |  | 1 |  |  |  |  | 1 |  |  |  |  |  | 1 |  | 1 |  | 1 |  |  |  |  |  | 1 |  |  | 1 |  | 1 |  | 1 |  |  |  | 1 |  |  | 1 |  |  |  | 1 |  | 1 |  |  |  |  |  |  |  |  | 1 | 1 |  |  |  |  | 1 |  |  |  |  |  | 1 | 1 |  |  |  | 1 | 1 |  |  |  |  |  | 1 |  |  |  | 1 |  |  |  | 1 |  | 1 |  |  |  |  | 1 |  | 1 |  |  |  | 1 |  | 1 |  | 1 |  | 1 |  |  | 1 |  |  |
| 4 | 1 | 4 | 2 | 3 | 1 |  |  |  |  |  |  | 1 |  |  |  |  | 1 |  | 1 |  |  |  | 1 |  |  |  |  |  | 1 | 1 |  |  |  | 1 | 1 |  |  |  |  |  |  | 1 |  |  |  |  | 1 |  | 1 |  | 1 |  |  |  | 1 |  |  |  | 1 |  |  | 1 |  | 1 |  | 1 |  |  | 1 |  | 1 |  |  | 1 |  |  |  | 1 |  |  |  | 1 |  |  |  |  |  | 1 |  |  |  | 1 |  |  |  | 1 |  |  | 1 | 1 |  |  | 1 |  |  |  |  |  |  | 1 |  |  | 1 |  |  |  |  | 1 | 1 | 1 |  |  |  | 1 |  |  | 1 |  |  |  | 1 |  | 1 |  |  |  | 1 |  |  | 1 |  |  |
| 5 | 1 | 5 | 4 | 1 |  | 1 |  |  | 1 |  |  |  |  |  |  | 1 |  | 1 |  |  |  |  | 1 |  |  |  |  |  | 1 |  | 1 |  |  |  | 1 |  |  |  |  |  | 1 |  |  | 1 |  |  |  |  | 1 |  |  |  |  |  |  |  | 1 |  |  | 1 |  | 1 |  |  |  |  |  | 1 |  |  | 1 |  |  |  | 1 |  |  | 1 |  |  |  | 1 |  |  |  |  | 1 |  |  |  |  | 1 |  |  |  |  |  | 1 | 1 | 1 |  |  | 1 | 1 |  |  |  |  |  |  | 1 |  |  |  |  | 1 | 1 | 1 | 1 | 1 |  |  |  | 1 |  |  | 1 |  |  |  | 1 |  |  |  |  | 1 | 1 |  |  | 1 |  |  |
| 6 | 1 | 4 | 3 | 1 |  | 1 |  |  | 1 |  |  |  |  |  |  | 1 |  | 1 |  |  |  |  | 1 |  |  |  |  | 1 |  |  |  |  |  |  | 1 |  |  |  |  |  |  | 1 |  | 1 |  |  |  |  |  |  |  | 1 |  |  |  | 1 |  | 1 |  |  |  | 1 |  | 1 |  |  | 1 |  |  |  |  | 1 |  |  | 1 |  |  | 1 |  |  |  |  |  |  | 1 |  | 1 |  |  |  |  | 1 |  |  |  |  | 1 |  | 1 |  |  |  |  |  | 1 |  |  |  |  |  | 1 |  |  |  | 1 |  | 1 | 1 | 1 | 1 |  |  |  | 1 |  |  |  | 1 |  |  | 1 |  | 1 |  |  | 1 | 1 |  |  | 1 |  |  |
| 7 | 1 | 3 | 2 | 4 | 1 |  |  |  | 1 |  |  |  | 1 |  |  |  |  | 1 |  |  |  |  | 1 |  |  |  |  |  | 1 | 1 | 1 |  |  |  | 1 |  |  |  |  |  | 1 |  |  |  |  |  | 1 |  |  |  | 1 |  |  |  |  |  | 1 |  | 1 |  |  |  |  | 1 |  |  |  |  |  | 1 | 1 |  |  | 1 |  |  |  | 1 |  |  |  |  |  |  |  | 1 | 1 |  |  |  |  | 1 |  |  |  |  |  | 1 |  | 1 |  |  |  | 1 |  |  |  | 1 |  |  |  |  |  | 1 |  |  |  |  |  | 1 |  |  |  | 1 |  |  | 1 |  |  |  | 1 |  |  |  |  |  | 1 |  |  | 1 |  |  |
| 8 | 2 | 3 | 3 | 1 |  | 1 |  |  | 1 |  |  |  |  |  |  | 1 |  | 1 |  |  |  |  | 1 |  |  |  |  | 1 |  | 1 | 1 |  |  | 1 | 1 |  |  |  |  |  | 1 |  |  | 1 |  |  |  | 1 | 1 |  | 1 |  |  |  | 1 |  |  | 1 |  |  |  | 1 |  | 1 |  |  | 1 | 1 |  |  | 1 |  |  |  | 1 |  |  | 1 |  |  |  | 1 |  |  |  |  | 1 |  |  |  |  | 1 |  |  |  |  | 1 |  | 1 | 1 |  |  | 1 | 1 | 1 |  |  |  |  | 1 |  |  | 1 |  |  |  |  | 1 | 1 | 1 |  |  |  | 1 |  |  | 1 |  |  |  | 1 |  | 1 |  |  | 1 | 1 |  |  | 1 |  |  |
| 9 | 2 | 3 | 2 | 3 |  | 1 |  |  | 1 |  |  |  |  |  |  | 1 |  | 1 |  |  |  |  | 1 |  |  |  | 1 |  |  | 1 | 1 |  |  | 1 | 1 |  |  |  |  |  | 1 |  |  | 1 |  |  |  | 1 | 1 | 1 | 1 |  |  |  |  | 1 |  | 1 |  |  |  | 1 |  | 1 |  |  |  | 1 |  |  | 1 |  |  |  | 1 |  |  | 1 |  |  |  |  |  |  | 1 |  | 1 |  |  |  |  | 1 |  |  |  |  | 1 |  | 1 | 1 |  |  | 1 | 1 |  |  |  |  |  | 1 |  |  | 1 |  |  |  | 1 | 1 | 1 | 1 |  |  |  |  | 1 |  | 1 |  |  |  | 1 |  | 1 |  |  | 1 | 1 |  |  | 1 |  |  |
| 10 | 2 | 4 | 4 | 1 |  | 1 |  | 1 |  |  |  |  |  |  |  |  | 1 | 1 |  |  |  |  | 1 |  |  |  |  |  | 1 | 1 | 1 |  |  | 1 | 1 |  |  |  |  |  | 1 |  |  |  |  |  | 1 |  | 1 |  | 1 |  |  |  | 1 |  |  | 1 |  |  |  | 1 |  | 1 |  |  | 1 | 1 |  |  | 1 |  |  |  | 1 |  |  | 1 |  |  |  | 1 |  |  |  |  | 1 |  |  |  |  | 1 |  |  |  |  |  | 1 | 1 | 1 |  |  | 1 | 1 |  |  |  |  |  | 1 |  |  | 1 |  |  |  |  | 1 |  | 1 |  |  |  | 1 |  |  | 1 |  |  |  | 1 |  | 1 |  |  | 1 | 1 |  |  | 1 |  |  |
| 11 | 1 | 2 | 1 | 1 | 1 |  |  | 1 |  |  |  |  |  |  |  |  | 1 |  |  |  | 1 |  |  | 1 |  |  |  |  | 1 |  |  |  |  | 1 | 1 |  |  |  |  |  |  | 1 |  |  |  |  | 1 |  |  |  | 1 |  |  |  | 1 |  |  | 1 |  |  |  |  |  | 1 |  |  | 1 | 1 |  |  | 1 |  |  |  | 1 |  |  | 1 |  |  |  | 1 |  |  |  |  | 1 |  |  |  |  | 1 |  |  |  |  |  | 1 | 1 | 1 |  |  | 1 | 1 |  |  |  |  |  |  | 1 |  | 1 |  |  |  |  | 1 |  | 1 |  |  |  | 1 |  |  | 1 |  |  |  | 1 |  | 1 |  |  | 1 |  |  |  | 1 |  |  |
| 12 | 1 | 2 | 1 | 1 |  | 1 |  |  | 1 |  |  |  |  |  |  | 1 | 1 |  |  |  | 1 |  |  | 1 |  |  |  |  | 1 |  | 1 |  |  | 1 | 1 | 1 |  |  |  |  | 1 |  |  | 1 |  |  |  |  | 1 |  | 1 |  |  |  |  | 1 |  | 1 |  |  |  | 1 |  | 1 |  |  | 1 | 1 |  |  | 1 |  |  |  | 1 |  |  | 1 |  |  |  |  |  |  | 1 |  |  |  |  | 1 |  |  |  | 1 |  |  |  | 1 |  | 1 |  |  | 1 | 1 | 1 |  |  |  |  | 1 |  |  | 1 |  |  |  |  | 1 | 1 | 1 |  |  |  |  | 1 |  | 1 |  |  |  | 1 |  | 1 |  |  | 1 | 1 |  |  | 1 |  |  |
| 13 | 1 | 2 | 2 | 3 |  |  | 1 | 1 |  |  |  |  |  |  |  | 1 |  | 1 |  |  |  |  |  | 1 |  |  |  |  | 1 |  |  | 1 |  | 1 | 1 |  |  |  |  |  | 1 |  |  |  |  |  | 1 | 1 | 1 |  | 1 |  |  |  | 1 |  |  | 1 |  |  |  | 1 |  | 1 |  |  | 1 |  |  |  | 1 |  |  | 1 |  |  | 1 |  |  |  |  | 1 |  |  | 1 |  | 1 |  |  |  |  |  | 1 |  |  |  |  | 1 | 1 | 1 |  |  | 1 | 1 |  |  |  |  |  | 1 |  |  |  |  |  | 1 | 1 | 1 |  | 1 |  |  |  | 1 |  |  | 1 |  |  |  | 1 |  | 1 |  |  | 1 |  | 1 |  |  |  | 1 |
| 14 | 2 | 3 | 4 | 1 |  | 1 |  |  | 1 |  |  |  |  |  |  | 1 |  |  |  | 1 |  |  | 1 |  |  |  |  |  | 1 | 1 | 1 |  |  | 1 | 1 | 1 |  |  |  |  |  | 1 |  | 1 |  |  |  |  | 1 |  | 1 |  |  |  |  | 1 |  |  | 1 |  |  | 1 |  | 1 |  |  | 1 | 1 |  |  | 1 |  |  |  | 1 |  |  | 1 |  |  |  |  |  |  | 1 |  |  |  | 1 |  |  |  |  | 1 |  |  |  | 1 | 1 | 1 |  |  | 1 | 1 | 1 |  |  |  |  | 1 |  |  | 1 |  |  |  | 1 | 1 | 1 | 1 |  |  |  | 1 |  |  | 1 |  |  |  | 1 |  | 1 |  |  | 1 | 1 |  |  | 1 |  |  |
| 15 | 2 | 3 | 2 | 1 |  | 1 |  |  | 1 |  |  |  | 1 |  |  |  |  | 1 |  |  |  |  | 1 |  |  |  | 1 |  |  | 1 | 1 |  |  | 1 | 1 |  |  |  |  |  |  |  | 1 |  |  |  | 1 | 1 | 1 | 1 | 1 |  |  |  |  | 1 |  | 1 |  |  |  | 1 |  | 1 |  |  |  | 1 |  |  | 1 |  |  | 1 |  |  |  | 1 |  |  |  | 1 |  |  |  |  | 1 |  |  |  |  | 1 |  |  |  | 1 |  |  |  | 1 |  |  | 1 | 1 |  |  |  |  |  | 1 |  |  |  |  | 1 |  | 1 | 1 | 1 | 1 |  |  |  |  | 1 |  | 1 |  |  |  | 1 |  | 1 |  |  |  | 1 |  |  | 1 |  |  |
| 16 | 2 | 4 | 1 | 1 |  |  | 1 |  |  |  |  | 1 |  |  |  |  | 1 | 1 |  |  |  | 1 |  |  |  |  |  |  | 1 |  |  |  |  | 1 | 1 |  |  |  |  |  |  |  | 1 |  |  |  | 1 |  |  |  | 1 |  |  |  |  |  | 1 |  | 1 |  |  |  |  |  | 1 |  |  | 1 |  |  |  |  | 1 |  | 1 |  |  |  |  |  | 1 |  |  |  |  | 1 | 1 |  |  |  |  |  |  | 1 |  |  |  | 1 |  |  |  |  | 1 | 1 |  |  |  |  |  | 1 |  |  |  |  |  | 1 |  |  |  | 1 |  |  |  |  |  | 1 |  |  | 1 |  |  |  |  | 1 |  |  | 1 |  |  | 1 |  |  |
| 17 | 1 | 2 | 2 | 2 | 1 |  |  | 1 |  |  |  |  |  |  |  |  | 1 |  | 1 |  |  |  | 1 |  |  |  |  |  | 1 |  |  |  |  | 1 | 1 |  |  |  |  |  |  |  | 1 | 1 |  |  |  |  | 1 |  | 1 |  |  |  |  | 1 |  | 1 |  |  |  | 1 |  | 1 |  |  |  |  |  | 1 | 1 |  |  |  | 1 |  |  | 1 |  |  |  | 1 |  |  |  |  | 1 |  |  |  |  | 1 |  |  |  |  |  | 1 |  | 1 |  |  | 1 | 1 |  |  |  |  |  | 1 |  |  | 1 |  |  |  |  | 1 |  | 1 |  |  |  |  | 1 |  | 1 |  |  |  | 1 |  | 1 |  |  |  | 1 |  |  | 1 |  |  |
| 18 | 2 | 3 | 3 | 1 |  | 1 |  |  |  |  | 1 |  |  |  |  | 1 |  |  | 1 |  |  |  |  | 1 |  |  |  |  | 1 | 1 | 1 |  |  | 1 | 1 | 1 |  |  |  |  |  | 1 |  |  |  |  | 1 |  | 1 | 1 | 1 |  |  |  | 1 | 1 |  | 1 |  |  |  | 1 |  |  |  |  | 1 |  |  |  | 1 |  |  |  | 1 |  |  | 1 |  |  |  | 1 |  |  |  |  | 1 |  |  |  |  |  | 1 |  |  |  |  | 1 | 1 | 1 |  |  | 1 | 1 | 1 |  |  |  |  |  | 1 |  | 1 |  |  |  |  | 1 | 1 | 1 |  |  |  |  | 1 |  | 1 |  |  |  | 1 |  |  |  |  |  | 1 |  |  | 1 |  |  |
| 19 | 2 | 2 | 4 | 1 |  | 1 |  |  | 1 |  |  |  | 1 |  |  |  |  |  | 1 |  |  |  | 1 |  |  |  |  | 1 |  | 1 | 1 |  |  | 1 | 1 |  |  |  |  |  |  | 1 |  | 1 |  |  |  | 1 | 1 | 1 | 1 |  |  |  |  | 1 |  | 1 |  |  |  | 1 |  | 1 |  |  |  | 1 |  |  | 1 |  |  |  | 1 |  |  | 1 |  |  |  | 1 |  |  |  |  |  | 1 |  |  |  | 1 |  |  |  |  | 1 |  | 1 | 1 |  | 1 |  | 1 |  |  |  |  |  |  | 1 |  | 1 |  |  |  |  | 1 | 1 | 1 |  |  |  |  | 1 |  | 1 |  |  |  | 1 |  | 1 |  |  |  | 1 |  |  | 1 |  |  |
| 20 | 2 | 2 | 3 | 1 | 1 |  |  |  |  | 1 |  |  |  |  |  | 1 |  | 1 |  |  |  |  |  | 1 |  |  |  |  | 1 |  | 1 |  |  | 1 | 1 | 1 |  |  |  |  |  | 1 |  |  |  |  | 1 |  | 1 |  | 1 |  |  |  |  | 1 |  | 1 |  |  |  | 1 |  |  |  |  | 1 |  |  |  | 1 |  |  |  | 1 |  |  | 1 |  |  |  |  |  |  | 1 |  |  | 1 |  |  |  |  | 1 |  |  |  |  | 1 | 1 | 1 |  |  |  | 1 |  |  |  |  |  |  | 1 |  | 1 |  |  |  |  | 1 | 1 | 1 |  |  |  |  | 1 |  | 1 |  |  |  | 1 |  | 1 |  |  |  | 1 |  |  | 1 |  |  |
| 21 | 2 | 2 | 2 | 4 | 1 |  |  |  | 1 |  |  |  |  |  |  |  | 1 | 1 |  |  |  |  | 1 |  |  |  | 1 |  |  |  |  |  |  |  | 1 |  |  |  |  |  | 1 |  |  |  |  |  | 1 | 1 | 1 |  | 1 |  |  |  | 1 |  |  | 1 |  |  |  | 1 |  | 1 |  |  |  | 1 |  |  | 1 |  |  |  | 1 |  |  | 1 |  |  |  | 1 |  |  |  |  | 1 |  |  |  |  | 1 |  |  |  | 1 |  |  | 1 | 1 |  |  | 1 | 1 |  |  |  |  |  | 1 |  |  |  |  | 1 |  | 1 | 1 |  | 1 |  |  |  | 1 |  |  |  |  | 1 |  | 1 |  | 1 |  |  |  | 1 |  |  | 1 |  |  |
| 22 | 1 | 3 | 2 | 6 |  | 1 |  |  | 1 |  |  |  | 1 |  |  |  |  | 1 |  |  |  |  | 1 |  |  |  |  |  | 1 |  |  |  |  | 1 | 1 |  |  |  |  |  |  |  | 1 |  |  |  | 1 |  | 1 |  | 1 |  |  |  |  | 1 |  |  |  | 1 |  | 1 |  | 1 |  |  |  | 1 |  |  | 1 |  |  |  | 1 |  |  | 1 |  |  |  | 1 |  |  |  |  | 1 |  |  |  |  | 1 |  |  |  |  |  | 1 | 1 | 1 |  |  | 1 |  | 1 |  |  |  |  |  |  | 1 |  |  |  | 1 |  | 1 | 1 | 1 |  |  |  |  | 1 |  | 1 |  |  |  | 1 |  | 1 |  |  |  | 1 |  |  | 1 |  |  |
| 23 | 1 | 4 | 2 | 1 | 1 |  |  | 1 |  |  |  |  |  |  |  |  | 1 | 1 |  |  |  |  | 1 |  |  |  |  |  | 1 |  |  | 1 |  | 1 |  |  |  |  |  |  |  | 1 |  |  | 1 |  |  | 1 | 1 |  |  |  |  |  | 1 |  |  | 1 |  |  |  | 1 |  | 1 |  |  | 1 | 1 |  |  | 1 |  |  |  | 1 |  |  | 1 |  |  |  | 1 |  |  |  |  | 1 |  |  |  |  | 1 |  |  |  | 1 |  |  | 1 | 1 |  |  | 1 | 1 |  |  |  |  |  |  | 1 |  | 1 |  |  |  |  | 1 |  | 1 |  |  |  | 1 |  |  | 1 |  |  |  | 1 |  | 1 |  |  | 1 | 1 |  |  | 1 |  |  |
| 24 | 2 | 2 | 1 | 1 |  | 1 |  |  | 1 |  |  |  |  |  |  | 1 |  | 1 |  |  |  |  | 1 |  |  |  |  |  | 1 |  | 1 |  |  |  |  |  |  |  |  |  |  | 1 |  | 1 |  |  |  |  | 1 |  | 1 |  |  |  | 1 |  |  |  |  | 1 |  | 1 |  | 1 |  |  | 1 |  |  |  |  |  | 1 |  | 1 |  |  | 1 |  |  |  | 1 |  |  |  |  | 1 |  |  |  |  | 1 |  |  |  | 1 |  |  | 1 | 1 |  |  | 1 | 1 |  |  |  |  |  |  | 1 |  | 1 |  |  |  |  | 1 |  | 1 |  |  |  | 1 |  |  | 1 |  |  |  | 1 |  | 1 |  |  | 1 | 1 |  |  | 1 |  |  |
| 25 | 1 | 3 | 4 | 5 |  | 1 |  |  | 1 |  |  |  |  |  |  | 1 |  | 1 |  |  |  |  | 1 |  |  |  |  |  | 1 | 1 |  | 1 |  | 1 | 1 | 1 |  |  |  |  |  | 1 |  | 1 |  |  |  |  | 1 |  | 1 |  |  |  | 1 |  |  | 1 |  |  |  | 1 |  | 1 |  |  | 1 | 1 |  |  | 1 |  |  |  | 1 |  |  | 1 |  |  |  | 1 |  |  |  |  | 1 |  |  |  |  | 1 |  |  |  | 1 |  |  | 1 | 1 |  |  | 1 | 1 |  |  |  |  |  |  | 1 |  | 1 |  |  |  |  | 1 |  | 1 |  |  |  | 1 |  |  | 1 |  |  |  | 1 |  | 1 |  |  | 1 | 1 |  |  | 1 |  |  |
| 26 | 1 | 3 | 2 | 1 |  | 1 |  |  | 1 |  |  |  |  |  |  | 1 |  |  | 1 |  |  | 1 |  |  |  |  |  |  | 1 |  | 1 |  |  |  |  |  |  |  |  |  |  | 1 |  | 1 |  |  |  |  |  |  | 1 |  |  |  | 1 |  |  | 1 |  |  |  | 1 |  | 1 |  |  | 1 | 1 |  |  | 1 |  |  |  | 1 |  |  | 1 |  |  |  |  |  |  | 1 |  | 1 |  |  |  |  | 1 |  |  |  |  |  | 1 |  |  |  |  | 1 | 1 | 1 |  |  |  |  |  | 1 |  |  | 1 |  |  |  | 1 |  | 1 |  |  |  | 1 |  |  | 1 |  |  |  | 1 |  | 1 |  |  | 1 | 1 |  |  | 1 |  |  |
| 27 | 1 | 3 | 1 | 1 |  | 1 |  |  | 1 |  |  |  |  |  |  | 1 |  | 1 |  |  |  |  |  | 1 |  |  | 1 |  |  |  |  |  |  | 1 | 1 |  |  |  |  |  |  | 1 |  | 1 |  |  |  |  | 1 |  | 1 |  |  |  | 1 |  |  | 1 |  |  |  | 1 |  | 1 |  |  | 1 | 1 |  |  | 1 |  |  |  | 1 |  |  | 1 |  |  |  | 1 |  |  |  |  | 1 |  |  |  |  | 1 |  |  |  | 1 |  |  | 1 |  |  |  | 1 | 1 |  |  |  |  |  |  |  | 1 |  |  | 1 |  |  | 1 |  | 1 |  |  |  | 1 |  |  | 1 |  |  |  | 1 |  | 1 |  |  | 1 | 1 |  |  | 1 |  |  |
| 28 | 1 | 2 | 1 | 1 |  | 1 |  |  | 1 |  |  |  |  |  |  | 1 |  | 1 |  |  |  |  |  | 1 |  |  | 1 |  |  |  |  |  |  | 1 | 1 |  |  |  |  |  |  | 1 |  | 1 |  |  |  |  | 1 |  | 1 |  |  |  | 1 |  |  | 1 |  |  |  | 1 |  | 1 |  |  | 1 | 1 |  |  | 1 |  |  |  | 1 |  |  | 1 |  |  |  | 1 |  |  |  |  | 1 |  |  |  |  | 1 |  |  |  | 1 |  |  | 1 |  |  |  | 1 | 1 |  |  |  |  |  |  |  | 1 |  |  | 1 |  |  | 1 |  | 1 |  |  |  | 1 |  |  | 1 |  |  |  | 1 |  | 1 |  |  | 1 | 1 |  |  | 1 |  |  |
| 29 | 2 | 3 | 4 | 1 |  | 1 |  |  |  |  |  | 1 |  |  |  | 1 |  |  | 1 |  |  |  |  | 1 |  |  |  |  | 1 |  |  |  |  |  | 1 |  |  |  |  |  |  | 1 |  |  |  |  | 1 |  | 1 |  | 1 |  |  |  | 1 |  |  | 1 |  |  |  | 1 |  | 1 |  |  | 1 |  |  |  | 1 |  |  |  | 1 |  | 1 |  |  |  |  |  |  |  | 1 |  | 1 |  |  |  |  |  | 1 |  |  |  | 1 |  |  |  |  |  | 1 | 1 |  |  |  |  |  |  | 1 |  | 1 |  |  |  |  | 1 |  | 1 |  |  |  | 1 |  |  | 1 |  |  |  | 1 |  | 1 |  |  | 1 |  |  |  | 1 |  |  |
| 30 | 2 | 3 | 2 | 1 |  |  | 1 |  | 1 |  |  |  |  |  |  |  | 1 | 1 |  |  |  |  | 1 |  |  |  |  |  | 1 |  | 1 |  |  | 1 | 1 |  |  |  |  |  |  | 1 |  |  |  |  | 1 |  | 1 |  | 1 |  |  |  | 1 | 1 | 1 | 1 |  |  |  | 1 |  | 1 |  |  | 1 | 1 |  |  | 1 |  |  |  | 1 |  |  | 1 |  |  |  | 1 |  |  |  |  | 1 |  |  |  |  | 1 |  |  |  | 1 |  |  | 1 | 1 |  |  | 1 | 1 |  |  |  |  |  |  | 1 |  | 1 |  |  |  |  | 1 |  | 1 |  |  |  | 1 |  |  | 1 |  |  |  | 1 |  | 1 |  |  | 1 | 1 |  |  | 1 |  |  |
| 31 | 1 | 4 | 2 | 6 |  | 1 |  |  |  |  |  | 1 |  |  |  |  | 1 | 1 |  |  |  |  | 1 |  |  |  |  |  | 1 |  |  |  |  | 1 | 1 |  |  |  |  |  |  |  | 1 |  |  |  | 1 | 1 |  |  | 1 |  |  |  | 1 |  |  |  |  | 1 |  | 1 |  |  |  |  | 1 |  |  |  | 1 |  |  |  | 1 |  |  |  |  |  | 1 |  |  |  |  | 1 | 1 |  |  |  |  | 1 |  |  |  |  |  | 1 |  | 1 |  |  | 1 | 1 |  |  |  |  |  |  |  | 1 |  |  |  | 1 | 1 | 1 |  | 1 |  |  |  | 1 |  |  | 1 |  |  |  | 1 |  | 1 |  |  | 1 |  |  |  | 1 |  |  |
| 32 | 1 | 4 | 2 | 3 |  | 1 |  |  |  |  |  | 1 |  |  |  |  | 1 | 1 |  |  |  |  | 1 |  |  |  |  |  | 1 |  |  |  |  | 1 | 1 |  |  |  |  |  |  |  | 1 |  |  |  | 1 | 1 | 1 |  | 1 |  |  |  | 1 |  |  |  |  | 1 |  | 1 |  |  |  |  | 1 |  |  |  | 1 |  |  |  | 1 |  |  |  |  |  | 1 |  |  |  |  | 1 | 1 |  |  |  |  | 1 |  |  |  |  |  | 1 |  |  |  |  | 1 | 1 |  |  |  |  |  |  |  | 1 |  |  |  | 1 | 1 | 1 |  | 1 |  |  |  | 1 |  |  | 1 |  |  |  | 1 |  | 1 |  |  | 1 |  | 1 |  | 1 |  |  |
| 33 | 1 | 3 | 2 | 1 |  | 1 |  |  | 1 |  |  |  |  |  |  |  | 1 | 1 |  |  |  |  | 1 |  |  |  |  |  | 1 |  | 1 |  |  | 1 | 1 |  |  |  |  |  |  |  | 1 |  |  |  | 1 |  |  |  | 1 |  |  |  |  |  | 1 | 1 |  |  |  | 1 |  | 1 |  |  | 1 | 1 |  |  | 1 |  |  |  | 1 |  |  | 1 |  |  |  | 1 |  |  |  |  | 1 |  |  |  |  | 1 |  |  |  |  |  | 1 | 1 | 1 |  |  | 1 | 1 |  |  |  |  |  | 1 |  |  |  |  | 1 |  |  | 1 |  | 1 |  |  |  | 1 |  |  | 1 |  |  |  | 1 |  | 1 |  |  | 1 |  |  |  | 1 |  |  |
| 34 | 1 | 2 | 2 | 2 |  | 1 |  |  | 1 |  |  |  |  |  |  | 1 |  | 1 |  |  |  |  | 1 |  |  |  |  |  | 1 |  |  |  |  | 1 | 1 |  |  |  |  |  | 1 |  |  |  |  |  | 1 |  | 1 |  | 1 |  |  |  | 1 | 1 |  | 1 |  |  |  | 1 |  | 1 |  |  | 1 |  |  |  | 1 |  |  |  | 1 |  |  | 1 |  |  |  | 1 |  |  |  |  | 1 |  |  |  |  | 1 |  |  |  |  |  | 1 | 1 | 1 |  |  | 1 | 1 |  |  |  |  |  | 1 |  |  |  |  | 1 |  |  | 1 |  | 1 |  |  |  | 1 | 1 |  | 1 |  |  |  | 1 |  | 1 |  |  | 1 |  |  |  | 1 |  |  |
| 35 | 1 | 5 | 2 | 3 |  | 1 |  |  | 1 |  |  |  |  |  |  |  | 1 | 1 |  |  |  |  | 1 |  |  |  |  |  | 1 |  |  |  |  |  |  |  | 1 |  |  |  |  | 1 |  |  |  |  | 1 |  | 1 |  | 1 |  |  |  |  |  | 1 |  |  | 1 |  | 1 |  |  |  |  | 1 |  |  |  | 1 |  |  |  | 1 |  |  | 1 |  |  |  | 1 |  |  |  |  | 1 |  |  |  |  | 1 |  |  |  |  |  | 1 | 1 |  |  |  | 1 | 1 |  |  |  |  |  |  | 1 |  |  |  | 1 |  |  | 1 |  | 1 |  |  |  | 1 |  |  | 1 |  |  |  | 1 |  |  |  |  | 1 |  |  |  | 1 |  |  |
| 36 | 1 | 2 | 1 | 1 | 1 |  |  |  | 1 |  |  |  |  |  |  | 1 |  | 1 |  |  |  |  |  |  | 1 |  | 1 |  |  |  | 1 |  |  | 1 | 1 |  |  |  |  |  | 1 |  |  | 1 |  |  |  | 1 | 1 |  | 1 |  |  |  | 1 |  |  | 1 |  |  |  | 1 |  | 1 |  |  | 1 |  |  |  | 1 |  |  |  | 1 |  |  | 1 |  |  |  | 1 |  |  |  |  | 1 |  |  |  |  | 1 |  |  |  |  | 1 |  |  | 1 |  |  | 1 | 1 |  |  |  |  |  | 1 |  |  |  |  | 1 |  | 1 |  | 1 | 1 |  |  |  | 1 |  |  | 1 |  |  |  | 1 |  | 1 |  |  | 1 |  |  |  | 1 |  |  |
| 37 | 1 | 4 | 1 | 3 |  | 1 |  |  | 1 |  |  |  |  |  |  | 1 |  | 1 |  |  |  |  | 1 |  |  |  |  |  | 1 | 1 |  |  |  | 1 | 1 |  |  |  |  |  | 1 |  |  | 1 |  |  |  |  | 1 |  | 1 |  |  |  | 1 | 1 |  | 1 |  |  |  | 1 |  | 1 |  |  |  |  |  | 1 | 1 |  |  |  | 1 |  |  | 1 |  |  |  | 1 |  |  |  |  | 1 |  |  |  |  | 1 |  |  |  |  |  | 1 | 1 |  |  |  | 1 | 1 |  |  |  |  |  | 1 |  |  |  |  | 1 |  |  | 1 |  | 1 |  |  |  | 1 |  |  | 1 |  |  |  | 1 |  | 1 |  |  | 1 |  |  |  | 1 |  |  |
| 38 | 1 | 4 | 2 | 2 |  | 1 |  |  | 1 |  |  |  |  |  |  |  | 1 | 1 |  |  |  |  | 1 |  |  |  |  | 1 |  | 1 | 1 |  |  |  | 1 |  |  |  |  |  | 1 |  |  |  | 1 |  |  |  | 1 |  | 1 |  |  |  |  | 1 |  | 1 |  |  |  | 1 |  | 1 |  |  | 1 |  |  |  | 1 |  |  |  | 1 |  |  | 1 |  |  |  | 1 |  |  |  |  | 1 |  |  |  |  | 1 |  |  |  | 1 |  |  | 1 | 1 |  |  | 1 | 1 |  |  |  |  |  | 1 |  |  |  |  | 1 |  |  | 1 |  | 1 |  |  |  |  | 1 |  | 1 |  |  |  | 1 |  | 1 |  |  | 1 |  |  |  | 1 |  |  |
| 39 | 2 | 3 | 2 | 6 |  | 1 |  |  | 1 |  |  |  |  |  |  | 1 |  |  | 1 |  |  |  | 1 |  |  |  |  |  | 1 | 1 | 1 |  |  | 1 | 1 |  |  |  |  |  |  | 1 |  |  |  |  | 1 | 1 | 1 |  | 1 |  |  |  | 1 |  |  | 1 |  |  |  | 1 |  |  |  |  | 1 | 1 |  |  | 1 |  |  |  | 1 |  |  | 1 |  |  |  | 1 |  |  |  |  | 1 |  |  |  |  | 1 |  |  |  |  |  | 1 | 1 | 1 |  |  | 1 | 1 |  |  |  |  |  |  | 1 |  |  |  | 1 |  |  | 1 |  | 1 |  |  |  | 1 |  |  | 1 |  |  |  | 1 |  |  |  |  | 1 |  |  |  | 1 |  |  |
| 40 | 1 | 3 | 4 | 1 |  | 1 |  |  | 1 |  |  |  |  |  |  | 1 |  | 1 |  |  |  |  | 1 |  |  |  |  | 1 |  |  | 1 |  |  | 1 | 1 |  |  |  |  |  |  | 1 |  | 1 |  |  |  |  | 1 | 1 |  |  |  |  | 1 |  |  | 1 |  |  |  | 1 |  |  |  |  | 1 |  |  |  | 1 |  |  |  | 1 |  |  | 1 |  |  |  | 1 |  |  |  |  | 1 |  |  |  |  | 1 |  |  |  |  | 1 |  |  | 1 |  |  | 1 | 1 |  |  |  |  |  |  | 1 |  |  |  | 1 |  |  | 1 |  | 1 |  |  |  | 1 |  |  | 1 |  |  |  | 1 |  |  |  |  | 1 |  |  |  | 1 |  |  |
| 41 | 1 | 4 | 2 | 1 | 1 |  |  |  | 1 |  |  |  |  |  |  | 1 |  | 1 |  |  |  |  |  | 1 |  |  | 1 |  |  |  | 1 |  |  | 1 | 1 |  |  |  |  |  |  | 1 |  | 1 |  |  |  |  | 1 | 1 | 1 |  |  |  | 1 |  |  | 1 |  |  |  | 1 |  | 1 |  |  |  | 1 |  |  | 1 |  |  | 1 |  |  |  | 1 |  |  |  |  |  |  | 1 |  | 1 |  |  |  |  | 1 |  |  |  | 1 |  |  |  | 1 |  |  | 1 | 1 |  |  |  |  |  |  | 1 |  |  |  | 1 |  |  | 1 | 1 | 1 |  |  |  | 1 |  |  | 1 |  |  |  | 1 |  | 1 |  |  | 1 | 1 |  |  | 1 |  |  |
| 42 | 2 | 2 | 3 | 1 |  | 1 |  |  |  |  |  | 1 |  |  |  |  | 1 | 1 |  |  |  |  |  | 1 |  |  |  |  | 1 |  |  |  |  | 1 | 1 | 1 |  |  |  |  |  | 1 |  |  |  |  | 1 | 1 | 1 | 1 | 1 |  |  |  |  | 1 |  | 1 |  |  |  | 1 |  | 1 |  |  | 1 | 1 |  |  | 1 |  |  |  | 1 |  |  | 1 |  |  |  |  |  |  |  | 1 | 1 |  |  |  |  | 1 |  |  |  |  |  | 1 |  |  |  |  | 1 | 1 | 1 |  |  |  |  |  | 1 |  |  |  | 1 |  | 1 | 1 | 1 | 1 |  |  |  | 1 | 1 |  | 1 |  |  |  | 1 |  | 1 |  |  | 1 | 1 |  |  | 1 |  |  |
| 43 | 1 | 3 | 5 | 3 |  |  | 1 |  | 1 |  |  |  |  |  |  | 1 |  |  | 1 |  |  |  | 1 |  |  |  |  |  | 1 | 1 | 1 |  |  | 1 | 1 |  |  |  |  |  |  | 1 |  |  | 1 |  |  |  | 1 |  | 1 |  |  |  | 1 | 1 |  | 1 |  |  |  | 1 |  | 1 |  |  |  | 1 |  |  | 1 |  |  |  | 1 |  |  | 1 |  |  |  | 1 |  |  |  |  | 1 |  |  |  |  | 1 |  |  |  |  |  | 1 | 1 | 1 |  |  | 1 | 1 |  |  |  |  |  |  | 1 |  |  |  | 1 |  |  | 1 |  | 1 |  |  |  | 1 |  |  | 1 |  |  |  | 1 |  | 1 |  |  | 1 | 1 |  |  | 1 |  |  |
| 44 | 2 | 4 | 4 | 3 |  |  | 1 |  |  |  |  | 1 |  |  |  |  | 1 |  | 1 |  |  |  |  | 1 |  |  |  |  | 1 |  | 1 |  |  | 1 | 1 |  |  |  |  |  | 1 |  |  |  |  |  | 1 | 1 | 1 |  | 1 |  |  |  |  |  | 1 |  |  | 1 |  | 1 |  | 1 |  |  | 1 | 1 |  |  | 1 |  |  |  | 1 |  |  | 1 |  |  |  | 1 |  |  |  |  | 1 |  |  |  |  | 1 |  |  |  | 1 | 1 |  | 1 | 1 |  |  | 1 | 1 | 1 |  |  |  |  |  | 1 |  |  |  | 1 |  | 1 | 1 | 1 | 1 |  |  |  | 1 | 1 |  | 1 |  |  |  | 1 |  | 1 |  |  | 1 | 1 |  |  | 1 |  |  |
| 45 | 1 | 3 | 2 | 1 |  | 1 |  |  | 1 |  |  |  |  |  |  | 1 |  | 1 |  |  |  |  | 1 |  |  |  |  |  | 1 | 1 | 1 |  |  | 1 | 1 |  |  |  |  |  | 1 |  |  | 1 |  |  |  |  |  | 1 |  |  |  |  | 1 |  |  | 1 |  |  |  | 1 |  | 1 |  |  |  | 1 |  |  | 1 |  |  |  | 1 |  |  | 1 |  |  |  | 1 |  |  |  |  | 1 |  |  |  |  | 1 |  |  |  |  |  | 1 | 1 | 1 |  |  | 1 | 1 |  |  |  |  |  | 1 |  |  |  |  | 1 |  |  | 1 |  | 1 |  |  |  | 1 |  |  | 1 |  |  |  | 1 |  | 1 |  |  | 1 | 1 |  |  | 1 |  |  |
| 46 | 1 | 4 | 4 | 1 | 1 |  |  |  | 1 |  |  |  |  |  |  | 1 |  | 1 |  |  |  |  | 1 |  |  |  |  |  | 1 | 1 | 1 |  |  | 1 | 1 |  |  |  |  |  | 1 |  |  | 1 |  |  |  | 1 | 1 |  | 1 |  |  |  | 1 |  |  |  |  | 1 |  |  |  | 1 |  |  | 1 |  |  |  | 1 |  |  |  | 1 |  |  | 1 |  |  |  | 1 |  |  |  |  | 1 |  |  |  |  | 1 |  |  |  |  |  | 1 | 1 | 1 |  |  | 1 |  |  |  |  |  |  | 1 |  |  | 1 |  | 1 |  | 1 | 1 |  | 1 |  |  |  | 1 |  |  | 1 |  |  |  | 1 |  | 1 |  |  | 1 |  |  |  | 1 |  |  |
| 47 | 2 | 3 | 3 | 1 |  | 1 |  |  | 1 |  |  |  |  |  |  | 1 |  | 1 |  |  |  |  | 1 |  |  |  |  | 1 |  |  | 1 |  |  | 1 | 1 | 1 |  |  |  |  | 1 |  |  | 1 |  |  |  | 1 | 1 |  | 1 |  |  |  | 1 |  |  | 1 |  |  |  | 1 |  | 1 |  |  | 1 | 1 |  |  | 1 |  |  |  | 1 |  |  | 1 |  |  |  | 1 |  |  |  |  | 1 |  |  |  |  | 1 |  |  |  |  | 1 |  |  | 1 |  |  | 1 | 1 | 1 |  |  |  |  | 1 |  |  |  |  | 1 |  | 1 | 1 |  | 1 |  |  |  | 1 |  |  | 1 |  |  |  | 1 |  | 1 |  |  | 1 |  |  |  | 1 |  |  |
| 48 | 2 | 4 | 1 | 1 |  |  | 1 |  |  |  |  | 1 |  |  |  |  | 1 | 1 |  |  |  |  |  | 1 |  |  |  |  | 1 |  | 1 |  |  | 1 | 1 | 1 |  |  |  |  |  | 1 |  | 1 |  |  |  |  | 1 |  | 1 |  |  |  | 1 |  |  | 1 |  |  |  | 1 |  | 1 |  |  |  |  |  | 1 | 1 |  |  |  | 1 |  |  | 1 |  |  |  |  |  |  |  | 1 | 1 |  |  |  |  | 1 |  |  |  |  |  | 1 |  | 1 |  |  | 1 | 1 | 1 |  |  |  |  |  | 1 |  |  |  | 1 |  |  | 1 |  | 1 |  |  |  | 1 |  |  | 1 |  |  |  | 1 |  | 1 |  |  | 1 |  |  |  | 1 |  |  |
| 49 | 2 | 3 | 4 | 4 | 1 |  |  |  | 1 |  |  |  |  |  |  |  | 1 |  | 1 |  |  |  |  | 1 |  | 1 |  |  |  |  |  |  |  | 1 | 1 | 1 |  |  | 1 |  |  |  |  | 1 |  |  |  | 1 | 1 |  | 1 |  |  |  | 1 |  |  |  | 1 |  |  | 1 |  | 1 |  |  | 1 | 1 |  |  | 1 |  |  |  | 1 |  |  | 1 |  |  |  | 1 |  |  |  |  | 1 |  |  |  |  | 1 |  |  | 1 |  |  |  |  |  |  |  | 1 | 1 | 1 |  |  | 1 |  |  |  |  | 1 |  |  |  | 1 |  |  |  |  |  |  | 1 |  |  | 1 |  |  |  | 1 |  | 1 |  |  | 1 | 1 |  |  | 1 |  |  |
| 50 | 1 | 4 | 4 | 3 |  | 1 |  |  | 1 |  |  |  | 1 |  |  |  |  |  | 1 |  |  |  | 1 |  |  |  |  |  | 1 | 1 |  |  |  | 1 | 1 |  |  |  |  |  | 1 |  |  | 1 |  |  |  |  | 1 | 1 | 1 |  |  |  |  | 1 |  | 1 |  |  |  | 1 |  | 1 |  |  | 1 |  |  |  | 1 |  |  |  | 1 |  |  | 1 |  |  |  | 1 |  |  |  |  | 1 |  |  |  |  | 1 |  |  |  |  |  | 1 | 1 |  |  |  |  | 1 | 1 |  |  |  |  | 1 |  |  |  |  | 1 |  |  | 1 |  | 1 |  |  |  | 1 |  |  | 1 |  |  |  | 1 |  | 1 |  |  | 1 |  |  |  | 1 |  |  |
| 51 | 1 | 3 | 2 | 1 |  | 1 |  | 1 |  |  |  |  |  |  |  | 1 |  |  | 1 |  |  |  | 1 |  |  |  |  | 1 |  | 1 |  |  |  | 1 | 1 |  |  |  |  |  | 1 |  |  | 1 |  |  |  |  | 1 |  | 1 |  |  |  | 1 |  |  |  | 1 |  |  | 1 |  | 1 |  |  | 1 | 1 |  |  | 1 |  |  |  | 1 |  |  | 1 |  |  |  |  |  |  | 1 |  | 1 |  |  |  |  | 1 |  |  |  |  | 1 |  | 1 |  |  |  | 1 | 1 |  |  |  |  |  | 1 |  |  |  |  | 1 |  |  | 1 |  | 1 |  |  |  | 1 |  |  | 1 |  |  |  | 1 |  | 1 |  |  | 1 | 1 |  |  | 1 |  |  |
| 52 | 1 | 3 | 4 | 6 |  | 1 |  | 1 |  |  |  |  |  |  |  |  | 1 | 1 |  |  |  |  |  |  | 1 |  |  |  | 1 | 1 | 1 | 1 | 1 | 1 | 1 | 1 |  |  |  |  |  |  | 1 |  |  |  | 1 | 1 | 1 | 1 | 1 |  |  |  |  |  | 1 | 1 |  |  |  | 1 |  | 1 |  |  | 1 | 1 |  |  | 1 |  |  |  | 1 |  |  | 1 |  |  |  | 1 |  |  |  |  | 1 |  |  |  |  | 1 |  |  |  |  |  | 1 | 1 | 1 | 1 | 1 | 1 | 1 | 1 |  |  |  |  | 1 |  |  |  |  | 1 |  | 1 | 1 | 1 | 1 |  |  |  | 1 |  |  | 1 |  |  |  | 1 |  | 1 |  |  | 1 |  |  |  | 1 |  |  |
| 53 | 1 | 1 | 1 | 1 |  | 1 |  |  | 1 |  |  |  |  |  |  | 1 |  | 1 |  |  |  |  |  | 1 |  |  |  |  | 1 |  |  |  |  | 1 |  |  |  |  |  |  |  | 1 |  |  |  |  | 1 |  |  |  | 1 |  |  |  | 1 | 1 |  | 1 |  |  |  | 1 |  | 1 |  |  | 1 | 1 |  |  | 1 |  |  |  | 1 |  |  | 1 |  |  |  | 1 |  |  |  |  | 1 |  |  |  |  |  | 1 |  |  |  |  | 1 |  |  |  |  | 1 | 1 |  |  |  |  |  |  | 1 |  |  |  | 1 |  |  |  |  | 1 |  |  |  | 1 |  |  | 1 |  |  |  | 1 |  | 1 |  |  | 1 |  |  |  | 1 |  |  |
| 54 | 2 | 4 | 2 | 3 |  | 1 |  |  | 1 |  |  |  |  |  |  | 1 |  | 1 |  |  |  |  | 1 |  |  |  |  |  | 1 |  |  |  |  | 1 | 1 |  |  |  |  |  |  | 1 |  |  |  |  | 1 |  |  |  | 1 |  |  |  |  |  | 1 | 1 |  |  |  | 1 |  | 1 |  |  | 1 | 1 |  |  | 1 |  |  |  | 1 |  |  | 1 |  |  |  |  |  |  | 1 |  | 1 |  |  |  |  | 1 |  |  |  |  |  | 1 |  | 1 |  |  | 1 | 1 |  |  |  |  |  | 1 |  |  |  |  | 1 |  |  | 1 |  | 1 |  |  |  | 1 |  |  | 1 |  |  |  | 1 |  |  |  |  | 1 | 1 |  |  | 1 |  |  |
| 55 | 1 | 3 | 4 | 6 |  | 1 |  |  | 1 |  |  |  |  |  |  | 1 |  | 1 |  |  |  |  |  | 1 |  |  |  |  | 1 | 1 | 1 |  |  | 1 | 1 |  |  |  |  |  | 1 |  |  |  | 1 |  |  |  |  |  | 1 |  |  |  | 1 |  |  |  | 1 |  |  | 1 |  |  |  |  | 1 |  |  |  | 1 |  |  |  | 1 |  |  | 1 |  |  |  |  |  |  | 1 |  | 1 |  |  |  |  | 1 |  |  |  |  |  | 1 | 1 | 1 |  |  | 1 | 1 |  |  |  |  |  | 1 |  |  |  |  | 1 |  |  | 1 |  | 1 |  |  |  | 1 |  |  | 1 |  |  |  | 1 |  |  |  |  | 1 |  |  |  | 1 |  |  |
| 56 | 2 | 3 | 4 | 2 |  | 1 |  |  | 1 |  |  |  |  |  |  | 1 |  | 1 |  |  |  |  |  | 1 |  |  | 1 |  |  |  |  |  | 1 | 1 |  |  |  |  | 1 |  |  |  |  | 1 |  |  |  |  | 1 |  |  |  |  |  | 1 |  |  | 1 |  |  |  | 1 |  |  |  |  | 1 |  |  |  | 1 |  |  |  | 1 |  |  | 1 |  |  |  | 1 |  |  |  |  | 1 |  |  |  |  | 1 |  |  |  | 1 |  |  |  | 1 |  |  | 1 |  |  |  |  | 1 |  |  |  |  |  |  | 1 |  |  | 1 |  | 1 |  |  |  | 1 |  |  | 1 |  |  |  | 1 |  |  |  |  | 1 |  |  |  | 1 |  |  |
| 57 | 1 | 2 | 2 | 2 | 1 |  |  |  | 1 |  |  |  | 1 |  |  |  |  | 1 |  |  |  |  | 1 |  |  |  |  |  | 1 | 1 | 1 |  |  | 1 | 1 |  |  |  |  |  | 1 |  |  | 1 |  |  |  |  | 1 |  | 1 |  |  |  | 1 |  |  |  | 1 |  |  | 1 |  |  |  |  | 1 |  |  |  | 1 |  |  | 1 |  |  |  | 1 |  |  |  | 1 |  |  |  |  | 1 |  |  |  |  | 1 |  |  |  |  |  | 1 | 1 | 1 |  |  | 1 |  |  |  |  |  |  | 1 |  |  |  |  | 1 |  |  | 1 |  | 1 |  |  | 1 |  |  |  | 1 |  |  |  | 1 |  |  |  |  | 1 |  |  |  | 1 |  |  |
| 58 | 2 | 4 | 4 | 1 |  |  | 1 |  | 1 |  |  |  |  |  |  |  | 1 | 1 |  |  |  |  |  | 1 |  |  |  | 1 |  |  |  |  |  | 1 | 1 |  |  |  |  |  |  | 1 |  |  |  |  | 1 |  | 1 | 1 | 1 |  |  |  |  | 1 |  | 1 |  |  |  | 1 |  | 1 |  | 1 |  |  |  |  | 1 |  |  |  | 1 |  |  | 1 |  |  |  | 1 |  |  |  |  | 1 |  |  |  |  | 1 |  |  |  |  | 1 |  |  |  |  |  | 1 | 1 |  |  |  |  |  |  | 1 |  |  |  | 1 |  |  | 1 |  | 1 |  |  |  | 1 | 1 |  | 1 |  |  |  | 1 |  | 1 |  |  | 1 | 1 |  |  | 1 |  |  |
| 59 | 1 | 3 | 4 | 3 |  | 1 |  |  | 1 |  |  |  |  |  |  | 1 |  | 1 |  |  |  |  |  | 1 |  |  | 1 |  |  | 1 | 1 |  |  | 1 | 1 |  |  |  |  |  |  | 1 |  | 1 |  |  |  |  | 1 |  | 1 |  |  |  |  | 1 |  | 1 |  |  |  | 1 |  | 1 |  |  | 1 | 1 |  |  | 1 |  |  |  | 1 |  |  | 1 |  |  |  | 1 |  |  |  |  | 1 |  |  |  |  | 1 |  |  |  | 1 |  |  | 1 | 1 |  |  | 1 | 1 |  |  |  |  |  |  | 1 |  | 1 |  |  |  | 1 | 1 |  | 1 |  |  |  |  | 1 |  | 1 |  |  |  | 1 |  | 1 |  |  | 1 |  |  |  | 1 |  |  |
| 60 | 2 | 4 | 4 | 1 |  |  | 1 |  | 1 |  |  |  |  |  |  |  | 1 | 1 |  |  |  |  | 1 |  |  |  |  |  | 1 | 1 | 1 |  |  |  |  |  |  |  |  |  | 1 |  |  |  |  |  | 1 |  | 1 |  |  |  |  |  |  |  | 1 | 1 |  |  |  | 1 |  |  |  |  |  |  |  | 1 |  |  | 1 |  | 1 |  |  | 1 |  |  |  | 1 |  |  |  |  | 1 |  |  |  |  | 1 |  |  |  |  |  | 1 | 1 | 1 |  |  | 1 |  |  |  |  |  |  | 1 |  |  |  |  | 1 |  |  | 1 |  |  |  |  |  |  |  | 1 | 1 |  |  |  | 1 |  |  |  |  | 1 |  |  |  | 1 |  |  |
| 61 | 1 | 2 | 2 | 1 |  | 1 |  | 1 |  |  |  |  |  |  |  |  | 1 | 1 |  |  |  |  |  | 1 |  |  |  |  | 1 |  | 1 |  |  |  | 1 | 1 |  |  |  |  | 1 |  |  | 1 |  |  |  |  | 1 |  | 1 |  |  |  |  |  | 1 | 1 |  |  |  | 1 |  | 1 |  |  |  |  |  | 1 | 1 |  |  |  | 1 |  |  | 1 |  |  |  |  |  |  |  | 1 | 1 |  |  |  |  | 1 |  |  |  |  |  | 1 |  | 1 |  |  | 1 | 1 | 1 |  |  |  |  | 1 |  |  |  |  | 1 |  |  | 1 |  | 1 |  |  |  | 1 |  |  | 1 |  |  |  | 1 |  | 1 |  |  | 1 | 1 |  |  | 1 |  |  |
| 62 | 2 | 5 | 2 | 1 |  | 1 |  |  |  |  |  | 1 |  |  |  |  | 1 |  | 1 |  |  |  |  | 1 |  |  |  |  | 1 |  | 1 |  |  |  | 1 | 1 |  |  |  |  | 1 |  |  |  |  |  | 1 |  | 1 |  | 1 |  |  |  | 1 |  |  | 1 |  |  |  | 1 |  | 1 |  |  | 1 | 1 |  |  | 1 |  |  |  | 1 |  |  | 1 |  |  |  | 1 |  |  |  |  | 1 |  |  |  |  |  | 1 |  |  |  |  | 1 |  | 1 |  |  | 1 | 1 | 1 |  |  |  |  | 1 |  |  |  |  | 1 |  |  | 1 |  | 1 |  |  |  | 1 |  |  | 1 |  |  |  | 1 |  | 1 |  |  | 1 | 1 |  |  | 1 |  |  |
| 63 | 2 | 2 | 3 | 1 |  | 1 |  | 1 |  |  |  |  |  |  |  | 1 |  |  | 1 |  |  |  |  | 1 |  |  |  |  | 1 | 1 |  |  |  | 1 | 1 | 1 |  |  |  |  | 1 |  |  |  |  |  | 1 |  | 1 |  |  |  |  |  |  |  | 1 |  | 1 |  |  | 1 |  |  |  |  |  | 1 |  |  | 1 |  |  |  | 1 |  |  | 1 |  |  |  |  |  |  | 1 |  | 1 |  |  |  |  | 1 |  |  |  |  |  | 1 | 1 |  |  |  | 1 |  | 1 |  |  |  |  | 1 |  |  |  |  | 1 |  |  | 1 |  |  |  |  |  |  |  | 1 | 1 |  |  |  | 1 |  |  |  |  | 1 | 1 |  |  | 1 |  |  |
| 64 | 2 | 3 | 3 | 1 | 1 |  |  |  | 1 |  |  |  |  |  |  |  | 1 |  | 1 |  |  |  |  | 1 |  |  |  |  | 1 |  | 1 |  |  | 1 | 1 |  |  |  |  |  | 1 |  |  | 1 |  |  |  |  | 1 |  | 1 |  |  |  | 1 |  |  |  | 1 |  |  | 1 |  |  |  |  | 1 |  |  |  | 1 |  |  |  | 1 |  |  | 1 |  |  |  | 1 |  |  |  |  | 1 |  |  |  |  | 1 |  |  |  |  |  | 1 |  | 1 |  |  | 1 | 1 |  |  |  |  |  | 1 |  |  |  |  | 1 |  |  | 1 |  | 1 |  |  | 1 |  |  |  |  | 1 |  |  | 1 |  |  |  |  | 1 |  |  |  | 1 |  |  |
| 65 | 1 | 3 | 3 | 1 | 1 |  |  |  | 1 |  |  |  |  |  |  | 1 |  | 1 |  |  |  |  | 1 |  |  |  |  | 1 |  |  | 1 |  |  | 1 | 1 |  |  |  |  |  | 1 |  |  | 1 |  |  |  | 1 | 1 |  | 1 |  |  |  |  | 1 |  | 1 |  |  |  | 1 |  |  |  |  | 1 |  |  |  | 1 |  |  |  | 1 |  |  | 1 |  |  |  | 1 |  |  |  |  | 1 |  |  |  |  | 1 |  |  |  |  | 1 |  |  | 1 |  |  | 1 | 1 |  |  |  |  |  | 1 |  |  |  |  | 1 |  | 1 | 1 |  | 1 |  |  |  |  | 1 |  | 1 |  |  |  | 1 |  |  |  |  | 1 |  |  |  | 1 |  |  |
| 66 | 1 | 4 | 2 | 1 | 1 |  |  |  | 1 |  |  |  |  |  |  | 1 |  | 1 |  |  |  |  |  | 1 |  |  |  |  | 1 |  | 1 |  |  | 1 | 1 |  |  |  |  |  |  | 1 |  |  | 1 |  |  |  |  |  | 1 |  |  | 1 |  |  |  |  |  | 1 |  | 1 |  |  |  |  |  |  |  | 1 | 1 |  |  |  | 1 |  |  | 1 |  |  |  |  |  |  | 1 |  | 1 |  |  |  |  | 1 |  |  |  |  |  | 1 |  | 1 |  |  | 1 | 1 |  |  |  |  |  |  | 1 |  |  |  | 1 |  |  |  |  | 1 |  |  |  | 1 |  |  | 1 |  |  |  | 1 |  |  |  |  | 1 | 1 |  |  | 1 |  |  |
| 67 | 1 | 4 | 4 | 1 |  | 1 |  |  |  | 1 |  |  | 1 |  |  |  |  |  | 1 |  |  |  | 1 |  |  | 1 |  |  |  | 1 | 1 |  |  | 1 | 1 |  |  |  | 1 |  |  |  |  |  | 1 |  |  |  | 1 |  | 1 |  |  |  | 1 |  |  | 1 |  |  |  |  |  | 1 |  |  |  | 1 |  |  | 1 |  |  |  | 1 |  |  | 1 |  |  |  | 1 |  |  |  |  | 1 |  |  |  |  | 1 |  |  |  |  |  | 1 | 1 | 1 |  |  | 1 | 1 |  |  |  | 1 |  |  |  |  |  |  | 1 |  |  | 1 |  | 1 |  |  |  | 1 |  |  | 1 |  |  |  | 1 |  | 1 |  |  | 1 | 1 |  |  | 1 |  |  |
| 68 | 1 | 4 | 1 | 6 |  | 1 |  |  | 1 |  |  |  |  |  |  | 1 |  | 1 |  |  |  |  |  | 1 |  |  |  |  | 1 | 1 | 1 |  |  | 1 | 1 |  |  |  |  |  | 1 |  |  | 1 |  |  |  |  | 1 |  | 1 |  |  |  | 1 |  |  | 1 |  |  |  | 1 |  |  |  |  | 1 | 1 |  |  | 1 |  |  |  | 1 |  |  | 1 |  |  |  | 1 |  |  |  |  | 1 |  |  |  |  | 1 |  |  |  | 1 |  |  | 1 | 1 |  |  | 1 | 1 |  |  |  |  |  | 1 |  |  |  |  | 1 |  |  | 1 |  | 1 |  |  |  | 1 |  |  | 1 |  |  |  | 1 |  |  |  |  | 1 |  |  |  | 1 |  |  |
| 69 | 2 | 3 | 1 | 1 |  |  | 1 |  | 1 |  |  |  |  |  |  |  | 1 | 1 |  |  |  |  |  | 1 |  |  |  |  | 1 |  | 1 |  |  | 1 | 1 | 1 |  |  |  |  |  |  | 1 |  |  |  | 1 |  | 1 |  | 1 |  |  |  |  |  | 1 |  |  | 1 |  | 1 |  | 1 |  |  |  |  |  | 1 | 1 |  |  |  | 1 |  |  | 1 |  |  |  |  |  |  |  | 1 | 1 |  |  |  |  | 1 |  |  |  |  |  | 1 |  | 1 |  |  | 1 | 1 |  |  |  |  |  | 1 |  |  |  |  | 1 |  |  | 1 |  | 1 |  |  |  | 1 |  |  |  |  | 1 |  | 1 |  | 1 |  |  | 1 |  |  |  | 1 |  |  |
| 70 | 2 | 2 | 2 | 3 |  | 1 |  |  | 1 |  |  |  |  |  |  | 1 |  | 1 |  |  |  |  |  | 1 |  |  | 1 |  |  |  |  |  |  | 1 | 1 |  |  |  |  |  | 1 |  |  |  | 1 |  |  |  | 1 |  | 1 |  |  |  |  |  | 1 | 1 |  |  |  | 1 |  |  |  |  | 1 | 1 |  |  | 1 |  |  |  | 1 |  |  | 1 |  |  |  |  |  |  | 1 |  | 1 |  |  |  |  | 1 |  |  |  | 1 |  |  |  |  |  |  | 1 |  |  |  |  |  |  | 1 |  |  |  |  | 1 |  |  | 1 |  | 1 |  |  |  | 1 |  |  | 1 |  |  |  | 1 |  | 1 |  |  | 1 | 1 |  |  | 1 |  |  |
| 71 | 1 | 5 | 2 | 1 | 1 |  |  |  | 1 |  |  |  |  |  |  | 1 |  | 1 |  |  |  |  |  | 1 |  |  | 1 |  |  |  |  |  |  | 1 | 1 |  |  |  |  |  | 1 |  |  |  |  |  | 1 |  | 1 |  | 1 |  |  |  |  |  | 1 | 1 |  |  |  | 1 |  | 1 |  |  |  |  |  | 1 | 1 |  |  |  | 1 |  |  | 1 |  |  |  |  |  |  | 1 |  | 1 |  |  |  |  | 1 |  |  |  | 1 |  |  |  |  |  |  | 1 |  |  |  |  |  |  | 1 |  |  |  |  | 1 |  |  | 1 |  | 1 |  |  |  | 1 | 1 |  | 1 |  |  |  | 1 |  | 1 |  |  | 1 |  |  |  | 1 |  |  |
| 72 | 2 | 2 | 4 | 1 | 1 |  |  |  | 1 |  |  |  |  |  |  |  | 1 | 1 |  |  |  |  |  |  | 1 |  |  |  | 1 |  |  |  |  | 1 | 1 |  |  |  |  |  | 1 |  |  | 1 |  |  |  |  |  |  | 1 |  |  |  | 1 |  |  | 1 |  |  |  | 1 |  | 1 |  |  |  |  |  | 1 | 1 |  |  |  | 1 |  |  | 1 |  |  |  | 1 |  |  |  |  | 1 |  |  |  |  | 1 |  |  |  |  |  | 1 |  | 1 |  |  | 1 | 1 |  |  |  |  |  | 1 |  |  |  |  | 1 |  |  |  |  | 1 |  |  |  | 1 |  |  | 1 |  |  |  | 1 |  | 1 |  |  | 1 |  |  |  | 1 |  |  |
| 73 | 1 | 4 | 3 | 3 |  | 1 |  |  | 1 |  |  |  |  |  |  | 1 |  | 1 |  |  |  |  | 1 |  |  |  |  |  | 1 | 1 | 1 |  |  | 1 | 1 |  |  |  |  |  |  | 1 |  | 1 |  |  |  |  |  |  | 1 |  |  |  | 1 | 1 |  |  |  | 1 |  | 1 |  |  |  |  |  | 1 |  |  | 1 |  |  |  | 1 |  |  | 1 |  |  |  |  |  |  | 1 |  | 1 |  |  |  |  | 1 |  |  |  |  |  | 1 | 1 | 1 |  |  | 1 | 1 |  |  |  |  |  |  | 1 |  |  |  | 1 |  |  |  |  | 1 |  |  |  | 1 |  |  | 1 |  |  |  | 1 |  |  |  |  | 1 | 1 |  |  | 1 |  |  |
| 74 | 2 | 1 | 1 | 1 |  | 1 |  |  | 1 |  |  |  |  |  |  | 1 |  | 1 |  |  |  |  | 1 |  |  |  |  |  | 1 |  | 1 |  |  | 1 | 1 | 1 |  |  |  |  | 1 |  |  |  |  |  | 1 |  |  |  | 1 |  |  |  | 1 |  |  | 1 |  |  |  | 1 |  | 1 |  |  |  |  |  | 1 | 1 |  |  |  | 1 |  |  | 1 |  |  |  |  |  |  | 1 |  | 1 |  |  |  |  | 1 |  |  |  |  |  | 1 | 1 | 1 |  |  | 1 | 1 | 1 |  |  |  |  | 1 |  |  |  |  | 1 |  |  | 1 |  | 1 |  |  |  | 1 |  |  | 1 |  |  |  | 1 |  | 1 |  |  |  |  |  | 1 | 1 |  |  |
| 75 | 1 | 3 | 2 | 1 |  | 1 |  | 1 |  |  |  |  |  |  |  | 1 |  |  | 1 |  |  |  | 1 |  |  |  |  |  | 1 | 1 |  |  |  | 1 | 1 |  |  |  |  |  | 1 |  |  |  |  | 1 |  | 1 | 1 |  | 1 |  |  |  |  | 1 |  | 1 |  |  |  | 1 |  | 1 |  |  | 1 |  |  |  | 1 |  |  |  | 1 |  |  | 1 |  |  |  | 1 |  |  |  |  | 1 |  |  |  |  | 1 |  |  |  |  | 1 |  |  | 1 |  |  | 1 | 1 | 1 |  |  |  |  |  | 1 |  |  |  | 1 |  | 1 | 1 |  | 1 |  |  |  | 1 | 1 |  | 1 |  |  |  | 1 |  | 1 |  |  | 1 |  |  |  | 1 |  |  |
| 76 | 1 | 3 | 4 | 6 |  | 1 |  |  | 1 |  |  |  |  |  |  | 1 |  | 1 |  |  |  |  | 1 |  |  |  | 1 |  |  | 1 | 1 |  |  | 1 | 1 |  |  |  |  |  |  |  | 1 |  |  |  | 1 | 1 | 1 | 1 | 1 |  |  |  | 1 | 1 |  | 1 |  |  |  | 1 |  | 1 |  |  | 1 | 1 |  |  | 1 |  |  |  | 1 |  |  | 1 |  |  |  | 1 |  |  |  |  | 1 |  |  |  |  | 1 |  |  |  | 1 |  |  | 1 | 1 |  |  | 1 | 1 |  |  |  |  |  |  | 1 |  |  |  | 1 |  | 1 |  | 1 | 1 |  |  |  |  | 1 |  | 1 |  |  |  | 1 |  | 1 |  |  | 1 |  |  |  | 1 |  |  |
| 77 | 1 | 4 | 2 | 1 |  |  | 1 |  |  |  |  | 1 |  |  |  | 1 |  | 1 |  |  |  |  |  | 1 |  |  |  |  | 1 |  |  |  |  | 1 | 1 |  |  |  |  |  |  | 1 |  |  |  |  | 1 |  | 1 | 1 | 1 |  |  |  | 1 |  |  | 1 |  |  |  | 1 |  | 1 |  |  |  |  |  | 1 | 1 |  |  |  | 1 |  |  | 1 |  |  |  | 1 |  |  |  |  | 1 |  |  |  |  | 1 |  |  |  |  | 1 |  | 1 | 1 |  |  | 1 | 1 | 1 |  |  |  |  |  | 1 |  |  |  | 1 |  | 1 | 1 | 1 | 1 |  |  |  | 1 |  |  | 1 |  |  |  | 1 |  | 1 |  |  | 1 | 1 |  |  | 1 |  |  |
| 78 | 2 | 2 | 2 | 1 | 1 |  |  |  | 1 |  |  |  |  |  |  | 1 |  | 1 |  |  |  |  |  | 1 |  |  |  |  | 1 |  |  |  |  | 1 | 1 |  |  |  |  |  |  | 1 |  |  |  | 1 |  |  | 1 |  | 1 |  |  |  |  |  | 1 | 1 |  |  |  |  |  | 1 |  |  | 1 |  |  |  | 1 |  |  |  | 1 |  |  | 1 |  |  |  | 1 |  |  |  |  | 1 |  |  |  |  | 1 |  |  |  | 1 |  |  | 1 | 1 |  |  | 1 | 1 | 1 |  |  |  |  | 1 | 1 |  |  |  | 1 |  | 1 | 1 | 1 | 1 |  |  |  | 1 |  |  | 1 |  |  |  | 1 |  | 1 |  |  | 1 | 1 |  |  | 1 |  |  |
| 79 | 2 | 2 | 2 | 1 |  | 1 |  |  | 1 |  |  |  |  |  |  | 1 |  | 1 |  |  |  |  |  | 1 |  |  |  | 1 |  |  |  |  |  |  | 1 |  |  |  |  |  | 1 |  |  | 1 |  |  |  | 1 | 1 |  | 1 |  |  |  | 1 | 1 |  | 1 |  |  |  | 1 |  | 1 |  |  | 1 |  |  |  | 1 |  |  |  | 1 |  |  | 1 |  |  |  | 1 |  |  |  |  | 1 |  |  |  |  | 1 |  |  |  |  |  | 1 |  |  |  |  | 1 | 1 | 1 |  |  |  |  |  | 1 |  |  |  | 1 |  | 1 |  | 1 | 1 |  |  |  | 1 |  |  | 1 |  |  |  | 1 |  | 1 |  |  | 1 |  |  |  | 1 |  |  |
| 80 | 1 | 4 | 2 | 1 |  | 1 |  |  | 1 |  |  |  | 1 |  |  |  |  | 1 |  |  |  |  |  | 1 |  |  | 1 |  |  | 1 | 1 |  |  | 1 |  |  |  |  |  |  |  | 1 |  | 1 |  |  |  |  | 1 |  | 1 |  |  |  | 1 |  |  | 1 |  |  |  | 1 |  | 1 |  |  | 1 | 1 |  |  | 1 |  |  |  | 1 |  |  | 1 |  |  |  | 1 |  |  |  |  | 1 |  |  |  |  | 1 |  |  |  | 1 |  |  | 1 | 1 |  |  | 1 | 1 | 1 |  |  |  |  |  | 1 |  |  |  | 1 |  | 1 | 1 | 1 | 1 |  |  |  | 1 |  |  | 1 |  |  |  | 1 |  | 1 |  |  | 1 | 1 |  |  | 1 |  |  |
| 81 | 1 | 3 | 2 | 2 |  |  | 1 |  | 1 |  |  |  |  |  |  | 1 |  | 1 |  |  |  |  | 1 |  |  |  |  |  | 1 |  |  |  |  | 1 | 1 | 1 |  |  |  |  | 1 |  |  |  |  |  | 1 |  | 1 |  | 1 |  |  |  |  | 1 |  |  | 1 |  |  |  |  |  | 1 |  |  |  |  | 1 | 1 |  |  |  | 1 |  |  | 1 |  |  |  | 1 |  |  |  |  | 1 |  |  |  |  | 1 |  |  |  | 1 |  |  | 1 | 1 |  |  | 1 | 1 | 1 |  |  |  |  | 1 |  |  |  |  | 1 |  |  | 1 |  | 1 |  |  |  | 1 |  |  | 1 |  |  |  | 1 |  | 1 |  |  |  |  |  | 1 | 1 |  |  |
| 82 | 2 | 4 | 2 | 5 |  | 1 |  |  | 1 |  |  |  |  |  |  | 1 |  | 1 |  |  |  |  | 1 |  |  |  |  | 1 |  | 1 | 1 |  |  | 1 | 1 |  |  |  |  |  | 1 | 1 |  | 1 |  |  |  |  | 1 |  | 1 |  |  |  |  |  | 1 | 1 |  |  |  | 1 |  | 1 |  |  | 1 | 1 |  |  | 1 |  |  |  | 1 |  |  | 1 |  |  |  | 1 |  |  |  |  | 1 |  |  |  |  | 1 |  |  |  | 1 |  |  | 1 | 1 |  |  | 1 | 1 | 1 |  |  |  |  | 1 | 1 |  |  |  | 1 |  | 1 | 1 | 1 | 1 |  |  |  | 1 | 1 |  | 1 |  |  |  | 1 |  | 1 |  |  | 1 | 1 |  |  | 1 |  |  |
| 83 | 1 | 4 | 2 | 1 |  |  | 1 |  |  | 1 |  |  |  |  |  |  | 1 | 1 |  |  |  |  |  | 1 |  |  |  |  | 1 |  |  |  |  |  |  |  | 1 |  |  |  |  |  | 1 |  |  |  | 1 |  | 1 |  | 1 |  |  |  |  |  | 1 |  | 1 |  |  |  |  |  | 1 |  |  |  |  | 1 | 1 |  |  |  | 1 |  |  | 1 |  |  |  | 1 |  |  |  |  | 1 |  |  |  |  | 1 |  |  |  | 1 | 1 |  | 1 | 1 |  |  | 1 | 1 | 1 |  |  |  |  |  | 1 |  |  |  | 1 |  | 1 | 1 | 1 | 1 |  |  |  | 1 | 1 |  | 1 |  |  |  | 1 |  | 1 |  |  | 1 |  |  |  | 1 |  |  |
| 84 | 2 | 5 | 2 | 1 |  | 1 |  |  | 1 |  |  |  |  |  |  | 1 |  | 1 |  |  |  |  |  | 1 |  |  |  |  | 1 | 1 | 1 |  |  | 1 | 1 |  |  |  |  |  | 1 |  |  | 1 |  |  |  |  | 1 |  | 1 |  |  |  | 1 |  |  | 1 |  |  |  | 1 |  | 1 |  |  | 1 |  |  |  | 1 |  |  |  | 1 |  |  | 1 |  |  |  | 1 |  |  |  |  | 1 |  |  |  |  | 1 |  |  |  | 1 | 1 |  | 1 | 1 |  |  | 1 | 1 | 1 |  |  |  |  |  | 1 |  |  |  | 1 |  | 1 | 1 | 1 | 1 |  |  |  | 1 | 1 |  | 1 |  |  |  | 1 |  | 1 |  |  | 1 | 1 |  |  | 1 |  |  |
| 85 | 1 | 3 | 2 | 6 |  | 1 |  |  | 1 |  |  |  |  |  |  |  | 1 | 1 |  |  |  |  |  |  | 1 |  |  |  | 1 | 1 | 1 |  |  | 1 | 1 |  |  |  |  |  | 1 |  |  |  |  |  | 1 | 1 | 1 | 1 | 1 |  |  |  | 1 | 1 |  |  | 1 |  |  | 1 |  |  |  |  |  | 1 |  |  | 1 |  |  |  | 1 |  |  | 1 |  |  |  | 1 |  |  |  |  | 1 |  |  |  |  | 1 |  |  |  | 1 | 1 |  | 1 | 1 |  |  | 1 |  | 1 |  |  |  |  | 1 | 1 |  |  |  | 1 |  | 1 | 1 | 1 | 1 |  |  |  | 1 | 1 |  | 1 |  |  |  | 1 |  | 1 |  |  | 1 | 1 |  |  | 1 |  |  |
| 86 | 1 | 3 | 4 | 2 |  | 1 |  |  | 1 |  |  |  |  |  |  | 1 |  | 1 |  |  |  |  | 1 |  |  |  |  |  | 1 |  |  |  |  | 1 |  |  |  |  |  |  |  | 1 |  | 1 |  |  |  |  |  |  | 1 |  |  |  |  | 1 |  | 1 |  |  |  |  |  | 1 |  |  | 1 |  |  |  | 1 |  |  |  | 1 |  |  | 1 |  |  |  |  |  |  | 1 |  | 1 |  |  |  |  | 1 |  |  |  |  |  | 1 |  |  |  |  |  |  |  | 1 |  |  |  | 1 |  |  | 1 |  |  |  |  | 1 |  |  |  |  |  |  | 1 |  | 1 |  |  |  | 1 |  |  |  |  |  | 1 |  |  | 1 |  |  |
| 87 | 2 | 3 | 3 | 1 |  | 1 |  |  | 1 |  |  |  |  |  |  | 1 |  | 1 |  |  |  |  |  | 1 |  |  |  | 1 |  | 1 | 1 |  |  | 1 | 1 |  |  |  |  |  | 1 | 1 |  | 1 |  |  |  |  | 1 |  | 1 |  |  |  | 1 |  |  | 1 |  |  |  | 1 |  | 1 |  |  | 1 |  |  |  | 1 |  |  |  | 1 |  |  | 1 |  |  |  | 1 |  |  |  |  | 1 |  |  |  |  | 1 |  |  |  | 1 | 1 |  | 1 | 1 |  |  | 1 | 1 | 1 |  |  |  |  | 1 | 1 |  |  |  | 1 |  | 1 | 1 | 1 | 1 |  |  |  | 1 | 1 |  | 1 |  |  |  | 1 |  | 1 |  |  | 1 | 1 |  |  | 1 |  |  |
| 88 | 1 | 2 | 2 | 2 |  | 1 |  |  | 1 |  |  |  |  |  |  | 1 |  | 1 |  |  |  |  |  | 1 |  |  |  |  | 1 | 1 | 1 |  |  | 1 | 1 | 1 |  |  |  |  |  | 1 |  |  |  |  | 1 |  | 1 |  | 1 |  |  |  | 1 |  |  | 1 |  |  |  | 1 |  |  |  |  |  |  |  | 1 | 1 |  |  |  | 1 |  |  | 1 |  |  |  | 1 |  |  |  |  | 1 |  |  |  |  | 1 |  |  |  | 1 |  |  | 1 | 1 |  |  | 1 | 1 | 1 |  |  |  |  | 1 |  |  |  |  | 1 |  | 1 | 1 | 1 | 1 |  |  |  | 1 | 1 |  | 1 |  |  |  | 1 |  | 1 |  |  | 1 |  | 1 |  | 1 |  |  |
| 89 | 2 | 3 | 4 | 2 |  |  | 1 | 1 |  |  |  |  |  |  |  | 1 |  | 1 |  |  |  |  | 1 |  |  |  |  |  | 1 | 1 |  |  |  | 1 | 1 |  |  |  |  |  | 1 |  |  | 1 |  |  |  |  |  | 1 | 1 |  |  |  | 1 |  |  | 1 |  |  |  | 1 |  |  |  |  |  |  |  | 1 |  |  | 1 | 1 |  |  |  | 1 |  |  |  | 1 |  |  |  |  | 1 |  |  |  |  | 1 |  |  |  |  |  | 1 | 1 |  |  |  |  | 1 | 1 |  |  |  |  |  | 1 |  |  |  | 1 |  |  |  | 1 |  |  |  |  | 1 |  |  | 1 |  |  |  | 1 |  |  |  |  |  |  | 1 |  | 1 |  |  |
| 90 | 2 | 3 | 4 | 1 |  |  | 1 |  | 1 |  |  |  |  |  |  | 1 |  | 1 |  |  |  |  |  | 1 |  |  |  |  | 1 |  | 1 |  |  | 1 | 1 |  |  |  |  |  | 1 |  |  |  |  |  | 1 |  | 1 |  | 1 |  |  |  | 1 |  |  | 1 |  |  |  | 1 |  | 1 |  |  |  |  |  | 1 | 1 |  |  | 1 |  |  |  | 1 |  |  |  |  |  |  | 1 |  | 1 |  |  |  |  | 1 |  |  |  |  |  | 1 |  | 1 |  |  |  | 1 | 1 |  |  |  |  |  | 1 |  |  |  | 1 |  | 1 | 1 |  | 1 |  |  |  | 1 |  |  | 1 |  |  |  | 1 |  | 1 |  |  | 1 | 1 |  |  | 1 |  |  |
| 91 | 1 | 2 | 2 | 3 |  |  | 1 |  | 1 |  |  |  |  |  |  | 1 |  | 1 |  |  |  |  |  | 1 |  |  | 1 |  |  |  |  |  |  | 1 | 1 |  |  |  |  |  | 1 |  |  | 1 |  |  |  | 1 | 1 | 1 | 1 |  |  |  |  | 1 |  | 1 |  |  |  | 1 |  | 1 |  |  | 1 | 1 |  |  | 1 |  |  |  | 1 |  |  |  |  | 1 |  |  |  |  | 1 |  | 1 |  |  |  |  |  | 1 |  |  | 1 |  |  |  |  |  |  | 1 | 1 | 1 |  |  |  |  | 1 |  |  | 1 |  |  |  | 1 | 1 | 1 | 1 |  |  |  | 1 | 1 |  | 1 |  |  |  | 1 |  | 1 |  |  | 1 | 1 |  |  | 1 |  |  |
| 92 | 2 | 1 | 1 | 1 |  | 1 |  |  |  | 1 |  |  |  |  |  |  | 1 | 1 |  |  |  |  |  | 1 |  |  |  |  | 1 |  |  |  |  | 1 | 1 |  |  |  |  |  | 1 |  |  |  |  |  | 1 |  |  |  | 1 |  |  |  | 1 |  |  | 1 |  |  |  | 1 |  | 1 |  |  |  | 1 |  |  | 1 |  |  |  | 1 |  |  | 1 |  |  |  | 1 |  |  |  |  | 1 |  |  |  |  | 1 |  |  |  |  | 1 |  | 1 | 1 |  |  | 1 | 1 | 1 |  |  |  |  |  | 1 |  | 1 |  | 1 |  | 1 | 1 | 1 | 1 |  |  |  | 1 | 1 |  | 1 |  |  |  | 1 |  | 1 |  |  | 1 | 1 |  |  | 1 |  |  |
| 93 | 1 | 3 | 2 | 1 |  | 1 |  |  | 1 |  |  |  | 1 |  |  |  |  | 1 |  |  |  |  | 1 |  |  |  |  | 1 |  | 1 | 1 |  |  | 1 |  | 1 |  |  |  |  | 1 |  |  | 1 |  |  |  | 1 | 1 | 1 | 1 |  |  |  | 1 |  |  | 1 |  |  |  | 1 |  | 1 |  |  | 1 | 1 |  |  | 1 |  |  |  | 1 |  |  | 1 |  |  |  | 1 |  |  |  |  | 1 |  |  |  |  | 1 |  |  |  |  | 1 |  | 1 | 1 |  |  | 1 | 1 | 1 |  |  |  |  |  | 1 |  |  |  | 1 |  | 1 | 1 | 1 | 1 |  |  |  | 1 | 1 |  | 1 |  |  |  | 1 |  | 1 |  |  | 1 | 1 |  |  | 1 |  |  |
| 94 | 1 | 2 | 1 | 2 | 1 |  |  |  |  | 1 |  |  |  |  |  | 1 |  | 1 |  |  |  |  |  | 1 |  |  | 1 |  |  | 1 |  |  |  | 1 | 1 |  |  |  |  |  |  |  | 1 |  |  |  | 1 |  | 1 |  | 1 |  |  |  |  |  | 1 | 1 |  |  |  | 1 |  | 1 |  |  |  | 1 |  |  |  |  | 1 | 1 |  |  |  | 1 |  |  |  | 1 |  |  |  |  | 1 |  |  |  |  | 1 |  |  | 1 |  |  |  | 1 |  |  |  | 1 | 1 |  |  | 1 | 1 |  |  |  |  | 1 |  |  |  |  | 1 |  | 1 |  |  |  | 1 |  |  | 1 |  |  |  | 1 |  | 1 |  |  |  | 1 |  |  | 1 |  |  |
| 95 | 1 | 1 | 1 | 1 |  | 1 |  |  | 1 |  |  |  |  |  |  | 1 |  | 1 |  |  |  |  |  | 1 |  |  |  |  | 1 |  |  |  |  |  |  |  | 1 |  |  |  |  | 1 |  |  |  |  | 1 |  | 1 |  | 1 |  |  |  | 1 |  |  | 1 |  |  |  | 1 |  | 1 |  |  |  |  | 1 |  | 1 |  |  |  | 1 |  | 1 |  |  |  |  |  |  |  | 1 |  | 1 |  |  |  | 1 |  |  |  |  |  | 1 |  |  |  |  |  | 1 | 1 |  |  |  |  |  |  | 1 |  |  |  | 1 |  | 1 | 1 |  |  |  |  |  | 1 |  |  | 1 |  |  |  | 1 |  | 1 |  |  | 1 |  |  |  | 1 |  |  |
| 96 | 1 | 3 | 3 | 1 | 1 |  |  |  | 1 |  |  |  |  |  |  |  | 1 | 1 |  |  |  |  |  | 1 |  |  |  |  | 1 | 1 | 1 |  |  | 1 | 1 |  |  |  |  |  |  |  | 1 |  |  |  | 1 |  |  |  | 1 |  |  |  | 1 |  |  | 1 |  |  |  | 1 |  | 1 |  |  |  | 1 |  |  | 1 |  |  |  | 1 |  |  | 1 |  |  |  | 1 |  |  |  |  | 1 |  |  |  |  | 1 |  |  |  | 1 | 1 |  | 1 | 1 |  |  | 1 | 1 | 1 |  |  |  |  |  | 1 |  |  |  | 1 |  | 1 | 1 | 1 | 1 |  |  |  | 1 | 1 |  | 1 |  |  |  | 1 |  | 1 |  |  | 1 | 1 |  |  | 1 |  |  |
| 97 | 1 | 4 | 4 | 3 |  | 1 |  |  | 1 |  |  |  |  |  |  | 1 |  | 1 |  |  |  |  |  | 1 |  |  |  |  | 1 |  |  | 1 |  |  | 1 |  |  |  |  |  |  | 1 |  |  |  |  | 1 |  |  | 1 | 1 |  |  |  | 1 |  |  |  | 1 |  |  | 1 |  |  |  |  |  |  |  | 1 | 1 |  |  |  | 1 |  |  | 1 |  |  |  | 1 |  |  |  |  | 1 |  |  |  |  | 1 |  |  |  | 1 |  |  | 1 | 1 | 1 |  | 1 | 1 | 1 |  |  |  |  |  | 1 |  |  |  | 1 |  | 1 | 1 | 1 | 1 |  |  |  | 1 | 1 |  | 1 |  |  |  | 1 |  | 1 |  |  | 1 | 1 |  |  | 1 |  |  |
| 98 | 1 | 2 | 2 | 1 | 1 |  |  |  | 1 |  |  |  |  |  |  |  | 1 | 1 |  |  |  |  |  | 1 |  |  |  |  | 1 | 1 | 1 |  |  | 1 | 1 |  |  |  |  |  | 1 |  |  |  |  |  | 1 |  | 1 |  | 1 |  |  |  |  |  | 1 | 1 |  |  |  | 1 |  |  |  |  |  |  |  | 1 |  |  | 1 |  | 1 |  |  | 1 |  |  |  | 1 |  |  |  |  | 1 |  |  |  |  | 1 |  |  |  | 1 |  |  | 1 | 1 |  |  | 1 | 1 | 1 |  |  |  |  |  | 1 |  |  |  | 1 |  | 1 | 1 | 1 | 1 |  |  |  | 1 | 1 |  | 1 |  |  |  | 1 |  | 1 |  |  | 1 | 1 |  |  | 1 |  |  |
| 99 | 1 | 3 | 3 | 1 |  | 1 |  |  | 1 |  |  |  |  |  |  |  | 1 | 1 |  |  |  |  |  | 1 |  |  | 1 |  |  |  | 1 |  |  | 1 | 1 |  |  |  |  |  | 1 |  |  |  |  |  | 1 |  |  |  | 1 |  |  |  | 1 |  |  |  | 1 |  |  | 1 |  | 1 |  |  |  | 1 |  |  | 1 |  |  |  | 1 |  |  | 1 |  |  |  | 1 |  |  |  |  | 1 |  |  |  |  | 1 |  |  |  | 1 |  |  | 1 | 1 |  |  | 1 | 1 | 1 |  |  |  |  |  | 1 |  |  |  | 1 |  | 1 | 1 | 1 | 1 |  |  |  | 1 | 1 |  | 1 |  |  |  | 1 |  | 1 |  |  | 1 | 1 |  |  | 1 |  |  |
| 100 | 2 | 3 | 2 | 3 |  | 1 |  |  | 1 |  |  |  |  |  |  | 1 |  |  | 1 |  |  |  |  | 1 |  |  |  |  | 1 | 1 | 1 |  |  | 1 | 1 |  |  |  |  |  | 1 |  |  | 1 |  |  |  |  | 1 |  | 1 |  |  |  | 1 |  |  | 1 |  |  |  | 1 |  |  |  |  | 1 |  |  |  | 1 |  |  |  | 1 |  |  | 1 |  |  |  | 1 |  |  |  |  | 1 |  |  |  |  | 1 |  |  |  | 1 |  |  | 1 | 1 |  |  | 1 | 1 | 1 |  |  |  |  |  | 1 |  |  |  | 1 |  | 1 | 1 | 1 | 1 |  |  |  | 1 | 1 |  | 1 |  |  |  | 1 |  | 1 |  |  | 1 | 1 |  |  | 1 |  |  |
| 101 | 2 | 3 | 2 | 1 |  | 1 |  |  | 1 |  |  |  | 1 |  |  |  |  | 1 |  |  |  |  |  | 1 |  |  |  | 1 |  | 1 | 1 |  |  | 1 | 1 |  |  |  |  |  |  | 1 |  | 1 |  |  |  |  | 1 |  | 1 |  |  |  |  | 1 |  | 1 |  |  |  | 1 |  |  |  |  |  |  |  | 1 | 1 |  |  |  | 1 |  |  | 1 |  |  |  | 1 |  |  |  |  | 1 |  |  |  |  | 1 |  |  |  | 1 |  |  | 1 | 1 |  |  | 1 | 1 | 1 |  |  |  |  |  | 1 |  |  |  | 1 |  | 1 | 1 | 1 | 1 |  |  |  | 1 | 1 |  | 1 |  |  |  | 1 |  | 1 |  |  | 1 | 1 |  |  | 1 |  |  |
| 102 | 1 | 3 | 4 | 1 |  | 1 |  |  | 1 |  |  |  |  |  |  | 1 |  | 1 |  |  |  |  |  | 1 |  |  | 1 |  |  |  | 1 |  |  | 1 | 1 |  |  |  |  |  | 1 |  |  | 1 |  |  |  | 1 | 1 |  | 1 |  |  |  | 1 |  |  | 1 |  |  |  | 1 |  |  |  |  | 1 |  |  |  | 1 |  |  |  | 1 |  |  | 1 |  |  |  | 1 |  |  |  |  | 1 |  |  |  |  | 1 |  |  |  | 1 |  |  | 1 | 1 |  |  | 1 | 1 | 1 |  |  |  |  |  | 1 |  |  |  | 1 |  | 1 | 1 |  | 1 |  |  |  | 1 |  |  | 1 |  |  |  | 1 |  | 1 |  |  | 1 | 1 |  |  | 1 |  |  |
| 103 | 1 | 4 | 2 | 6 |  | 1 |  |  | 1 |  |  |  |  |  |  | 1 |  | 1 |  |  |  |  |  | 1 |  |  | 1 |  |  | 1 | 1 |  |  | 1 |  |  |  |  |  |  |  | 1 |  | 1 |  |  |  | 1 | 1 | 1 | 1 |  |  |  | 1 |  |  | 1 |  |  |  | 1 |  | 1 |  |  | 1 |  |  |  | 1 |  |  |  | 1 |  |  | 1 |  |  |  |  |  |  | 1 |  | 1 |  |  |  |  | 1 |  |  |  | 1 |  |  | 1 | 1 |  |  | 1 |  |  |  |  |  |  |  | 1 |  | 1 |  |  |  | 1 | 1 | 1 | 1 |  |  |  | 1 |  |  | 1 |  |  |  | 1 |  | 1 |  |  | 1 |  |  |  | 1 |  |  |
| 104 | 1 | 2 | 4 | 2 |  | 1 |  | 1 |  |  |  |  |  |  |  |  | 1 | 1 |  |  |  |  |  | 1 |  |  |  | 1 |  |  |  |  |  | 1 | 1 |  |  |  |  |  | 1 |  |  |  | 1 |  |  |  | 1 | 1 | 1 |  |  |  | 1 |  |  | 1 |  |  |  | 1 |  | 1 |  |  |  |  |  | 1 | 1 |  |  |  | 1 |  |  | 1 |  |  |  | 1 |  |  |  |  | 1 |  |  |  |  | 1 |  |  |  |  | 1 |  | 1 | 1 |  |  | 1 | 1 | 1 |  |  |  |  |  | 1 |  |  |  | 1 |  | 1 | 1 | 1 | 1 |  |  |  | 1 |  |  | 1 |  |  |  | 1 |  |  |  |  | 1 | 1 |  |  | 1 |  |  |
| 105 | 2 | 2 | 2 | 2 |  |  | 1 |  | 1 |  |  |  |  |  |  |  | 1 |  | 1 |  |  |  | 1 |  |  |  |  |  | 1 |  |  |  |  |  |  |  | 1 |  |  |  | 1 |  |  | 1 |  |  |  |  | 1 |  | 1 |  |  |  | 1 |  |  | 1 |  |  |  | 1 |  |  |  |  |  | 1 |  |  | 1 |  |  |  | 1 |  |  | 1 |  |  |  | 1 |  |  |  |  | 1 |  |  |  |  | 1 |  |  |  | 1 | 1 |  | 1 | 1 |  |  | 1 | 1 | 1 |  |  |  |  |  | 1 |  |  |  | 1 |  | 1 | 1 | 1 | 1 |  |  |  | 1 | 1 |  | 1 |  |  |  | 1 |  | 1 |  |  | 1 | 1 |  |  | 1 |  |  |
| 106 | 1 | 1 | 1 | 1 |  | 1 |  |  | 1 |  |  |  |  |  |  | 1 |  | 1 |  |  |  |  |  | 1 |  |  |  |  | 1 |  |  |  |  | 1 | 1 |  |  |  |  |  | 1 |  |  | 1 |  |  |  | 1 | 1 |  | 1 |  |  |  | 1 |  |  | 1 |  |  |  | 1 |  |  |  |  | 1 |  |  |  | 1 |  |  |  | 1 |  |  | 1 |  |  |  | 1 |  |  |  |  | 1 |  |  |  |  | 1 |  |  |  |  |  | 1 | 1 | 1 |  |  | 1 | 1 | 1 |  |  |  |  | 1 |  |  |  |  | 1 |  | 1 |  | 1 | 1 |  |  |  | 1 | 1 |  | 1 |  |  |  | 1 |  | 1 |  |  | 1 |  |  |  | 1 |  |  |
| 107 | 1 | 3 | 2 | 1 |  | 1 |  |  | 1 |  |  |  |  |  |  |  | 1 | 1 |  |  |  |  | 1 |  |  |  |  |  | 1 |  |  |  |  | 1 |  |  |  |  |  |  |  |  | 1 |  |  |  | 1 |  | 1 |  |  |  |  |  |  | 1 |  | 1 |  |  |  |  |  |  | 1 |  | 1 |  |  |  | 1 |  |  |  | 1 |  |  | 1 |  |  |  | 1 |  |  |  |  | 1 |  |  |  |  | 1 |  |  |  |  |  | 1 |  | 1 |  |  | 1 | 1 |  |  |  |  |  |  | 1 |  |  |  | 1 |  | 1 | 1 |  | 1 |  |  |  |  | 1 |  | 1 |  |  |  | 1 |  | 1 |  |  | 1 |  |  |  | 1 |  |  |
| 108 | 1 | 3 | 1 | 1 |  | 1 |  | 1 |  |  |  |  |  |  |  |  | 1 |  | 1 |  |  |  |  | 1 |  |  |  |  | 1 |  |  |  |  | 1 | 1 |  |  |  |  |  | 1 |  |  | 1 |  |  |  |  | 1 |  |  |  |  |  | 1 |  |  | 1 |  |  |  | 1 |  | 1 |  |  | 1 | 1 |  |  | 1 |  |  |  | 1 |  |  | 1 |  |  |  | 1 |  |  |  |  | 1 |  |  |  |  | 1 |  |  |  | 1 | 1 |  | 1 | 1 |  |  | 1 | 1 | 1 |  |  |  |  |  | 1 |  |  |  | 1 |  | 1 | 1 | 1 | 1 |  |  |  | 1 | 1 |  | 1 |  |  |  | 1 |  | 1 |  |  | 1 | 1 |  |  | 1 |  |  |
| 109 | 1 | 3 | 1 | 3 |  |  | 1 |  | 1 |  |  |  |  |  |  | 1 |  |  | 1 |  |  |  |  |  | 1 |  |  |  | 1 | 1 |  |  |  | 1 | 1 |  |  |  |  |  |  |  | 1 |  |  |  | 1 |  |  |  | 1 |  |  |  |  | 1 |  | 1 |  |  |  |  |  |  | 1 |  |  |  |  | 1 | 1 |  |  |  | 1 |  |  | 1 |  |  |  |  |  |  |  | 1 |  | 1 |  |  |  | 1 |  |  | 1 |  |  |  | 1 |  |  |  | 1 | 1 | 1 |  |  |  |  |  |  | 1 |  |  |  | 1 |  | 1 | 1 | 1 |  |  |  |  | 1 |  | 1 |  |  |  | 1 |  |  |  |  |  |  |  | 1 | 1 |  |  |
| 110 | 2 | 4 | 1 | 1 |  | 1 |  |  | 1 |  |  |  |  |  |  |  | 1 | 1 |  |  |  |  | 1 |  |  |  | 1 |  |  | 1 |  |  |  | 1 | 1 | 1 |  |  |  |  |  |  | 1 |  |  |  | 1 |  | 1 |  |  |  |  |  | 1 |  |  | 1 |  |  |  | 1 |  | 1 |  |  |  |  |  | 1 | 1 |  |  |  | 1 |  |  | 1 |  |  |  | 1 |  |  |  |  | 1 |  |  |  |  | 1 |  |  |  | 1 | 1 |  | 1 | 1 |  |  | 1 | 1 | 1 |  |  |  |  | 1 |  |  |  |  | 1 |  | 1 | 1 |  | 1 |  |  |  | 1 |  |  | 1 |  |  |  | 1 |  | 1 |  |  | 1 |  |  |  | 1 |  |  |
| 111 | 1 | 3 | 4 | 1 |  | 1 |  |  | 1 |  |  |  |  |  |  | 1 |  | 1 |  |  |  |  |  | 1 |  |  | 1 |  |  |  |  |  |  | 1 | 1 |  |  |  |  |  |  |  | 1 | 1 |  |  |  |  | 1 |  | 1 |  |  |  | 1 |  |  | 1 |  |  |  | 1 |  | 1 |  |  | 1 |  |  |  | 1 |  |  |  | 1 |  |  | 1 |  |  |  | 1 |  |  |  |  | 1 |  |  |  |  | 1 |  |  |  | 1 | 1 |  | 1 | 1 |  |  | 1 | 1 | 1 |  |  |  |  |  | 1 |  |  |  | 1 |  | 1 | 1 | 1 | 1 |  |  |  | 1 | 1 |  | 1 |  |  |  | 1 |  | 1 |  |  | 1 |  |  |  | 1 |  |  |
| 112 | 1 | 3 | 2 | 1 | 1 |  |  |  | 1 |  |  |  |  |  |  | 1 |  |  | 1 |  |  |  |  | 1 |  |  | 1 |  |  |  | 1 |  |  | 1 | 1 | 1 |  |  |  |  | 1 |  |  | 1 |  |  |  | 1 | 1 | 1 | 1 |  |  |  | 1 |  |  | 1 |  |  |  | 1 |  | 1 |  |  | 1 | 1 |  |  | 1 |  |  |  | 1 |  |  | 1 |  |  |  | 1 |  |  |  |  | 1 |  |  |  |  | 1 |  |  |  | 1 | 1 |  | 1 | 1 |  |  | 1 | 1 | 1 |  |  |  |  |  | 1 |  | 1 |  |  |  | 1 | 1 | 1 | 1 |  |  |  | 1 | 1 |  | 1 |  |  |  | 1 |  | 1 |  |  | 1 | 1 |  |  | 1 |  |  |
| 113 | 2 | 2 | 2 | 1 | 1 |  |  | 1 |  |  |  |  |  |  |  | 1 |  |  | 1 |  |  |  |  | 1 |  |  |  | 1 |  |  | 1 |  |  | 1 |  | 1 |  |  |  |  | 1 |  |  | 1 |  |  |  |  | 1 |  | 1 |  |  |  | 1 |  |  | 1 |  |  |  | 1 |  |  |  |  | 1 | 1 |  |  | 1 |  |  |  | 1 |  |  | 1 |  |  |  | 1 |  |  |  |  | 1 |  |  |  |  | 1 |  |  |  | 1 | 1 |  | 1 | 1 |  |  | 1 | 1 | 1 |  |  |  |  |  | 1 |  |  |  | 1 |  | 1 | 1 | 1 | 1 |  |  |  | 1 | 1 |  | 1 |  |  |  | 1 |  | 1 |  |  | 1 | 1 |  |  | 1 |  |  |
| 114 | 2 | 3 | 4 | 1 |  |  | 1 |  |  |  |  | 1 |  |  |  |  | 1 | 1 |  |  |  |  | 1 |  |  |  |  |  | 1 |  |  |  |  |  |  |  | 1 |  |  |  |  |  | 1 |  |  |  | 1 |  |  |  | 1 |  |  |  |  |  | 1 |  |  | 1 |  |  |  |  | 1 |  |  |  |  | 1 | 1 |  |  | 1 |  |  | 1 |  |  |  |  |  |  |  |  | 1 |  |  |  | 1 |  | 1 |  |  |  |  |  | 1 |  |  |  |  |  | 1 |  |  |  |  |  |  |  | 1 |  |  |  | 1 |  |  |  |  | 1 |  |  | 1 |  |  | 1 |  |  |  | 1 |  |  |  |  |  | 1 |  |  | 1 |  |  |
| 115 | 2 | 4 | 1 | 1 |  | 1 |  |  | 1 |  |  |  |  |  |  | 1 |  |  | 1 |  |  |  | 1 |  |  | 1 |  |  |  |  |  |  |  |  |  |  | 1 | 1 |  |  |  |  |  |  |  |  | 1 |  |  |  |  | 1 | 1 |  |  |  |  |  | 1 |  |  |  | 1 |  |  |  | 1 |  |  |  |  |  | 1 |  | 1 |  |  | 1 |  |  |  |  |  |  | 1 |  | 1 |  |  |  |  | 1 |  |  |  |  |  | 1 |  | 1 |  |  | 1 | 1 |  |  |  |  |  |  |  | 1 |  |  | 1 |  |  |  |  | 1 |  |  |  | 1 |  |  | 1 |  |  |  | 1 |  | 1 |  |  |  | 1 |  |  | 1 |  |  |
| 116 | 2 | 4 | 2 | 2 |  | 1 |  |  |  | 1 |  |  |  |  |  |  | 1 |  | 1 |  |  |  |  | 1 |  |  |  |  | 1 |  |  |  |  |  | 1 | 1 |  |  |  |  |  | 1 |  |  |  |  | 1 |  | 1 |  | 1 |  |  |  | 1 |  |  |  |  | 1 |  | 1 |  |  |  |  | 1 | 1 |  |  | 1 |  |  |  | 1 |  | 1 |  | 1 |  |  |  |  |  |  | 1 |  | 1 |  |  |  | 1 |  |  |  |  |  | 1 |  |  |  |  |  | 1 | 1 |  |  |  |  |  | 1 |  |  |  |  | 1 | 1 | 1 | 1 | 1 |  |  |  | 1 |  |  | 1 |  |  |  | 1 |  |  |  |  |  | 1 |  |  | 1 |  |  |
| 117 | 2 | 2 | 1 | 1 |  | 1 |  |  | 1 |  |  |  |  |  |  |  | 1 | 1 |  |  |  |  |  | 1 |  |  |  |  | 1 |  | 1 |  |  | 1 | 1 |  |  |  |  |  | 1 |  |  |  |  |  | 1 | 1 | 1 |  | 1 |  |  |  |  |  | 1 |  | 1 |  |  | 1 |  | 1 |  |  |  | 1 |  |  | 1 |  |  | 1 |  |  |  | 1 |  |  |  | 1 |  |  |  |  | 1 |  |  |  |  | 1 |  |  |  |  |  | 1 | 1 | 1 |  |  | 1 | 1 | 1 |  |  |  |  | 1 |  |  |  |  |  | 1 |  | 1 |  | 1 |  |  |  |  |  | 1 | 1 |  |  |  | 1 |  |  |  |  |  | 1 |  | 1 | 1 |  |  |
| 118 | 1 | 4 | 2 | 1 |  | 1 |  |  | 1 |  |  |  |  |  |  | 1 |  | 1 |  |  |  |  | 1 |  |  |  |  |  | 1 |  | 1 |  |  | 1 | 1 |  |  |  |  |  |  | 1 |  | 1 |  |  |  | 1 | 1 |  | 1 |  |  |  | 1 |  |  | 1 |  |  |  | 1 |  | 1 |  |  | 1 | 1 |  |  | 1 |  |  |  | 1 |  |  | 1 |  |  |  | 1 |  |  |  |  | 1 |  |  |  |  | 1 |  |  |  |  |  | 1 | 1 | 1 |  |  | 1 | 1 |  |  |  |  |  |  | 1 |  |  |  | 1 |  | 1 | 1 | 1 | 1 |  |  |  | 1 |  |  | 1 |  |  |  |  |  | 1 |  |  | 1 | 1 |  |  | 1 |  |  |
| 119 | 1 | 4 | 2 | 1 |  | 1 |  | 1 |  |  |  |  |  |  |  |  | 1 | 1 |  |  |  |  |  | 1 |  |  |  |  | 1 | 1 | 1 | 1 |  | 1 | 1 | 1 |  |  |  |  | 1 |  |  |  |  |  | 1 |  | 1 |  |  |  |  |  | 1 |  |  | 1 |  |  |  | 1 |  |  |  |  | 1 |  |  |  | 1 |  |  |  | 1 |  |  | 1 |  |  |  |  |  |  | 1 |  | 1 |  |  |  |  |  | 1 |  |  |  |  | 1 | 1 | 1 |  |  | 1 | 1 | 1 |  |  |  |  | 1 |  |  |  |  |  | 1 |  | 1 |  | 1 |  |  |  | 1 |  |  | 1 |  |  |  | 1 |  |  |  |  |  |  |  | 1 | 1 |  |  |
| 120 | 2 | 2 | 4 | 6 | 1 | 1 |  |  |  |  |  | 1 | 1 |  |  |  |  |  | 1 |  |  |  |  | 1 |  | 1 |  |  |  | 1 | 1 |  |  | 1 | 1 | 1 |  |  |  |  |  | 1 |  |  |  | 1 |  | 1 | 1 | 1 | 1 |  |  |  | 1 | 1 |  | 1 |  |  |  | 1 |  | 1 |  |  | 1 | 1 |  |  | 1 |  |  |  | 1 |  |  | 1 |  |  |  |  |  |  | 1 |  | 1 |  |  |  |  | 1 |  |  |  | 1 |  |  | 1 | 1 |  |  | 1 | 1 | 1 |  |  |  |  |  | 1 |  | 1 |  |  |  |  | 1 | 1 | 1 |  |  |  |  | 1 |  | 1 |  |  |  | 1 |  |  |  |  |  | 1 |  |  | 1 |  |  |
| 121 | 1 | 4 | 2 | 3 |  | 1 |  |  | 1 |  |  |  |  |  |  |  | 1 | 1 |  |  |  |  | 1 |  |  |  |  |  | 1 | 1 | 1 |  |  | 1 | 1 | 1 |  |  |  |  |  | 1 |  |  |  |  | 1 |  |  |  | 1 |  |  |  | 1 |  |  |  | 1 |  |  | 1 |  | 1 |  |  |  | 1 |  |  | 1 |  |  |  | 1 |  |  | 1 |  |  |  | 1 |  |  |  |  | 1 |  |  |  |  | 1 |  |  |  |  |  | 1 | 1 | 1 |  |  | 1 | 1 |  |  |  |  |  |  | 1 |  | 1 |  |  |  |  |  | 1 | 1 |  |  |  | 1 |  |  | 1 |  |  |  | 1 |  | 1 |  |  |  | 1 |  |  | 1 |  |  |
| 122 | 1 | 3 | 1 | 1 |  | 1 |  | 1 |  |  |  |  |  | 1 |  |  |  | 1 |  |  |  |  |  | 1 |  |  |  |  | 1 |  | 1 |  |  | 1 | 1 |  |  |  |  |  |  | 1 |  | 1 |  |  |  | 1 | 1 |  | 1 |  |  |  | 1 |  |  | 1 |  |  |  | 1 |  | 1 |  |  |  | 1 |  |  | 1 | 1 |  |  | 1 |  |  | 1 |  |  |  | 1 |  |  |  |  | 1 |  |  |  |  | 1 |  |  |  | 1 |  |  | 1 | 1 |  |  | 1 | 1 |  |  |  |  |  |  | 1 |  | 1 |  |  |  | 1 | 1 | 1 | 1 |  |  |  | 1 |  |  | 1 |  |  |  | 1 |  | 1 |  |  |  | 1 |  |  | 1 |  |  |
| 123 | 2 | 3 | 2 | 1 |  | 1 |  |  | 1 |  |  |  |  |  |  | 1 |  |  |  | 1 |  |  | 1 |  |  |  | 1 |  |  | 1 | 1 |  |  | 1 | 1 |  |  |  |  |  |  | 1 |  |  | 1 |  |  |  | 1 |  | 1 |  |  |  | 1 |  |  | 1 |  |  |  | 1 |  | 1 |  |  | 1 | 1 |  |  | 1 |  |  |  | 1 |  |  | 1 |  |  |  | 1 |  |  |  |  | 1 |  |  |  |  | 1 |  |  |  | 1 |  |  | 1 | 1 |  |  | 1 | 1 |  |  |  |  |  |  | 1 |  | 1 |  |  |  |  | 1 | 1 | 1 |  |  |  | 1 | 1 |  | 1 |  |  |  | 1 |  | 1 |  |  | 1 | 1 |  |  | 1 |  |  |
| 124 | 2 | 4 | 2 | 4 |  | 1 |  | 1 |  |  |  |  |  |  |  |  | 1 |  |  |  | 1 |  |  | 1 |  |  |  |  | 1 |  |  |  |  | 1 | 1 | 1 |  |  |  |  |  | 1 |  |  |  |  | 1 |  | 1 |  | 1 |  |  |  | 1 |  |  |  | 1 |  |  | 1 |  | 1 |  |  |  | 1 |  |  | 1 |  |  |  | 1 |  | 1 |  |  |  |  |  | 1 |  |  |  |  | 1 |  |  |  | 1 |  |  |  |  |  | 1 |  |  |  |  | 1 | 1 |  |  |  |  |  |  | 1 |  |  |  | 1 |  | 1 | 1 | 1 | 1 |  |  |  | 1 | 1 |  | 1 |  |  |  | 1 |  |  |  |  |  | 1 |  |  | 1 |  |  |
| 125 | 2 | 4 | 1 | 1 |  | 1 |  |  | 1 |  |  |  | 1 |  |  |  |  | 1 |  |  |  |  | 1 |  |  |  |  | 1 |  | 1 |  |  |  |  |  |  |  |  |  |  | 1 |  |  |  |  | 1 |  |  | 1 | 1 | 1 |  |  |  |  | 1 |  | 1 |  |  |  | 1 |  |  |  |  | 1 |  |  |  | 1 |  |  |  | 1 |  |  | 1 |  |  |  | 1 |  |  |  |  | 1 |  |  |  |  | 1 |  |  |  |  | 1 |  | 1 |  |  |  |  |  |  |  |  |  |  | 1 |  |  |  | 1 |  |  | 1 | 1 | 1 | 1 |  |  |  |  | 1 |  | 1 |  |  |  | 1 |  |  |  |  | 1 | 1 |  |  | 1 |  |  |
| 126 | 1 | 4 | 1 | 1 |  | 1 |  |  | 1 |  |  |  |  |  |  |  | 1 | 1 |  |  |  |  |  | 1 |  |  |  |  | 1 |  | 1 |  |  |  | 1 |  |  |  |  |  | 1 |  |  |  |  |  | 1 |  | 1 |  | 1 |  |  |  |  |  | 1 |  |  | 1 |  | 1 |  | 1 |  |  |  |  |  | 1 | 1 |  |  |  | 1 |  |  | 1 |  |  |  | 1 |  |  |  |  | 1 |  |  |  |  | 1 |  |  |  |  |  | 1 | 1 | 1 |  |  |  | 1 |  |  |  |  |  | 1 |  |  | 1 |  |  |  |  | 1 | 1 | 1 |  |  |  | 1 |  |  | 1 |  |  |  | 1 |  | 1 |  |  |  | 1 |  |  | 1 |  |  |
| 127 | 2 | 3 | 3 | 1 |  |  | 1 |  | 1 |  |  |  |  |  |  | 1 |  |  |  | 1 |  |  | 1 |  |  |  | 1 |  |  | 1 | 1 |  |  | 1 | 1 |  |  |  |  |  |  | 1 |  |  |  |  | 1 |  |  |  | 1 |  |  |  |  | 1 |  |  | 1 |  |  | 1 |  | 1 |  |  | 1 | 1 |  |  | 1 |  |  |  | 1 |  |  | 1 |  |  |  | 1 |  |  |  |  | 1 |  |  |  |  | 1 |  |  |  | 1 |  |  | 1 | 1 |  |  | 1 | 1 |  |  |  |  |  |  | 1 |  |  |  | 1 |  |  | 1 | 1 | 1 |  |  |  | 1 |  |  | 1 |  |  |  | 1 |  | 1 |  |  |  | 1 |  |  | 1 |  |  |
| 128 | 2 | 3 | 4 | 1 |  | 1 |  |  | 1 |  |  |  |  |  |  | 1 |  | 1 |  |  |  |  | 1 |  |  |  |  |  | 1 | 1 | 1 |  |  | 1 | 1 | 1 |  |  |  |  |  | 1 |  |  |  |  | 1 |  |  |  | 1 |  |  |  | 1 |  |  |  | 1 |  |  | 1 |  | 1 |  |  |  | 1 |  |  | 1 |  |  |  | 1 |  |  | 1 |  |  |  | 1 |  |  |  |  | 1 |  |  |  |  | 1 |  |  |  |  |  | 1 | 1 | 1 |  |  | 1 |  |  |  |  |  |  |  | 1 |  |  |  | 1 |  |  |  | 1 | 1 |  |  |  | 1 |  |  | 1 |  |  |  | 1 |  | 1 |  |  |  | 1 |  |  | 1 |  |  |
| 129 | 1 | 1 | 1 | 1 | 1 |  |  |  | 1 |  |  |  | 1 |  |  |  |  | 1 |  |  |  |  | 1 |  |  |  |  |  | 1 |  | 1 |  |  | 1 | 1 | 1 |  |  | 1 |  |  |  |  | 1 |  |  |  | 1 | 1 |  | 1 |  |  |  |  | 1 |  |  | 1 |  |  | 1 |  |  |  |  |  | 1 |  |  | 1 |  |  |  | 1 |  |  | 1 |  |  |  | 1 |  |  |  |  | 1 |  |  |  |  | 1 |  |  | 1 |  |  |  |  | 1 |  |  | 1 | 1 | 1 |  |  |  |  |  | 1 |  |  |  | 1 |  | 1 | 1 |  | 1 |  |  |  | 1 |  |  | 1 |  |  |  | 1 |  | 1 |  |  | 1 | 1 |  |  | 1 |  |  |
| 130 | 1 | 4 | 2 | 3 |  |  | 1 |  |  |  |  | 1 |  |  |  |  | 1 | 1 |  |  |  |  | 1 |  |  |  |  |  | 1 | 1 |  |  |  | 1 | 1 |  |  |  |  |  |  |  | 1 |  |  |  | 1 |  | 1 |  | 1 |  |  |  |  |  | 1 |  |  | 1 |  | 1 |  | 1 |  |  |  |  |  | 1 | 1 |  |  |  | 1 |  |  | 1 |  |  |  | 1 |  |  | 1 |  | 1 |  |  |  |  | 1 |  |  |  | 1 |  |  | 1 | 1 |  |  | 1 | 1 | 1 |  |  |  |  | 1 |  |  | 1 |  |  |  | 1 | 1 | 1 | 1 |  |  |  | 1 |  |  | 1 |  |  |  | 1 |  | 1 |  |  | 1 | 1 |  |  | 1 |  |  |
| 131 | 2 | 4 | 3 | 1 |  | 1 |  |  | 1 |  |  |  |  |  |  | 1 |  | 1 |  |  |  |  |  | 1 |  |  |  |  | 1 |  | 1 |  |  | 1 | 1 |  |  |  |  |  | 1 |  |  | 1 |  |  |  |  | 1 |  | 1 |  |  |  | 1 | 1 |  | 1 |  |  |  | 1 |  | 1 |  |  | 1 |  |  |  | 1 |  |  |  | 1 |  |  | 1 |  |  |  | 1 |  |  |  |  | 1 |  |  |  |  | 1 |  |  |  | 1 |  |  | 1 | 1 | 1 |  | 1 | 1 | 1 |  |  |  |  |  | 1 |  | 1 |  |  |  | 1 | 1 | 1 | 1 |  |  |  | 1 | 1 |  | 1 |  |  |  | 1 |  | 1 |  |  | 1 | 1 |  |  | 1 |  |  |
| 132 | 2 | 3 | 2 | 1 |  | 1 |  |  | 1 |  |  |  | 1 |  |  |  |  | 1 |  |  |  |  | 1 |  |  |  |  |  | 1 | 1 | 1 |  |  | 1 | 1 |  |  |  |  |  |  | 1 |  | 1 |  |  |  |  |  |  | 1 |  |  |  | 1 |  |  | 1 |  |  |  | 1 |  |  |  |  |  | 1 |  |  | 1 |  |  |  | 1 |  |  | 1 |  |  |  | 1 |  |  |  |  | 1 |  |  |  |  | 1 |  |  |  | 1 |  |  | 1 | 1 |  |  | 1 | 1 |  |  |  |  |  |  | 1 |  | 1 |  |  |  |  | 1 | 1 | 1 |  |  |  | 1 | 1 |  | 1 |  |  |  | 1 |  | 1 |  |  | 1 | 1 |  |  | 1 |  |  |
| 133 | 1 | 3 | 4 | 1 |  |  | 1 |  |  | 1 |  |  |  |  |  |  | 1 |  | 1 |  |  |  | 1 |  |  |  |  |  | 1 | 1 | 1 |  |  | 1 | 1 |  |  |  |  |  | 1 |  |  |  |  |  | 1 | 1 |  |  | 1 |  |  |  | 1 |  |  | 1 |  |  |  | 1 |  |  |  |  |  | 1 |  |  | 1 |  |  |  | 1 |  |  | 1 |  |  |  | 1 |  |  | 1 |  | 1 |  |  |  |  | 1 |  |  |  | 1 |  |  | 1 | 1 |  |  | 1 | 1 | 1 |  |  |  |  | 1 |  |  |  |  | 1 |  | 1 | 1 | 1 | 1 |  |  |  | 1 |  |  | 1 |  |  |  | 1 |  | 1 |  |  | 1 | 1 |  |  | 1 |  |  |
| 134 | 1 | 2 | 2 | 1 | 1 |  |  |  |  | 1 |  |  | 1 |  |  |  |  |  | 1 |  |  |  | 1 |  |  |  |  |  | 1 |  | 1 |  |  |  |  |  |  |  |  |  |  | 1 |  |  |  | 1 |  |  |  |  | 1 |  |  |  |  | 1 |  | 1 |  |  |  | 1 |  |  |  |  | 1 |  |  |  | 1 |  |  | 1 |  |  |  | 1 |  |  |  | 1 |  |  |  |  |  | 1 |  |  |  | 1 |  |  | 1 |  |  |  | 1 | 1 |  |  | 1 |  |  |  |  |  |  |  | 1 |  |  |  | 1 |  |  | 1 |  | 1 |  |  |  | 1 |  |  | 1 |  |  |  | 1 |  | 1 |  |  | 1 | 1 |  |  | 1 |  |  |
| 135 | 1 | 3 | 2 | 1 |  | 1 |  | 1 |  |  |  |  |  |  |  | 1 |  | 1 |  |  |  |  |  | 1 |  |  | 1 |  |  |  |  |  |  | 1 | 1 |  |  |  |  | 1 |  |  |  | 1 |  |  |  |  | 1 |  | 1 |  |  |  |  | 1 |  | 1 |  |  |  | 1 |  |  |  |  |  | 1 |  |  | 1 |  |  |  | 1 |  |  | 1 |  |  |  | 1 |  |  |  |  | 1 |  |  |  |  | 1 |  |  |  | 1 |  |  |  |  |  |  | 1 | 1 |  |  |  |  |  | 1 |  |  |  |  | 1 |  |  | 1 |  | 1 |  |  |  | 1 |  |  | 1 |  |  |  | 1 |  |  |  |  |  | 1 |  |  | 1 |  |  |
| 136 | 1 | 3 | 1 | 2 | 1 |  |  | 1 |  |  |  |  |  |  |  | 1 |  | 1 |  |  |  |  | 1 |  |  |  |  |  | 1 |  |  | 1 |  |  | 1 |  |  |  |  |  |  |  | 1 |  |  |  | 1 | 1 |  |  | 1 |  |  |  |  |  | 1 |  | 1 |  |  | 1 |  | 1 |  |  | 1 |  |  |  | 1 |  |  |  | 1 |  |  | 1 |  |  |  |  |  |  | 1 |  | 1 |  |  |  |  | 1 |  |  |  | 1 |  |  | 1 | 1 |  |  | 1 | 1 |  |  |  |  |  | 1 |  |  |  |  | 1 |  | 1 | 1 |  | 1 |  |  |  | 1 |  |  | 1 |  |  |  | 1 |  |  |  |  | 1 | 1 |  |  | 1 |  |  |
| 137 | 1 | 3 | 1 | 1 |  |  | 1 |  | 1 |  |  |  |  |  |  | 1 |  | 1 |  |  |  |  | 1 |  |  | 1 |  |  |  |  |  |  |  | 1 | 1 |  |  |  |  |  | 1 |  |  |  | 1 |  |  |  |  |  | 1 |  |  |  | 1 |  |  |  |  | 1 |  | 1 |  | 1 |  |  | 1 |  |  |  | 1 |  |  | 1 |  |  |  | 1 |  |  |  | 1 |  |  |  |  | 1 |  |  |  |  | 1 |  |  |  | 1 | 1 |  | 1 | 1 |  |  | 1 | 1 | 1 |  |  |  |  | 1 | 1 |  |  |  | 1 |  | 1 | 1 | 1 | 1 |  |  |  | 1 |  |  | 1 |  |  |  | 1 |  | 1 |  |  | 1 | 1 |  |  | 1 |  |  |
| 138 | 1 | 3 | 2 | 1 |  |  | 1 | 1 |  |  |  |  |  |  |  |  | 1 | 1 |  |  |  |  |  | 1 |  |  |  |  | 1 |  | 1 |  |  | 1 | 1 |  |  |  |  |  |  |  | 1 |  |  |  | 1 | 1 | 1 |  | 1 |  |  |  | 1 | 1 |  |  |  | 1 |  | 1 |  | 1 |  |  |  |  |  | 1 | 1 |  |  |  | 1 |  |  | 1 |  |  |  | 1 |  |  | 1 |  | 1 |  |  |  |  | 1 |  |  |  | 1 |  |  | 1 | 1 |  |  | 1 | 1 | 1 |  |  |  |  | 1 |  |  | 1 |  |  |  | 1 | 1 | 1 | 1 |  |  |  | 1 |  |  | 1 |  |  |  | 1 |  | 1 |  |  | 1 | 1 |  |  | 1 |  |  |
| 139 | 1 | 3 | 1 | 5 |  |  | 1 |  |  | 1 |  |  |  |  |  |  | 1 | 1 |  |  |  |  | 1 |  |  |  |  |  | 1 |  |  |  |  |  |  |  | 1 |  |  |  |  |  | 1 |  |  |  | 1 |  |  |  |  | 1 |  |  |  |  | 1 |  |  | 1 |  |  |  |  | 1 |  |  |  |  | 1 |  |  | 1 |  | 1 |  |  | 1 |  |  |  | 1 |  |  | 1 |  | 1 |  |  |  |  | 1 |  |  |  | 1 |  |  | 1 | 1 |  |  | 1 | 1 | 1 |  |  |  |  | 1 |  |  |  |  | 1 |  | 1 | 1 | 1 | 1 |  |  |  | 1 |  |  | 1 |  |  |  | 1 |  | 1 |  |  | 1 | 1 |  |  | 1 |  |  |
| 140 | 1 | 4 | 4 | 3 |  | 1 |  |  | 1 |  |  |  |  |  |  | 1 |  | 1 |  |  |  |  | 1 |  |  |  |  |  | 1 | 1 | 1 |  |  |  | 1 | 1 |  |  |  |  |  | 1 |  | 1 |  |  |  | 1 |  |  | 1 |  |  |  | 1 | 1 |  | 1 |  |  |  | 1 |  | 1 |  |  | 1 |  |  |  | 1 |  |  |  | 1 |  |  | 1 |  |  |  | 1 |  |  | 1 |  | 1 |  |  |  |  | 1 |  |  |  | 1 | 1 |  | 1 | 1 |  |  | 1 | 1 | 1 |  |  |  |  | 1 | 1 |  |  |  | 1 |  | 1 | 1 | 1 | 1 |  |  |  | 1 | 1 |  | 1 |  |  |  | 1 |  | 1 |  |  | 1 | 1 |  |  | 1 |  |  |
| 141 | 2 | 3 | 3 | 1 |  | 1 |  |  | 1 |  |  |  |  |  |  | 1 |  | 1 |  |  |  |  |  | 1 |  |  |  |  | 1 |  |  |  |  |  | 1 |  |  |  |  |  |  | 1 |  |  | 1 |  |  |  |  |  | 1 |  |  |  | 1 |  |  |  | 1 |  |  | 1 |  | 1 |  |  | 1 | 1 |  |  | 1 |  |  |  | 1 |  |  | 1 |  |  |  | 1 |  |  | 1 |  | 1 |  |  |  |  | 1 |  |  |  | 1 |  |  | 1 | 1 |  |  | 1 | 1 | 1 |  |  |  |  | 1 |  |  | 1 |  |  |  |  | 1 |  | 1 |  |  |  | 1 |  |  |  | 1 |  |  | 1 |  | 1 |  |  | 1 | 1 |  |  | 1 |  |  |
| 142 | 2 | 1 | 1 | 2 |  | 1 |  |  | 1 |  |  |  |  |  |  |  | 1 |  | 1 |  |  |  |  | 1 |  |  |  |  | 1 | 1 | 1 |  |  | 1 | 1 |  |  |  |  |  | 1 | 1 |  |  |  |  | 1 |  | 1 |  | 1 |  |  |  | 1 |  |  |  | 1 |  |  | 1 |  | 1 |  |  | 1 | 1 |  |  | 1 |  |  |  | 1 |  |  | 1 |  |  |  | 1 |  |  | 1 |  | 1 |  |  |  |  | 1 |  |  |  | 1 |  |  | 1 | 1 |  |  | 1 | 1 | 1 |  |  |  |  | 1 | 1 |  |  |  | 1 |  | 1 | 1 | 1 | 1 |  |  |  | 1 | 1 |  | 1 |  |  |  | 1 |  | 1 |  |  | 1 | 1 |  |  | 1 |  |  |
| 143 | 2 | 3 | 2 | 1 |  | 1 |  |  | 1 |  |  |  |  |  |  | 1 |  | 1 |  |  |  |  |  | 1 |  |  | 1 |  |  | 1 | 1 |  |  | 1 | 1 | 1 |  |  |  |  | 1 |  |  |  | 1 |  |  |  | 1 |  | 1 |  |  |  | 1 |  |  | 1 |  |  |  | 1 |  | 1 |  |  |  | 1 |  |  | 1 |  |  |  | 1 |  |  | 1 |  |  |  | 1 |  |  |  |  | 1 |  |  |  |  |  | 1 |  |  | 1 |  |  | 1 | 1 |  |  | 1 | 1 | 1 |  |  |  |  |  | 1 |  | 1 |  |  |  | 1 | 1 | 1 | 1 |  |  |  | 1 | 1 |  | 1 |  |  |  | 1 |  | 1 |  |  | 1 | 1 |  |  | 1 |  |  |
| 144 | 1 | 3 | 2 | 1 |  | 1 |  |  | 1 |  |  |  |  |  |  | 1 |  | 1 |  |  |  |  | 1 |  |  |  |  | 1 |  | 1 | 1 |  |  | 1 | 1 | 1 |  |  |  |  | 1 |  |  | 1 |  |  |  |  | 1 | 1 | 1 |  |  |  |  | 1 |  | 1 |  |  |  | 1 |  | 1 |  |  | 1 | 1 |  |  | 1 |  |  |  | 1 |  |  | 1 |  |  |  | 1 |  |  |  |  | 1 |  |  |  |  | 1 |  |  |  | 1 |  |  | 1 | 1 |  |  | 1 | 1 | 1 |  |  |  |  | 1 |  |  | 1 |  |  |  | 1 | 1 | 1 | 1 |  |  |  |  | 1 |  | 1 |  |  |  | 1 |  | 1 |  |  | 1 | 1 |  |  | 1 |  |  |
| 145 | 1 | 4 | 5 | 1 |  | 1 |  |  |  |  | 1 |  |  |  |  |  | 1 | 1 |  |  |  |  |  | 1 |  |  |  |  | 1 | 1 | 1 | 1 |  | 1 | 1 | 1 |  |  |  |  | 1 |  |  |  |  |  | 1 |  | 1 |  | 1 |  |  |  | 1 |  |  | 1 |  |  |  | 1 |  |  |  |  |  |  | 1 |  | 1 |  |  |  | 1 |  |  | 1 |  |  |  | 1 |  |  |  |  | 1 |  |  |  |  | 1 |  |  |  | 1 |  |  | 1 | 1 | 1 | 1 | 1 | 1 | 1 |  |  |  |  | 1 | 1 |  | 1 |  |  |  | 1 | 1 | 1 | 1 |  |  |  | 1 |  |  | 1 |  |  |  | 1 |  |  |  |  |  | 1 |  |  | 1 |  |  |
| 146 | 1 | 3 | 2 | 1 |  | 1 |  |  | 1 |  |  |  | 1 |  |  |  |  | 1 |  |  |  |  | 1 |  |  |  |  |  | 1 |  | 1 |  |  | 1 | 1 | 1 |  |  |  |  | 1 |  |  | 1 |  |  |  |  | 1 |  | 1 |  |  |  | 1 |  |  | 1 |  |  |  | 1 |  | 1 |  |  | 1 | 1 |  |  | 1 |  |  |  | 1 |  |  | 1 |  |  |  | 1 |  |  |  |  | 1 |  |  |  |  | 1 |  |  |  | 1 | 1 |  | 1 | 1 |  |  | 1 | 1 | 1 |  |  |  |  | 1 |  |  | 1 |  |  |  | 1 | 1 | 1 | 1 |  |  |  | 1 |  |  | 1 |  |  |  | 1 |  | 1 |  |  | 1 |  |  |  | 1 |  |  |
| 147 | 1 | 4 | 2 | 1 |  | 1 |  |  |  |  |  | 1 |  |  |  |  | 1 | 1 |  |  |  |  | 1 |  |  |  |  |  | 1 |  | 1 | 1 | 1 | 1 |  | 1 |  |  |  |  | 1 | 1 |  |  |  |  | 1 |  | 1 |  | 1 |  |  |  | 1 |  |  | 1 |  |  |  | 1 |  |  |  |  |  | 1 |  |  | 1 |  |  |  | 1 |  |  | 1 |  |  |  | 1 |  |  |  |  | 1 |  |  |  |  | 1 |  |  |  | 1 |  |  | 1 | 1 | 1 |  | 1 | 1 | 1 |  |  |  |  |  | 1 |  | 1 |  |  |  |  | 1 | 1 | 1 |  |  |  | 1 | 1 |  | 1 |  |  |  | 1 |  | 1 |  |  | 1 | 1 |  |  | 1 |  |  |
| 148 | 2 | 4 | 3 | 1 | 1 |  |  |  |  |  |  | 1 |  |  |  |  | 1 | 1 |  |  |  |  | 1 |  |  |  |  |  | 1 | 1 |  |  |  | 1 | 1 |  |  |  |  |  |  | 1 |  |  |  |  | 1 |  | 1 |  | 1 |  |  |  | 1 |  |  |  | 1 |  |  | 1 |  | 1 |  |  |  | 1 |  |  | 1 |  |  |  | 1 |  |  | 1 |  |  |  | 1 |  |  | 1 |  | 1 |  |  |  |  | 1 |  |  |  |  | 1 |  | 1 | 1 |  |  | 1 | 1 | 1 |  |  |  |  | 1 |  |  | 1 |  |  |  | 1 | 1 | 1 | 1 |  |  |  | 1 |  |  | 1 |  |  |  | 1 |  | 1 |  |  | 1 |  |  |  | 1 |  |  |
| 149 | 1 | 3 | 2 | 5 | 1 |  |  |  | 1 |  |  |  |  |  |  | 1 |  | 1 |  |  |  |  | 1 |  |  |  |  |  | 1 | 1 | 1 |  |  | 1 | 1 |  |  |  |  |  | 1 |  |  | 1 |  |  |  |  | 1 |  | 1 |  |  |  |  | 1 |  |  | 1 |  |  | 1 |  | 1 |  |  |  |  |  | 1 | 1 |  |  |  | 1 |  |  | 1 |  |  |  | 1 |  |  | 1 |  | 1 |  |  |  |  | 1 |  |  |  | 1 |  |  | 1 | 1 |  |  | 1 | 1 |  |  |  |  |  | 1 |  |  | 1 |  |  |  | 1 | 1 | 1 | 1 |  |  |  | 1 |  |  | 1 |  |  |  | 1 |  | 1 |  |  | 1 |  |  |  | 1 |  |  |
| 150 | 1 | 4 | 2 | 3 |  | 1 |  |  | 1 |  |  |  |  |  |  | 1 |  |  | 1 |  |  |  |  | 1 |  |  |  |  | 1 |  |  |  |  |  | 1 |  |  |  |  |  | 1 |  |  | 1 |  |  |  |  |  | 1 |  |  |  |  | 1 |  |  |  |  | 1 |  | 1 |  |  |  |  | 1 |  |  |  | 1 |  |  |  | 1 |  |  | 1 |  |  |  | 1 |  |  |  |  | 1 |  |  |  |  | 1 |  |  |  |  | 1 |  | 1 |  |  |  | 1 | 1 | 1 |  |  |  |  | 1 |  |  | 1 |  |  |  |  | 1 | 1 |  |  |  |  | 1 |  |  | 1 |  |  |  | 1 |  |  |  |  | 1 |  |  |  | 1 |  |  |
| 151 | 2 | 3 | 4 | 3 |  | 1 |  |  | 1 |  |  |  |  |  |  | 1 |  | 1 |  |  |  |  |  | 1 |  |  |  |  | 1 |  |  |  |  | 1 | 1 |  |  |  | 1 | 1 |  |  |  | 1 |  |  |  |  | 1 |  | 1 |  |  |  | 1 | 1 |  | 1 |  |  |  | 1 |  | 1 |  |  |  | 1 |  |  | 1 |  |  |  | 1 |  |  | 1 |  |  |  | 1 |  |  | 1 |  | 1 |  |  |  |  | 1 |  |  |  | 1 |  |  | 1 | 1 |  |  | 1 | 1 | 1 |  |  |  |  | 1 |  |  | 1 |  |  |  | 1 | 1 | 1 | 1 |  |  |  | 1 |  |  | 1 |  |  |  | 1 |  | 1 |  |  | 1 | 1 |  |  | 1 |  |  |
| 152 | 2 | 4 | 3 | 1 |  | 1 |  | 1 |  |  |  |  | 1 |  |  |  |  | 1 |  |  |  |  | 1 |  |  |  |  |  | 1 |  |  |  |  | 1 | 1 |  |  |  |  |  | 1 |  |  | 1 |  |  |  |  | 1 |  | 1 |  |  |  |  | 1 |  |  | 1 |  |  | 1 |  |  |  |  |  | 1 |  |  | 1 |  |  |  | 1 |  |  | 1 |  |  |  | 1 |  |  |  |  | 1 |  |  |  |  | 1 |  |  |  | 1 |  |  | 1 | 1 |  |  | 1 | 1 |  |  |  |  |  | 1 |  |  | 1 |  |  |  | 1 | 1 |  | 1 |  |  |  | 1 | 1 |  | 1 |  |  |  | 1 |  | 1 |  |  | 1 | 1 |  |  | 1 |  |  |
| 153 | 1 | 3 | 2 | 1 |  | 1 |  |  | 1 |  |  |  |  |  |  | 1 |  |  | 1 |  |  |  |  | 1 |  |  |  |  | 1 |  | 1 |  |  | 1 | 1 |  |  |  |  |  | 1 |  |  |  |  |  | 1 |  | 1 |  | 1 |  |  |  | 1 |  |  | 1 |  |  |  |  |  | 1 |  |  | 1 |  |  |  | 1 |  |  |  | 1 |  |  | 1 |  |  |  | 1 |  |  | 1 |  | 1 |  |  |  |  | 1 |  |  |  | 1 | 1 |  | 1 | 1 |  |  | 1 | 1 | 1 |  |  |  |  | 1 |  |  |  |  | 1 |  | 1 | 1 | 1 | 1 |  |  |  | 1 | 1 |  | 1 |  |  |  | 1 |  | 1 |  |  | 1 | 1 |  |  | 1 |  |  |
| 154 | 2 | 3 | 4 | 1 |  | 1 |  |  | 1 |  |  |  | 1 |  |  |  |  |  | 1 |  |  |  | 1 |  |  |  |  |  | 1 |  |  |  |  |  | 1 | 1 | 1 |  |  |  |  | 1 |  |  |  |  | 1 | 1 | 1 |  | 1 |  |  |  |  | 1 |  | 1 |  |  |  | 1 |  | 1 |  |  |  | 1 |  |  | 1 |  |  |  | 1 |  |  | 1 |  |  |  | 1 |  |  |  |  |  | 1 |  |  |  | 1 |  |  |  | 1 |  |  |  |  |  |  | 1 | 1 | 1 |  |  |  |  |  | 1 |  |  |  |  | 1 | 1 | 1 | 1 | 1 |  |  |  | 1 |  |  | 1 |  |  |  | 1 |  | 1 |  |  | 1 | 1 |  |  | 1 |  |  |
| 155 | 1 | 4 | 3 | 1 |  | 1 |  |  | 1 |  |  |  | 1 |  |  |  |  | 1 |  |  |  |  | 1 |  |  |  |  | 1 |  | 1 | 1 |  |  | 1 |  | 1 |  |  |  |  | 1 |  |  |  |  | 1 |  | 1 | 1 | 1 | 1 |  |  |  | 1 |  |  | 1 |  |  |  | 1 |  | 1 |  |  | 1 | 1 |  |  | 1 |  |  |  | 1 |  |  | 1 |  |  |  | 1 |  |  | 1 |  | 1 |  |  |  |  | 1 |  |  |  | 1 | 1 |  | 1 | 1 |  |  | 1 | 1 | 1 |  |  |  |  | 1 |  |  |  |  | 1 |  | 1 | 1 | 1 | 1 |  |  |  | 1 | 1 |  | 1 |  |  |  | 1 |  | 1 |  |  | 1 | 1 |  |  | 1 |  |  |
| 156 | 1 | 2 | 2 | 4 |  | 1 |  |  |  | 1 |  |  | 1 |  |  |  |  | 1 |  |  |  |  |  | 1 |  |  |  |  | 1 | 1 | 1 |  |  |  |  |  |  |  |  |  | 1 |  |  |  |  | 1 |  | 1 | 1 |  | 1 |  |  |  | 1 | 1 |  | 1 |  |  |  | 1 |  | 1 |  |  | 1 | 1 |  |  | 1 |  |  |  | 1 |  |  | 1 |  |  |  | 1 |  |  | 1 |  | 1 |  |  |  |  | 1 |  |  |  | 1 |  |  | 1 | 1 |  |  | 1 |  | 1 |  |  |  |  | 1 | 1 |  |  | 1 |  |  | 1 | 1 | 1 | 1 |  |  |  | 1 | 1 |  | 1 |  |  |  | 1 |  | 1 |  |  | 1 | 1 |  |  | 1 |  |  |
| 157 | 1 | 5 | 4 | 2 |  | 1 |  |  | 1 |  |  |  | 1 |  |  | 1 |  |  | 1 |  |  |  | 1 |  |  |  | 1 | 1 |  | 1 | 1 |  |  | 1 | 1 | 1 |  |  |  |  | 1 | 1 |  |  |  | 1 |  | 1 | 1 | 1 | 1 |  |  |  | 1 | 1 |  | 1 |  |  |  | 1 |  | 1 |  |  | 1 | 1 |  |  | 1 |  |  | 1 |  |  |  | 1 |  |  |  |  |  |  | 1 |  | 1 |  |  |  |  | 1 |  |  |  | 1 |  |  |  |  |  |  | 1 | 1 |  |  |  |  |  | 1 |  |  | 1 |  |  |  |  | 1 |  | 1 |  |  |  | 1 |  |  | 1 |  |  |  | 1 |  | 1 |  |  | 1 |  |  |  | 1 |  |  |
| 158 | 1 | 2 | 3 | 1 |  | 1 |  | 1 |  |  |  |  |  |  | 1 |  |  | 1 |  |  |  |  |  | 1 |  |  |  |  | 1 | 1 | 1 |  |  | 1 | 1 |  |  |  |  |  |  | 1 |  |  |  |  | 1 |  |  |  | 1 |  |  |  |  | 1 |  |  | 1 |  |  | 1 |  | 1 |  |  |  |  |  | 1 | 1 |  |  |  | 1 |  |  | 1 |  |  |  | 1 |  |  |  |  | 1 |  |  |  |  | 1 |  |  |  |  |  | 1 | 1 | 1 |  |  | 1 | 1 |  |  |  |  |  |  | 1 |  | 1 |  |  |  |  | 1 | 1 | 1 |  |  |  | 1 |  |  | 1 |  |  |  | 1 |  | 1 |  |  | 1 |  |  |  | 1 |  |  |
| 159 | 2 | 4 | 2 | 4 |  | 1 |  |  | 1 |  |  |  |  |  |  | 1 |  | 1 |  |  |  |  | 1 |  |  |  |  |  | 1 |  | 1 |  |  | 1 | 1 | 1 |  |  |  |  |  |  | 1 | 1 |  |  |  | 1 | 1 |  | 1 |  |  |  | 1 | 1 |  | 1 |  |  |  | 1 |  | 1 |  |  | 1 | 1 |  |  | 1 |  |  |  | 1 |  |  | 1 |  |  |  | 1 |  |  | 1 |  | 1 |  |  |  |  | 1 |  |  |  | 1 |  |  | 1 | 1 |  |  | 1 | 1 | 1 |  |  |  |  | 1 |  |  |  |  | 1 |  |  | 1 | 1 | 1 |  |  |  | 1 | 1 |  | 1 |  |  |  | 1 |  | 1 |  |  | 1 | 1 |  |  | 1 |  |  |
| 160 | 1 | 4 | 2 | 1 | 1 |  |  | 1 |  |  |  |  |  |  |  |  | 1 | 1 |  |  |  |  |  | 1 |  | 1 |  |  |  | 1 | 1 |  |  | 1 | 1 | 1 |  |  |  |  | 1 |  |  |  |  |  | 1 |  | 1 |  | 1 |  |  |  | 1 |  |  | 1 |  |  |  | 1 |  |  |  |  |  | 1 |  |  |  |  | 1 |  | 1 |  |  | 1 |  |  |  | 1 |  |  | 1 |  | 1 |  |  |  |  | 1 |  |  |  | 1 |  |  | 1 | 1 |  |  | 1 | 1 | 1 |  |  |  | 1 | 1 |  |  |  |  | 1 |  | 1 | 1 | 1 | 1 |  |  |  | 1 |  |  | 1 |  |  |  | 1 |  |  |  |  | 1 | 1 |  |  | 1 |  |  |
| 161 | 2 | 4 | 4 | 1 |  | 1 |  |  |  |  |  | 1 |  |  |  |  | 1 |  | 1 |  |  |  | 1 |  |  |  |  |  | 1 |  |  |  |  |  |  |  | 1 |  |  |  |  |  | 1 |  |  |  | 1 |  | 1 |  | 1 |  |  |  | 1 |  |  |  | 1 |  |  | 1 |  |  |  |  | 1 |  |  |  | 1 |  |  |  | 1 |  |  | 1 |  |  |  |  |  |  | 1 |  | 1 |  |  |  |  | 1 |  |  |  | 1 |  |  | 1 | 1 |  |  | 1 | 1 |  |  |  |  |  | 1 |  |  |  |  | 1 |  | 1 | 1 |  | 1 |  |  |  | 1 |  |  | 1 |  |  |  | 1 |  |  |  |  | 1 |  |  |  | 1 |  |  |
| 162 | 1 | 4 | 1 | 1 |  | 1 |  | 1 |  |  |  |  |  |  |  |  | 1 | 1 |  |  |  |  | 1 |  |  |  |  |  | 1 |  | 1 |  |  | 1 | 1 |  |  |  |  |  | 1 |  |  | 1 |  |  |  |  | 1 |  | 1 |  |  |  |  | 1 |  | 1 |  |  |  | 1 |  |  |  |  |  | 1 |  |  | 1 |  |  |  | 1 |  |  | 1 |  |  |  | 1 |  |  |  |  | 1 |  |  |  |  | 1 |  |  |  | 1 |  |  | 1 | 1 |  |  | 1 | 1 |  |  |  |  |  | 1 |  |  | 1 |  |  |  | 1 | 1 |  | 1 |  |  |  | 1 | 1 |  | 1 |  |  |  | 1 |  | 1 |  |  | 1 | 1 |  |  | 1 |  |  |
| 163 | 1 | 2 | 3 | 1 |  | 1 |  | 1 |  |  |  |  |  |  |  | 1 |  | 1 |  |  |  |  |  | 1 |  | 1 |  |  |  |  | 1 |  |  | 1 | 1 |  |  |  |  |  | 1 |  |  |  |  |  | 1 |  | 1 |  | 1 |  |  |  | 1 | 1 |  | 1 |  |  |  | 1 |  | 1 |  |  | 1 | 1 |  |  | 1 |  |  |  | 1 |  |  | 1 |  |  |  | 1 |  |  | 1 |  | 1 |  |  |  |  | 1 |  |  |  | 1 |  |  | 1 | 1 |  |  | 1 | 1 |  |  |  |  |  |  | 1 |  |  |  | 1 |  |  | 1 |  | 1 |  |  |  | 1 | 1 |  |  | 1 |  |  | 1 |  | 1 |  |  |  | 1 |  |  | 1 |  |  |
| 164 | 1 | 1 | 1 | 1 |  |  | 1 | 1 |  |  |  |  |  |  |  |  | 1 |  | 1 |  |  |  | 1 |  |  |  |  |  | 1 |  | 1 |  |  | 1 | 1 |  |  |  |  |  | 1 |  |  |  | 1 |  |  |  | 1 |  | 1 |  |  |  | 1 | 1 |  |  |  | 1 |  | 1 |  | 1 |  |  | 1 |  |  |  | 1 |  |  | 1 |  |  |  | 1 |  |  |  | 1 |  |  | 1 |  |  | 1 |  |  |  | 1 |  |  |  |  |  | 1 | 1 |  |  |  | 1 | 1 |  |  |  |  |  | 1 |  |  | 1 |  |  |  |  | 1 |  | 1 |  |  |  | 1 |  |  | 1 |  |  |  | 1 |  | 1 |  |  | 1 |  |  |  | 1 |  |  |
| 165 | 1 | 4 | 2 | 3 |  | 1 |  |  | 1 |  |  |  |  |  |  | 1 |  | 1 |  |  |  |  |  | 1 |  |  |  |  | 1 | 1 |  |  |  | 1 | 1 | 1 |  |  |  |  |  | 1 |  | 1 |  |  |  |  | 1 |  | 1 |  |  |  | 1 | 1 |  | 1 |  |  |  | 1 |  | 1 |  |  | 1 | 1 |  |  |  |  | 1 |  | 1 |  |  | 1 |  |  |  |  |  |  | 1 |  | 1 |  |  |  |  | 1 |  |  |  | 1 |  |  | 1 | 1 |  |  | 1 | 1 |  |  |  |  |  |  | 1 |  | 1 |  |  |  | 1 | 1 |  | 1 |  |  |  | 1 |  |  | 1 |  |  |  | 1 |  | 1 |  |  | 1 |  |  |  | 1 |  |  |
| 166 | 2 | 3 | 2 | 2 |  |  | 1 |  |  |  | 1 |  |  |  |  | 1 |  | 1 |  |  |  |  |  | 1 |  |  |  |  | 1 | 1 | 1 |  |  |  | 1 |  |  |  |  |  |  |  | 1 |  |  |  | 1 |  |  |  |  | 1 |  | 1 |  |  |  |  |  | 1 |  | 1 |  |  |  |  |  |  |  | 1 | 1 |  |  |  | 1 |  |  | 1 |  |  |  | 1 |  |  | 1 |  | 1 |  |  |  |  | 1 |  |  |  | 1 |  |  | 1 | 1 |  |  | 1 | 1 |  |  |  |  |  | 1 |  |  |  |  |  | 1 |  | 1 |  | 1 |  |  |  |  | 1 |  | 1 |  |  |  | 1 |  |  |  |  | 1 | 1 |  |  | 1 |  |  |
| 167 | 2 | 2 | 2 | 1 |  | 1 |  |  | 1 |  |  |  | 1 |  |  |  |  | 1 |  |  |  |  |  | 1 |  |  | 1 |  |  | 1 | 1 |  |  | 1 | 1 |  |  |  |  |  | 1 |  |  |  |  | 1 |  |  | 1 |  | 1 |  |  |  |  | 1 |  | 1 |  |  |  | 1 |  |  |  |  |  | 1 |  |  | 1 |  |  |  | 1 |  |  | 1 |  |  |  | 1 |  |  |  |  | 1 |  |  |  |  | 1 |  |  |  | 1 |  |  | 1 | 1 |  |  | 1 | 1 |  |  |  |  |  | 1 |  |  |  |  | 1 |  |  | 1 |  | 1 |  |  |  | 1 |  |  | 1 |  |  |  | 1 |  |  |  |  |  | 1 |  |  | 1 |  |  |
| 168 | 1 | 2 | 2 | 2 |  | 1 |  |  | 1 |  |  |  | 1 |  |  |  |  | 1 |  |  |  |  | 1 |  |  |  |  |  | 1 |  |  |  |  |  | 1 |  |  |  |  |  | 1 |  |  |  |  |  | 1 |  |  |  | 1 |  |  |  | 1 |  |  | 1 |  |  |  | 1 |  |  |  |  | 1 |  |  |  | 1 |  |  |  | 1 |  |  | 1 |  |  |  | 1 |  |  |  |  | 1 |  |  |  |  | 1 |  |  |  | 1 | 1 |  | 1 | 1 |  |  | 1 | 1 | 1 |  |  |  |  | 1 |  |  |  |  | 1 |  | 1 | 1 | 1 | 1 |  |  |  | 1 |  |  | 1 |  |  |  | 1 |  | 1 |  |  | 1 | 1 |  |  | 1 |  |  |
| 169 | 2 | 3 | 2 | 1 | 1 |  |  |  | 1 |  |  |  | 1 |  |  |  |  | 1 |  |  |  |  | 1 |  |  |  |  |  | 1 | 1 | 1 |  |  | 1 | 1 |  |  |  |  |  | 1 |  |  |  | 1 |  |  |  | 1 |  | 1 |  |  |  |  | 1 |  | 1 |  |  |  | 1 |  |  |  |  |  |  | 1 |  |  |  | 1 |  | 1 |  |  | 1 |  |  |  | 1 |  |  |  |  | 1 |  |  |  |  | 1 |  |  |  | 1 |  |  | 1 | 1 |  |  | 1 |  | 1 |  |  |  |  | 1 |  |  |  |  | 1 |  |  | 1 |  | 1 |  |  |  | 1 |  |  | 1 |  |  |  | 1 |  | 1 |  |  | 1 | 1 |  |  | 1 |  |  |
| 170 | 1 | 3 | 2 | 1 |  | 1 |  | 1 |  |  |  |  |  |  |  | 1 |  | 1 |  |  |  |  | 1 |  |  |  |  |  | 1 | 1 | 1 |  |  |  |  |  |  |  |  |  | 1 |  |  | 1 |  |  |  |  | 1 |  | 1 |  |  |  | 1 |  |  | 1 |  |  |  | 1 |  | 1 |  |  |  | 1 |  |  | 1 |  |  |  | 1 |  |  | 1 |  |  |  | 1 |  |  | 1 |  | 1 |  |  |  |  | 1 |  |  |  | 1 |  |  | 1 | 1 |  |  | 1 | 1 | 1 |  |  |  |  | 1 |  |  |  |  | 1 |  |  | 1 | 1 | 1 |  |  |  |  | 1 |  | 1 |  |  |  | 1 |  | 1 |  |  | 1 | 1 |  |  | 1 |  |  |
| 171 | 1 | 4 | 3 | 1 |  | 1 |  |  | 1 |  |  |  |  |  |  |  | 1 | 1 |  |  |  |  | 1 |  |  |  | 1 |  |  | 1 |  |  |  |  |  |  |  |  |  |  |  | 1 |  |  |  | 1 |  |  | 1 |  | 1 |  |  |  | 1 |  |  | 1 |  |  |  | 1 |  | 1 |  |  | 1 | 1 |  |  | 1 |  |  |  | 1 |  |  | 1 |  |  |  | 1 |  |  |  |  | 1 |  |  |  |  | 1 |  |  |  | 1 |  |  | 1 | 1 |  |  | 1 | 1 | 1 |  |  |  |  |  | 1 |  |  |  | 1 |  | 1 | 1 | 1 | 1 |  |  |  | 1 |  |  | 1 |  |  |  | 1 |  | 1 |  |  | 1 | 1 |  |  | 1 |  |  |
| 172 | 2 | 2 | 3 | 3 |  |  | 1 |  |  |  | 1 |  |  |  |  |  | 1 |  | 1 |  |  |  |  | 1 |  |  |  |  | 1 |  |  |  |  |  |  |  | 1 |  | 1 |  |  |  |  |  | 1 |  |  |  |  |  | 1 |  |  | 1 |  |  |  |  |  | 1 |  |  |  |  | 1 |  |  | 1 |  |  | 1 |  |  |  | 1 |  |  | 1 |  |  |  | 1 |  |  |  |  | 1 |  |  |  |  | 1 |  |  |  |  |  | 1 | 1 | 1 |  |  | 1 | 1 |  |  |  |  |  |  | 1 |  | 1 |  |  |  |  | 1 | 1 | 1 |  |  |  | 1 |  |  | 1 |  |  |  | 1 |  | 1 |  |  | 1 |  |  |  | 1 |  |  |
| 173 | 2 | 4 | 2 | 1 | 1 |  |  |  | 1 |  |  |  |  |  |  | 1 |  | 1 | 1 |  |  |  |  | 1 |  |  |  |  | 1 |  |  |  |  | 1 | 1 |  |  |  |  |  | 1 |  |  |  |  |  | 1 |  | 1 |  | 1 |  |  |  | 1 |  |  | 1 |  |  |  | 1 |  | 1 |  |  |  | 1 |  |  | 1 |  |  |  | 1 |  |  | 1 |  |  |  |  |  |  | 1 |  |  | 1 |  |  |  | 1 |  |  |  | 1 |  |  | 1 | 1 |  |  | 1 | 1 |  |  |  |  |  | 1 |  |  |  |  | 1 |  |  | 1 | 1 | 1 |  |  |  | 1 | 1 |  | 1 |  |  |  | 1 |  | 1 |  |  | 1 | 1 |  |  | 1 |  |  |
| 174 | 1 | 5 | 3 | 3 |  | 1 |  |  | 1 |  |  |  |  |  |  | 1 |  | 1 |  |  |  |  | 1 |  |  |  |  |  | 1 | 1 | 1 |  |  | 1 | 1 |  |  |  |  |  |  |  | 1 | 1 |  |  |  |  |  |  | 1 |  |  |  |  |  | 1 |  | 1 |  |  | 1 |  | 1 |  |  |  |  | 1 |  | 1 |  |  |  | 1 |  |  | 1 |  |  |  | 1 |  |  | 1 |  | 1 |  |  |  |  | 1 |  |  |  | 1 |  |  | 1 | 1 |  |  | 1 | 1 | 1 |  |  |  |  |  | 1 |  |  |  | 1 |  | 1 | 1 | 1 | 1 |  |  |  | 1 | 1 |  | 1 |  |  |  | 1 |  | 1 |  |  |  | 1 |  |  | 1 |  |  |
| 175 | 2 | 3 | 4 | 5 | 1 |  |  |  | 1 |  |  |  |  |  |  | 1 |  | 1 |  |  |  |  | 1 |  |  |  |  | 1 |  | 1 | 1 |  |  | 1 | 1 |  |  |  |  |  | 1 |  |  |  | 1 |  |  |  | 1 |  | 1 |  |  |  | 1 |  |  |  | 1 |  |  | 1 |  |  |  | 1 |  |  |  |  |  | 1 |  |  | 1 |  |  | 1 |  |  |  | 1 |  |  |  |  | 1 |  |  |  |  | 1 |  |  |  | 1 | 1 |  | 1 | 1 |  |  | 1 | 1 | 1 |  |  |  |  | 1 |  |  |  |  | 1 |  | 1 | 1 | 1 | 1 |  |  |  | 1 | 1 |  | 1 |  |  |  | 1 |  |  |  |  |  | 1 |  |  | 1 |  |  |
| 176 | 2 | 3 | 4 | 1 |  | 1 |  |  |  |  | 1 |  |  |  |  |  | 1 | 1 |  |  |  |  | 1 |  |  |  |  |  | 1 |  | 1 |  |  | 1 | 1 | 1 |  |  |  |  | 1 |  |  |  | 1 |  |  | 1 | 1 |  | 1 |  |  |  | 1 |  |  | 1 |  |  |  | 1 |  | 1 |  |  | 1 | 1 |  |  | 1 |  |  |  | 1 |  |  | 1 |  |  |  | 1 |  |  |  |  | 1 |  |  |  |  | 1 |  |  |  | 1 |  |  | 1 | 1 |  |  | 1 | 1 | 1 |  |  |  |  | 1 | 1 |  | 1 |  | 1 |  | 1 | 1 | 1 | 1 |  |  |  | 1 | 1 |  | 1 |  |  |  | 1 |  | 1 |  |  | 1 | 1 |  |  | 1 |  |  |
| 177 | 2 | 4 | 4 | 2 |  | 1 |  | 1 |  | 1 |  |  |  |  |  | 1 |  | 1 |  |  |  |  | 1 |  |  |  | 1 |  |  |  | 1 |  |  | 1 | 1 |  |  |  |  |  |  | 1 |  | 1 |  |  |  |  | 1 |  |  |  |  |  | 1 |  |  |  | 1 |  |  | 1 |  |  |  |  | 1 | 1 |  |  | 1 |  |  |  | 1 |  |  | 1 |  |  |  | 1 |  |  |  |  | 1 |  |  |  |  | 1 |  |  |  | 1 |  |  | 1 | 1 |  |  | 1 | 1 | 1 |  |  |  |  |  | 1 |  |  |  | 1 |  | 1 | 1 | 1 | 1 |  |  |  | 1 |  |  | 1 |  |  |  | 1 |  | 1 |  |  | 1 | 1 |  |  | 1 |  |  |
| 178 | 2 | 4 | 3 | 1 |  |  | 1 |  | 1 |  |  |  |  |  |  | 1 |  |  | 1 |  |  |  |  | 1 |  | 1 |  |  |  |  | 1 |  |  | 1 | 1 |  |  |  |  |  | 1 |  |  |  | 1 |  |  |  | 1 |  | 1 |  |  |  |  | 1 |  |  | 1 |  |  | 1 |  | 1 |  |  |  |  |  | 1 |  |  | 1 |  | 1 |  |  | 1 |  |  |  | 1 |  |  | 1 |  | 1 |  |  |  |  | 1 |  |  |  | 1 | 1 |  | 1 | 1 |  |  | 1 | 1 | 1 |  |  |  |  |  | 1 |  | 1 |  |  |  |  | 1 |  | 1 |  |  |  | 1 |  |  |  | 1 |  |  | 1 |  | 1 |  |  | 1 | 1 |  |  | 1 |  |  |
| 179 | 2 | 4 | 1 | 1 |  |  | 1 |  | 1 |  |  |  |  |  |  | 1 |  | 1 |  |  |  |  | 1 |  |  |  |  |  | 1 |  |  |  |  | 1 | 1 |  |  |  |  |  | 1 |  |  |  | 1 |  |  |  | 1 |  | 1 |  |  |  | 1 | 1 |  |  |  | 1 |  | 1 |  | 1 |  |  | 1 |  |  |  | 1 |  |  | 1 |  |  |  | 1 |  |  |  | 1 |  |  |  |  | 1 |  |  |  |  | 1 |  |  |  | 1 | 1 |  | 1 | 1 |  |  | 1 | 1 | 1 |  |  |  |  | 1 | 1 |  | 1 |  |  |  | 1 | 1 | 1 | 1 |  |  |  | 1 |  |  | 1 |  |  |  | 1 |  | 1 |  |  | 1 | 1 |  |  | 1 |  |  |
| 180 | 2 | 2 | 2 | 3 |  |  | 1 |  |  | 1 |  |  |  |  |  |  | 1 |  | 1 |  |  |  |  |  | 1 |  |  |  | 1 | 1 | 1 | 1 |  | 1 | 1 | 1 |  |  |  |  | 1 |  |  |  |  |  | 1 | 1 | 1 |  | 1 |  |  |  | 1 |  |  | 1 |  |  |  | 1 |  | 1 |  |  | 1 | 1 |  |  | 1 |  |  |  | 1 |  |  | 1 |  |  |  | 1 |  |  |  |  | 1 |  |  |  |  | 1 |  |  |  |  | 1 |  | 1 | 1 | 1 |  | 1 | 1 | 1 |  |  |  |  | 1 |  |  | 1 |  |  |  | 1 | 1 | 1 | 1 |  |  |  | 1 |  |  | 1 |  |  |  | 1 |  | 1 |  |  | 1 | 1 |  |  | 1 |  |  |
| 181 | 2 | 4 | 2 | 1 |  | 1 |  |  | 1 |  |  |  |  |  |  |  | 1 |  | 1 |  |  |  |  | 1 |  |  |  |  | 1 | 1 | 1 |  |  | 1 | 1 |  |  |  |  |  |  | 1 |  | 1 |  |  |  |  | 1 |  | 1 |  |  |  | 1 |  |  | 1 |  |  |  | 1 |  | 1 |  |  |  |  |  | 1 | 1 |  |  |  | 1 |  |  | 1 |  |  |  |  |  |  |  | 1 |  | 1 |  |  |  |  | 1 |  |  |  |  | 1 | 1 | 1 |  |  | 1 | 1 | 1 |  |  |  |  |  | 1 |  | 1 |  |  |  |  | 1 |  | 1 |  |  |  | 1 |  |  | 1 |  |  |  | 1 |  | 1 |  | 1 |  |  |  |  | 1 |  |  |
| 182 | 1 | 1 | 1 | 1 |  | 1 |  |  | 1 |  |  |  | 1 |  |  |  |  | 1 |  |  |  |  | 1 |  |  |  |  |  | 1 | 1 | 1 |  |  | 1 | 1 |  |  |  |  |  | 1 |  |  |  | 1 |  |  |  | 1 | 1 | 1 |  |  |  |  | 1 |  | 1 |  |  |  | 1 |  |  |  |  |  | 1 |  |  | 1 |  |  |  | 1 |  |  | 1 |  |  |  | 1 |  |  |  |  | 1 |  |  |  |  | 1 |  |  |  | 1 |  |  | 1 | 1 |  |  | 1 | 1 |  |  |  |  |  |  | 1 |  | 1 |  |  |  |  | 1 |  | 1 |  |  |  | 1 |  |  | 1 |  |  |  | 1 |  | 1 |  |  |  | 1 |  |  | 1 |  |  |
| 183 | 1 | 4 | 2 | 4 |  | 1 |  |  | 1 |  |  |  |  |  |  | 1 |  | 1 |  |  |  |  | 1 |  |  | 1 |  |  |  | 1 |  |  |  |  | 1 | 1 |  |  |  |  | 1 |  |  |  |  | 1 |  |  | 1 |  | 1 |  |  |  | 1 |  |  | 1 |  |  |  | 1 |  | 1 |  |  |  | 1 |  |  | 1 |  |  | 1 |  |  |  | 1 |  |  |  | 1 |  |  |  |  | 1 |  |  |  |  | 1 |  |  |  | 1 |  |  | 1 | 1 |  |  | 1 | 1 | 1 |  |  |  |  | 1 |  |  | 1 |  |  |  | 1 | 1 |  | 1 |  |  |  |  | 1 |  | 1 |  |  |  | 1 |  | 1 |  |  | 1 |  |  |  | 1 |  |  |
| 184 | 1 | 4 | 1 | 1 |  |  | 1 |  |  |  |  | 1 |  |  |  |  | 1 | 1 |  |  |  |  |  | 1 |  |  |  |  | 1 | 1 | 1 |  |  | 1 | 1 |  |  |  |  |  | 1 |  |  |  |  |  | 1 | 1 | 1 | 1 | 1 |  |  |  | 1 |  |  | 1 |  |  |  | 1 |  | 1 |  |  | 1 | 1 |  |  | 1 |  |  |  | 1 |  |  | 1 |  |  |  | 1 |  |  |  |  | 1 |  |  |  |  | 1 |  |  |  | 1 |  |  | 1 | 1 | 1 |  | 1 | 1 | 1 |  |  |  |  |  | 1 |  |  |  | 1 |  | 1 | 1 | 1 | 1 |  |  |  | 1 |  |  | 1 |  |  |  | 1 |  | 1 |  |  | 1 | 1 |  |  | 1 |  |  |
| 185 | 1 | 3 | 2 | 6 |  | 1 |  |  | 1 |  |  |  | 1 |  |  |  |  | 1 |  |  |  |  | 1 |  |  |  | 1 | 1 |  | 1 | 1 |  |  | 1 | 1 |  |  |  |  |  |  | 1 |  | 1 |  |  |  | 1 | 1 | 1 | 1 |  |  |  | 1 |  |  | 1 |  |  |  | 1 |  |  |  |  | 1 | 1 |  |  | 1 |  |  |  | 1 |  |  | 1 |  |  |  | 1 |  |  |  |  | 1 |  |  |  |  | 1 |  |  |  | 1 | 1 |  | 1 | 1 |  |  | 1 | 1 |  |  |  |  |  |  | 1 |  | 1 |  |  |  | 1 | 1 | 1 | 1 |  |  |  | 1 |  |  | 1 |  |  |  | 1 |  | 1 |  |  | 1 | 1 |  |  | 1 |  |  |
| 186 | 2 | 2 | 4 | 1 |  | 1 |  |  | 1 |  |  |  | 1 |  |  |  |  | 1 |  |  |  |  | 1 |  |  |  |  | 1 |  |  |  | 1 |  | 1 |  | 1 |  |  |  |  | 1 |  |  |  | 1 |  |  | 1 | 1 |  | 1 |  |  |  | 1 | 1 |  | 1 |  |  |  | 1 |  | 1 |  |  | 1 |  |  |  | 1 |  |  |  | 1 |  |  | 1 |  |  |  | 1 |  |  | 1 |  | 1 |  |  |  |  | 1 |  |  |  | 1 | 1 |  | 1 | 1 |  |  | 1 | 1 | 1 |  |  |  |  | 1 |  |  |  |  | 1 |  | 1 | 1 | 1 | 1 |  |  |  | 1 | 1 |  | 1 |  |  |  | 1 |  | 1 |  |  | 1 | 1 |  |  | 1 |  |  |
| 187 | 2 | 3 | 4 | 3 | 1 |  |  |  | 1 |  |  |  | 1 |  |  |  |  | 1 |  |  |  |  | 1 |  |  |  |  |  | 1 |  | 1 |  |  | 1 | 1 |  |  |  |  |  | 1 |  |  |  | 1 |  |  |  | 1 |  | 1 |  |  |  |  | 1 |  |  | 1 |  |  | 1 |  | 1 |  |  |  | 1 |  |  | 1 |  |  |  | 1 |  |  | 1 |  |  |  | 1 |  |  | 1 |  | 1 |  |  |  |  | 1 |  |  |  | 1 | 1 |  | 1 | 1 |  |  | 1 | 1 | 1 |  |  |  |  | 1 |  |  | 1 |  |  |  | 1 | 1 | 1 | 1 |  |  |  | 1 | 1 |  | 1 |  |  |  | 1 |  | 1 |  |  | 1 | 1 |  |  | 1 |  |  |
| 188 | 1 | 2 | 4 | 1 |  | 1 |  |  | 1 |  |  |  |  |  |  | 1 |  | 1 |  |  |  |  | 1 |  |  |  | 1 |  |  | 1 | 1 |  |  | 1 | 1 |  |  |  |  |  |  | 1 |  |  |  | 1 |  |  | 1 |  | 1 |  |  |  | 1 | 1 |  | 1 |  |  |  | 1 |  | 1 |  |  | 1 | 1 |  |  | 1 |  |  |  | 1 |  |  | 1 |  |  |  |  |  |  | 1 |  | 1 |  |  |  |  | 1 |  |  |  | 1 |  |  | 1 | 1 |  |  | 1 | 1 | 1 |  |  |  |  |  | 1 |  |  |  | 1 |  |  | 1 | 1 | 1 |  |  |  | 1 | 1 |  | 1 |  |  |  | 1 |  | 1 |  |  | 1 | 1 |  |  | 1 |  |  |
| 189 | 2 | 2 | 2 | 1 |  | 1 |  |  | 1 |  |  |  |  |  |  |  | 1 | 1 |  |  |  |  | 1 |  |  |  | 1 |  |  |  | 1 |  |  | 1 | 1 |  |  |  |  |  |  | 1 |  |  |  |  | 1 | 1 | 1 |  | 1 |  |  |  | 1 | 1 |  |  |  | 1 |  | 1 |  | 1 |  |  | 1 | 1 |  |  | 1 |  |  |  | 1 |  |  | 1 |  |  |  | 1 |  |  |  |  | 1 |  |  |  |  | 1 |  |  |  | 1 |  |  | 1 | 1 |  |  | 1 | 1 |  |  |  |  |  |  | 1 |  | 1 |  |  |  | 1 | 1 | 1 | 1 |  |  |  | 1 |  |  | 1 |  |  |  |  | 1 | 1 |  |  | 1 | 1 |  |  | 1 |  |  |
| 190 | 1 | 3 | 2 | 6 |  | 1 |  |  | 1 |  |  |  |  |  |  | 1 |  | 1 |  |  |  |  |  | 1 |  |  |  |  | 1 | 1 | 1 |  |  | 1 | 1 |  |  |  |  |  |  | 1 |  |  |  |  | 1 | 1 | 1 | 1 | 1 |  |  |  | 1 |  |  | 1 |  |  |  | 1 |  | 1 |  |  | 1 |  |  |  | 1 |  |  |  | 1 |  |  | 1 |  |  |  |  |  |  | 1 |  | 1 |  |  |  |  | 1 |  |  | 1 |  |  |  | 1 |  |  |  | 1 | 1 |  |  |  |  |  |  | 1 |  |  |  | 1 |  | 1 | 1 | 1 | 1 |  |  |  | 1 |  |  | 1 |  |  |  | 1 |  | 1 |  |  | 1 | 1 |  |  | 1 |  |  |
| 191 | 2 | 4 | 3 | 1 |  | 1 |  |  | 1 |  |  |  |  |  |  | 1 |  | 1 |  |  |  |  | 1 |  |  |  | 1 |  |  |  | 1 |  |  | 1 | 1 |  |  |  |  |  | 1 |  |  |  |  | 1 |  |  | 1 |  | 1 |  |  |  | 1 |  |  | 1 |  |  |  | 1 |  |  |  |  | 1 |  |  |  | 1 |  |  |  | 1 |  |  | 1 |  |  |  | 1 |  |  |  |  | 1 |  |  |  |  | 1 |  |  |  | 1 |  |  |  | 1 |  |  | 1 | 1 |  |  |  |  |  | 1 |  |  |  |  | 1 |  | 1 | 1 | 1 | 1 |  |  |  | 1 |  |  | 1 |  |  |  | 1 |  |  |  |  | 1 |  |  |  | 1 |  |  |
| 192 | 1 | 5 | 2 | 3 |  | 1 |  |  | 1 |  |  |  |  |  |  | 1 |  | 1 |  |  |  |  | 1 |  |  |  |  |  | 1 |  | 1 |  |  | 1 | 1 |  |  |  |  |  |  | 1 |  |  |  | 1 |  | 1 | 1 |  | 1 |  |  |  |  | 1 |  | 1 |  |  |  | 1 |  | 1 |  |  | 1 | 1 |  |  | 1 |  |  |  | 1 |  |  | 1 |  |  |  | 1 |  |  |  |  | 1 |  |  |  |  | 1 |  |  |  | 1 |  |  | 1 | 1 |  |  | 1 | 1 | 1 |  |  |  |  | 1 |  |  | 1 |  |  |  | 1 | 1 | 1 | 1 |  |  |  | 1 |  |  | 1 |  |  |  | 1 |  | 1 |  |  | 1 |  |  |  | 1 |  |  |
| 193 | 1 | 2 | 3 | 1 |  |  | 1 |  |  | 1 |  |  |  |  |  |  | 1 |  |  |  | 1 |  |  | 1 |  |  |  |  | 1 | 1 |  |  |  | 1 | 1 |  |  |  |  |  |  |  | 1 |  |  |  | 1 |  | 1 |  | 1 |  |  |  | 1 |  |  | 1 |  |  |  | 1 |  |  |  |  |  | 1 |  |  | 1 |  |  |  | 1 |  | 1 |  |  |  |  |  |  |  |  | 1 |  | 1 |  |  |  | 1 |  |  |  |  |  | 1 |  |  |  |  |  |  |  | 1 |  |  |  | 1 |  |  | 1 |  |  |  |  |  |  |  | 1 |  |  | 1 |  |  | 1 |  |  |  | 1 |  |  |  |  |  |  |  | 1 | 1 |  |  |
| 194 | 1 | 4 | 4 | 1 |  |  | 1 | 1 |  |  |  |  |  |  |  | 1 |  | 1 |  |  |  |  |  | 1 |  |  |  |  | 1 |  |  |  |  | 1 | 1 |  |  |  |  |  |  |  | 1 |  |  |  | 1 |  | 1 |  | 1 |  |  |  | 1 |  |  |  |  | 1 |  | 1 |  |  |  |  | 1 | 1 |  |  | 1 |  |  |  | 1 |  |  | 1 |  |  |  | 1 |  |  | 1 |  | 1 |  |  |  |  | 1 |  |  |  | 1 |  |  |  | 1 |  |  | 1 | 1 |  |  |  |  |  | 1 |  |  |  |  | 1 |  | 1 | 1 | 1 | 1 |  |  |  | 1 |  |  | 1 |  |  |  | 1 |  | 1 |  |  | 1 |  |  |  | 1 |  |  |
| 195 | 1 | 3 | 2 | 1 | 1 |  |  | 1 |  |  |  |  |  |  |  | 1 |  |  | 1 |  |  |  | 1 |  |  |  |  |  | 1 | 1 |  |  |  | 1 |  | 1 |  |  |  | 1 |  |  |  |  |  |  | 1 | 1 | 1 |  | 1 |  |  |  |  | 1 |  | 1 |  |  |  | 1 |  | 1 |  |  | 1 |  |  |  | 1 |  |  |  | 1 |  |  | 1 |  |  |  | 1 |  |  |  |  | 1 |  |  |  |  | 1 |  |  |  | 1 |  |  | 1 | 1 |  |  | 1 | 1 | 1 |  |  | 1 |  |  |  |  |  |  | 1 |  | 1 | 1 |  | 1 |  |  |  |  | 1 |  | 1 |  |  |  | 1 |  | 1 |  |  | 1 |  |  |  | 1 |  |  |
| 196 | 1 | 2 | 1 | 1 | 1 |  |  |  |  | 1 |  |  |  |  |  | 1 |  | 1 |  |  |  |  |  | 1 |  |  |  |  | 1 |  | 1 |  |  | 1 | 1 | 1 |  |  |  |  | 1 | 1 |  | 1 |  |  |  |  | 1 |  | 1 |  |  |  | 1 |  |  |  |  | 1 |  | 1 |  | 1 |  |  |  |  |  | 1 | 1 |  |  | 1 |  |  |  | 1 |  |  |  | 1 |  |  |  |  | 1 |  |  |  |  | 1 |  |  | 1 |  |  |  |  | 1 |  |  | 1 | 1 |  | 1 |  | 1 |  | 1 | 1 |  | 1 |  |  |  |  | 1 | 1 | 1 |  |  |  | 1 |  |  | 1 |  |  |  | 1 |  | 1 |  |  |  | 1 |  |  | 1 |  |  |
| 197 | 1 | 4 | 2 | 1 | 1 |  |  | 1 |  |  |  |  | 1 |  |  |  |  |  | 1 |  |  |  |  | 1 |  | 1 |  |  |  |  |  |  |  | 1 | 1 |  |  |  |  |  |  | 1 |  |  |  |  | 1 |  | 1 |  | 1 |  |  |  | 1 |  |  | 1 |  |  |  | 1 |  | 1 |  |  | 1 | 1 |  |  | 1 |  |  | 1 |  |  | 1 |  |  |  |  | 1 |  |  | 1 |  | 1 |  |  |  |  | 1 |  |  | 1 |  |  |  | 1 | 1 |  |  |  | 1 |  |  |  |  |  |  | 1 |  | 1 |  |  |  |  | 1 |  | 1 |  |  |  | 1 |  |  | 1 |  |  |  | 1 |  | 1 |  |  | 1 |  |  |  | 1 |  |  |
| 198 | 1 | 3 | 5 | 3 |  | 1 |  | 1 |  |  |  |  |  | 1 |  |  |  |  | 1 |  |  |  |  | 1 |  |  |  | 1 |  | 1 | 1 |  |  | 1 |  |  |  |  |  |  | 1 |  |  | 1 |  |  |  |  | 1 |  | 1 |  |  |  | 1 |  |  | 1 |  |  |  | 1 |  | 1 |  |  |  | 1 |  |  | 1 |  |  |  | 1 |  |  | 1 |  |  |  |  |  |  | 1 |  |  | 1 |  |  |  | 1 |  |  |  | 1 |  |  | 1 | 1 |  |  | 1 | 1 |  |  |  |  |  | 1 |  |  | 1 |  |  |  |  | 1 |  | 1 |  |  |  | 1 |  |  |  |  | 1 |  | 1 |  | 1 |  |  | 1 | 1 |  |  | 1 |  |  |
| 199 | 1 | 3 | 2 | 3 |  | 1 |  |  | 1 |  |  |  | 1 |  |  |  |  |  | 1 |  |  | 1 |  |  |  |  | 1 |  |  | 1 | 1 |  |  |  | 1 | 1 |  |  |  |  |  | 1 |  | 1 |  |  |  |  |  |  | 1 |  |  |  | 1 |  |  | 1 |  |  |  | 1 |  |  |  |  |  | 1 |  |  | 1 |  |  |  | 1 |  |  | 1 |  |  |  |  |  |  | 1 |  |  | 1 |  |  |  | 1 |  |  |  | 1 |  |  | 1 | 1 |  |  | 1 | 1 |  |  |  |  |  |  | 1 |  | 1 |  |  |  |  | 1 | 1 | 1 |  |  |  | 1 |  |  | 1 |  |  |  |  |  | 1 |  |  | 1 |  |  |  | 1 |  |  |
| 200 | 1 | 3 | 1 | 1 |  |  | 1 |  |  |  |  | 1 |  |  |  |  | 1 | 1 |  |  |  |  |  |  | 1 |  |  |  | 1 |  | 1 |  |  |  | 1 |  |  |  |  |  | 1 |  |  |  |  |  | 1 |  |  |  | 1 |  |  |  |  |  | 1 |  |  | 1 |  | 1 |  | 1 |  |  |  |  |  | 1 | 1 |  |  | 1 |  |  |  | 1 |  |  |  |  |  |  | 1 |  | 1 |  |  |  |  | 1 |  |  | 1 |  |  |  |  | 1 |  |  |  |  |  |  |  |  |  | 1 |  |  | 1 |  |  |  |  | 1 |  | 1 |  |  |  | 1 |  |  | 1 |  |  |  | 1 |  | 1 |  |  | 1 |  |  |  | 1 |  |  |
| 201 | 1 | 4 | 1 | 1 | 1 |  |  |  | 1 |  |  |  |  |  |  | 1 |  | 1 |  |  |  |  | 1 |  |  |  |  |  | 1 |  | 1 |  |  | 1 | 1 |  |  |  |  |  |  | 1 |  |  |  |  | 1 |  | 1 |  | 1 |  |  |  |  |  | 1 |  |  | 1 |  |  |  | 1 |  |  |  |  |  | 1 | 1 |  |  | 1 |  |  |  | 1 |  |  |  | 1 |  |  | 1 |  | 1 |  |  |  |  | 1 |  |  | 1 |  |  |  | 1 | 1 |  |  | 1 | 1 |  |  |  |  |  |  | 1 |  | 1 |  |  |  | 1 | 1 |  | 1 |  |  |  | 1 |  |  | 1 |  |  |  |  |  | 1 |  |  | 1 |  |  |  | 1 |  |  |
| 202 | 1 | 1 | 1 | 1 |  | 1 |  |  | 1 |  |  |  |  |  |  | 1 |  | 1 |  |  |  |  |  | 1 |  |  |  |  | 1 | 1 | 1 |  |  |  | 1 |  |  |  |  |  |  | 1 |  |  |  |  | 1 |  | 1 |  |  |  |  |  | 1 |  |  | 1 |  |  |  | 1 |  |  |  |  |  |  |  | 1 | 1 |  |  |  | 1 |  |  | 1 |  |  |  |  |  |  | 1 |  | 1 |  |  |  |  | 1 |  |  |  |  |  | 1 |  | 1 |  |  |  | 1 |  |  |  |  |  |  | 1 |  | 1 |  |  |  |  | 1 |  | 1 |  |  |  | 1 |  |  | 1 |  |  |  | 1 |  |  |  |  | 1 |  |  |  | 1 |  |  |
| 203 | 2 | 4 | 4 | 1 |  |  | 1 | 1 |  |  |  |  |  |  |  |  | 1 | 1 |  |  |  |  |  | 1 |  |  |  |  | 1 |  |  |  |  | 1 | 1 |  |  |  |  |  |  |  | 1 |  |  |  | 1 |  |  |  |  | 1 |  |  |  |  | 1 |  |  | 1 |  | 1 |  |  |  |  |  | 1 |  |  | 1 |  |  | 1 |  |  |  | 1 |  |  |  | 1 |  |  |  |  | 1 |  |  |  |  | 1 |  |  |  |  | 1 |  | 1 | 1 |  |  |  | 1 |  |  |  |  |  | 1 |  |  |  |  | 1 |  | 1 | 1 |  |  |  |  |  | 1 |  |  |  | 1 |  |  | 1 |  |  |  |  | 1 |  |  |  | 1 |  |  |
| 204 | 1 | 4 | 1 | 1 |  | 1 |  |  | 1 |  |  |  |  |  |  | 1 |  | 1 |  |  |  |  | 1 |  |  |  |  |  | 1 | 1 | 1 |  |  | 1 | 1 |  |  | 1 |  |  |  | 1 |  |  |  |  | 1 | 1 | 1 | 1 | 1 |  |  |  | 1 | 1 |  | 1 |  |  |  | 1 |  | 1 |  |  | 1 | 1 |  |  | 1 |  |  |  | 1 |  |  | 1 |  |  |  |  |  |  | 1 |  | 1 |  |  |  |  | 1 |  |  | 1 |  |  |  | 1 | 1 |  |  | 1 | 1 |  |  |  |  |  |  | 1 |  | 1 |  |  |  | 1 | 1 | 1 | 1 |  |  |  | 1 | 1 |  | 1 |  |  |  | 1 |  | 1 |  |  | 1 | 1 |  |  | 1 |  |  |
| 205 | 1 | 3 | 2 | 1 |  | 1 |  |  | 1 |  |  |  |  |  |  | 1 |  |  | 1 |  |  |  |  | 1 |  |  | 1 |  |  | 1 |  |  |  | 1 | 1 |  |  |  |  |  | 1 |  |  |  |  |  | 1 |  |  |  | 1 |  |  |  | 1 | 1 |  | 1 |  |  |  | 1 |  |  |  |  | 1 | 1 |  |  | 1 |  |  |  | 1 |  |  | 1 |  |  |  | 1 |  |  |  |  | 1 |  |  |  |  | 1 |  |  |  | 1 |  |  | 1 | 1 |  |  | 1 | 1 |  |  |  |  |  |  | 1 |  |  |  | 1 |  | 1 | 1 |  | 1 |  |  |  | 1 |  |  | 1 |  |  |  | 1 |  | 1 |  |  | 1 | 1 |  |  | 1 |  |  |
| 206 | 2 | 4 | 2 | 4 | 1 |  |  |  | 1 |  |  |  |  |  |  | 1 |  |  | 1 |  |  |  | 1 |  |  |  |  | 1 |  |  | 1 |  |  | 1 |  |  |  |  |  |  | 1 |  |  |  | 1 | 1 |  |  | 1 | 1 | 1 |  |  |  | 1 | 1 |  | 1 |  |  |  | 1 |  | 1 |  |  | 1 |  |  |  | 1 |  |  |  | 1 |  |  | 1 |  |  |  | 1 |  |  |  |  | 1 |  |  |  |  | 1 |  |  |  | 1 |  |  | 1 | 1 |  |  | 1 | 1 |  |  |  |  |  |  | 1 |  |  |  | 1 |  | 1 | 1 |  | 1 |  |  |  | 1 |  |  | 1 |  |  |  | 1 |  | 1 |  |  | 1 | 1 |  |  | 1 |  |  |
| 207 | 1 | 2 | 2 | 1 |  | 1 |  |  | 1 |  |  |  |  |  |  | 1 |  | 1 |  |  |  |  |  | 1 |  |  | 1 |  |  | 1 | 1 |  |  | 1 | 1 |  |  |  |  |  | 1 | 1 |  |  |  |  | 1 |  | 1 |  | 1 |  |  |  | 1 |  |  | 1 |  |  |  | 1 |  | 1 |  |  |  | 1 |  |  | 1 |  |  |  | 1 |  |  | 1 |  |  |  | 1 |  |  |  |  | 1 |  |  |  |  | 1 |  |  |  | 1 | 1 |  | 1 | 1 |  |  | 1 | 1 | 1 |  |  |  |  | 1 | 1 |  |  |  | 1 |  | 1 | 1 | 1 | 1 |  |  |  | 1 | 1 |  | 1 |  |  |  | 1 |  | 1 |  |  | 1 | 1 |  |  | 1 |  |  |
| 208 | 2 | 3 | 2 | 1 | 1 |  |  |  | 1 |  |  |  |  |  |  | 1 |  | 1 |  |  |  |  | 1 |  |  |  | 1 |  |  |  | 1 |  |  | 1 | 1 | 1 |  |  |  | 1 |  |  |  | 1 |  |  |  |  | 1 |  | 1 |  |  |  |  | 1 |  | 1 |  |  |  | 1 |  | 1 |  |  | 1 | 1 |  |  | 1 |  |  |  | 1 |  |  | 1 |  |  |  | 1 |  |  |  |  | 1 |  |  |  |  | 1 |  |  |  | 1 |  |  | 1 | 1 |  |  | 1 | 1 | 1 |  |  |  |  | 1 |  |  |  |  | 1 |  | 1 | 1 | 1 | 1 |  |  |  | 1 | 1 |  | 1 |  |  |  | 1 |  | 1 |  |  | 1 | 1 |  |  | 1 |  |  |
| 209 | 2 | 2 | 4 | 2 |  | 1 |  |  | 1 |  |  |  |  |  |  | 1 |  | 1 |  |  |  |  |  | 1 |  |  | 1 |  |  |  | 1 |  |  | 1 | 1 |  |  |  |  |  |  | 1 |  |  |  |  | 1 | 1 | 1 |  | 1 |  |  |  | 1 |  |  | 1 |  |  |  | 1 |  | 1 |  |  |  | 1 | 1 |  | 1 |  |  |  | 1 |  |  | 1 |  |  |  | 1 |  |  |  |  | 1 |  |  |  |  | 1 |  |  |  | 1 | 1 |  | 1 | 1 |  |  | 1 | 1 | 1 |  |  |  |  | 1 | 1 |  |  |  | 1 |  | 1 | 1 | 1 | 1 |  |  |  | 1 | 1 |  | 1 |  |  |  | 1 |  | 1 |  |  | 1 | 1 |  |  | 1 |  |  |
| 210 | 1 | 3 | 2 | 1 | 1 |  |  |  | 1 |  |  |  |  |  |  | 1 |  | 1 |  |  |  |  |  | 1 |  |  |  | 1 |  | 1 | 1 |  |  | 1 | 1 |  |  |  |  |  |  | 1 |  | 1 |  |  |  |  | 1 |  | 1 |  |  |  | 1 |  |  | 1 |  |  |  | 1 |  | 1 |  |  | 1 | 1 |  |  | 1 |  |  | 1 |  |  |  | 1 |  |  |  | 1 |  |  | 1 |  | 1 |  |  |  |  | 1 |  |  |  | 1 |  |  | 1 | 1 |  |  | 1 | 1 |  |  |  |  |  |  | 1 |  |  |  | 1 |  |  | 1 |  | 1 |  |  |  | 1 |  |  | 1 |  |  |  | 1 |  | 1 |  |  | 1 | 1 |  |  | 1 |  |  |
| 211 | 1 | 3 | 2 | 3 |  | 1 |  |  | 1 |  |  |  |  |  |  | 1 |  | 1 |  |  |  |  | 1 |  |  |  |  |  | 1 | 1 | 1 |  |  | 1 | 1 |  |  |  |  |  |  | 1 |  |  |  | 1 |  |  | 1 |  | 1 |  |  |  | 1 |  |  | 1 |  |  |  | 1 |  |  |  |  |  |  | 1 |  | 1 |  |  |  | 1 |  |  | 1 |  |  |  | 1 |  |  |  |  | 1 |  |  |  |  | 1 |  |  |  | 1 |  |  | 1 | 1 |  |  | 1 | 1 |  |  |  |  |  |  | 1 |  |  |  | 1 |  | 1 | 1 |  | 1 |  |  |  | 1 |  |  | 1 |  |  |  | 1 |  | 1 |  |  | 1 | 1 |  |  | 1 |  |  |
| 212 | 1 | 2 | 1 | 1 |  | 1 |  |  | 1 |  |  |  |  |  |  | 1 |  |  | 1 |  |  |  | 1 |  |  |  |  | 1 |  | 1 | 1 |  |  | 1 |  | 1 |  |  |  |  | 1 |  |  | 1 |  |  |  |  | 1 |  | 1 |  |  |  | 1 |  |  | 1 |  |  |  | 1 |  | 1 |  |  | 1 |  |  |  | 1 |  |  |  | 1 |  |  | 1 |  |  |  | 1 |  |  |  |  |  | 1 |  |  |  | 1 |  |  | 1 |  |  |  | 1 |  |  |  |  |  |  |  |  | 1 |  | 1 |  |  |  | 1 | 1 |  | 1 |  |  | 1 |  | 1 | 1 |  |  |  | 1 |  |  |  | 1 |  |  |  |  | 1 |  |  |  | 1 |  |  |
| GEN: Gender (1: Male; 2: Female) |  |  |  |  |  |  |  |  |  |  |  |  |  |  |  |  |  |  |  |  |  |  |  |  |  |  |  |  |  |  |  |  |  |  |  |  |  |  |  |  |  |  |  |  |  |  |  |  |  |  |  |  |  |  |  |  |  |  |  |  |  |  |  |  |  |  |  |  |  |  |  |  |  |  |  |  |  |  |  |  |  |  |  |  |  |  |  |  |  |  |  |  |  |  |  |  |  |  |  |  |  |  |  |  |  |  |  |  |  |  |  |  |  |  |  |  |  |  |  |  |  |  |  |  |  |  |  |  |  |  |  |  |  |  |  |  |  |  |  |  |  |  |
| AGE: Age (1: less than 15 years old; 2: 16 – 30 years old; 3: 31 – 45 years old; 4: 46 – 60 years old; 5: > 61 years old) | | | | | | | | | | | | | | | | | | | | | | | | | |  |  |  |  |  |  |  |  |  |  |  |  |  |  |  |  |  |  |  |  |  |  |  |  |  |  |  |  |  |  |  |  |  |  |  |  |  |  |  |  |  |  |  |  |  |  |  |  |  |  |  |  |  |  |  |  |  |  |  |  |  |  |  |  |  |  |  |  |  |  |  |  |  |  |  |  |  |  |  |  |  |  |  |  |  |  |  |  |  |  |  |  |  |  |  |  |  |  |  |  |  |  |  |  |  |  |  |  |  |  |  |  |  |  |  |  |  |
| EDU: Level of education (1: Compulsory school; 2: High School; 3: Bachelor degree; 4: Master degree; 5: Doctor of Phylosophy) | | | | | | | | | | | | | | | | | | | | | | | | | | | | |  |  |  |  |  |  |  |  |  |  |  |  |  |  |  |  |  |  |  |  |  |  |  |  |  |  |  |  |  |  |  |  |  |  |  |  |  |  |  |  |  |  |  |  |  |  |  |  |  |  |  |  |  |  |  |  |  |  |  |  |  |  |  |  |  |  |  |  |  |  |  |  |  |  |  |  |  |  |  |  |  |  |  |  |  |  |  |  |  |  |  |  |  |  |  |  |  |  |  |  |  |  |  |  |  |  |  |  |  |  |  |  |  |  |
| EXP: Diving qualification (1: None; 2: Open Water Diver; 3: Advanced Open Water Diver; 4: Rescue Diver; 5: Divemaster; 6: Instrucotor) | | | | | | | | | | | | | | | | | | | | | | | | | | | | | | |  |  |  |  |  |  |  |  |  |  |  |  |  |  |  |  |  |  |  |  |  |  |  |  |  |  |  |  |  |  |  |  |  |  |  |  |  |  |  |  |  |  |  |  |  |  |  |  |  |  |  |  |  |  |  |  |  |  |  |  |  |  |  |  |  |  |  |  |  |  |  |  |  |  |  |  |  |  |  |  |  |  |  |  |  |  |  |  |  |  |  |  |  |  |  |  |  |  |  |  |  |  |  |  |  |  |  |  |  |  |  |  |
